# DOPAL initiates αSynuclein-dependent impaired proteostasis and degeneration of neuronal projections in Parkinson’s disease

**DOI:** 10.1038/s41531-023-00485-1

**Published:** 2023-03-25

**Authors:** Anna Masato, Nicoletta Plotegher, Francesca Terrin, Michele Sandre, Gaia Faustini, Andrea Thor, Stephen Adams, Giulia Berti, Susanna Cogo, Federica De Lazzari, Camilla Maria Fontana, Paul Anthony Martinez, Randy Strong, Rina Bandopadhyay, Marco Bisaglia, Arianna Bellucci, Elisa Greggio, Luisa Dalla Valle, Daniela Boassa, Luigi Bubacco

**Affiliations:** 1 Department of Biology, University of Padova, Padova, 35131 Italy; 2 Centro Studi per la Neurodegenerazione (CESNE), University of Padova, Padova, Italy; 3 Department of Neuroscience, University of Padova, Padova, 35131 Italy; 4 Department of Molecular and Translational Medicine, University of Brescia, Brescia, 25123 Italy; 5 Department of Neurosciences, University of California San Diego, La Jolla, CA 92093-0608 USA; 6 National Center for Microscopy and Imaging Research, University of California San Diego, La Jolla, CA 92093-0608 USA; 7 Department of Pharmacology, University of California San Diego, La Jolla, CA 92093-0608 USA; 8 Department of Pharmacology and Barshop Institute for Longevity and Aging Studies, University of Texas Health Science Center at San Antonio, San Antonio, TX 78229 USA; 9 South Texas Veterans Health Care Network, San Antonio, TX 78229 USA; 10 Reta Lila Weston Institute of Neurological Studies, UCL Queen Square Institute of Neurology, London, WC1N 1PJ UK

**Keywords:** Parkinson's disease, Cellular neuroscience

## Abstract

Dopamine dyshomeostasis has been acknowledged among the determinants of nigrostriatal neuron degeneration in Parkinson’s disease (PD). Several studies in experimental models and *postmortem* PD patients underlined increasing levels of the dopamine metabolite 3,4-dihydroxyphenylacetaldehyde (DOPAL), which is highly reactive towards proteins. DOPAL has been shown to covalently modify the presynaptic protein αSynuclein (αSyn), whose misfolding and aggregation represent a major trait of PD pathology, triggering αSyn oligomerization in dopaminergic neurons. Here, we demonstrated that DOPAL elicits αSyn accumulation and hampers αSyn clearance in primary neurons. DOPAL-induced αSyn buildup lessens neuronal resilience, compromises synaptic integrity, and overwhelms protein quality control pathways in neurites. The progressive decline of neuronal homeostasis further leads to dopaminergic neuron loss and motor impairment, as showed in in vivo models. Finally, we developed a specific antibody which detected increased DOPAL-modified αSyn in human striatal tissues from idiopathic PD patients, corroborating the translational relevance of αSyn-DOPAL interplay in PD neurodegeneration.

## Introduction

The morphological, functional, and molecular features of the *Substantia Nigra pars compacta* (SNpc) dopaminergic neurons define the uniqueness of this neuronal subpopulation and its preferential vulnerability in Parkinson’s disease (PD)^1,2^. In addition to their autonomous pacemaking activity, dopaminergic neurons present complex arborizations of axonal projections, ensuring a profuse number of striatal synaptic connections, whose integrity needs to be preserved through a high bioenergetic supply^3,4^ and an efficient protein turn-over^5^. According to the dying-back hypothesis for PD, synapse dysfunction and loss constitute the early pathological events initiating a progressive retrograde axonal injury, which gradually evolve to neuronal soma degeneration^6^.

Aging is among the prominent pathological factors leading to PD, as it represents the greatest challenge for upholding efficient degradative pathways^7^, thus altering neuronal protein quality control. Moreover, dopamine-induced oxidative stress seems to be paramount in nigrostriatal neuronal dysfunction as it can affect several intersected pathways, which lead to a negative loop of mitochondrial and lysosomal dysfunction, and protein aggregation^8^. At striatal terminals, the misfolding and aggregation of αSynuclein (αSyn) constitutes a driving factor in synaptic derangement^9–11^.

The concept that a dyshomeostasis of catecholamines may lead to endotoxicity has been lately extended to dopamine catabolites, whose altered levels have been measured in autoptic samples and in vivo PD models^12^. Among them, the monoamine oxidase (MAO) dopamine catabolite 3,4-dihydroxyphenylacetaldehyde (DOPAL) is by far the most reactive^13,14^. Although catechol oxidation to quinone species renders dopamine able to modify thiol groups, this is a spontaneous conversion with a slow kinetics when compared to the rate of enzymatic production of DOPAL by MAO^15–17^. The additional presence of the aldehyde moiety in DOPAL exacerbates its reactivity towards proteins^18^, with detrimental outcomes upon accumulation in the intracellular milieu.

The primary site of DOPAL burden is the pre-synaptic terminal, where its buildup is favored by a combination of defective dopamine storage in synaptic vesicles^19^, increased MAO activity with aging^20^, and decreased DOPAL detoxification by the aldehyde dehydrogenase enzymes (ALDH1A1, ALDH2)^14^. Accordingly, transcriptomic and proteomic studies in *post-mortem* PD patients brains, both familial and idiopathic, identified the selective reduced expression of ALDH1A1 among the molecular determinants involved in the preferential susceptibility of SNpc dopaminergic neurons^1,21–23^, thus sustaining that the resulting DOPAL accumulation might be among the driving forces for dopaminergic neuron degeneration.

We previously demonstrated a functional consequence of DOPAL buildup at the pre-synaptic region, which induces a redistribution of synaptic vesicle pools in primary neuronal cultures^24^. This was linked to the generation of DOPAL-triggered annular-shaped αSyn *off-pathway* oligomers that were able to form pores on vesicles membrane. In agreement with other studies, we showed that DOPAL covalently modifies various lysines on αSyn sequence in a Schiff-base reaction between the primary amines and the aldehyde, with a higher and more specific reactivity than catecholamines^23–26^.

Here, we investigated the consequences of DOPAL buildup on neuronal homeostasis, in the light of DOPAL as a trigger of αSyn-mediated neurotoxicity. We observed a DOPAL-induced αSyn accumulation among neuronal compartments and impaired αSyn clearance in primary neuronal cultures. We assessed the differential impact of the αSyn-DOPAL interplay in diverse neuronal districts, revealing altered synaptic integrity, overwhelmed degradative pathways in neuronal projections and reduced axonal arborization. Accordingly, these observations were substantiated in both mouse and zebrafish in vivo models of defective DOPAL detoxification, which exhibited αSyn accumulation, dopaminergic neuron loss, and impaired motor phenotype. Finally, we developed a monoclonal antibody against DOPAL-modified αSyn that showed the significant presence of high-molecular-weight DOPAL-modified αSyn in striatal tissues from *post-mortem* idiopathic PD patients. Hence, our data disclose a novel pathological interplay between αSyn and DOPAL as a key molecular mechanism of enhanced dopaminergic neuron vulnerability in the early events of PD.

## Results

### DOPAL triggers αSynuclein aggregation and affects αSynuclein proteostasis in neurons

**Fig. 1:**
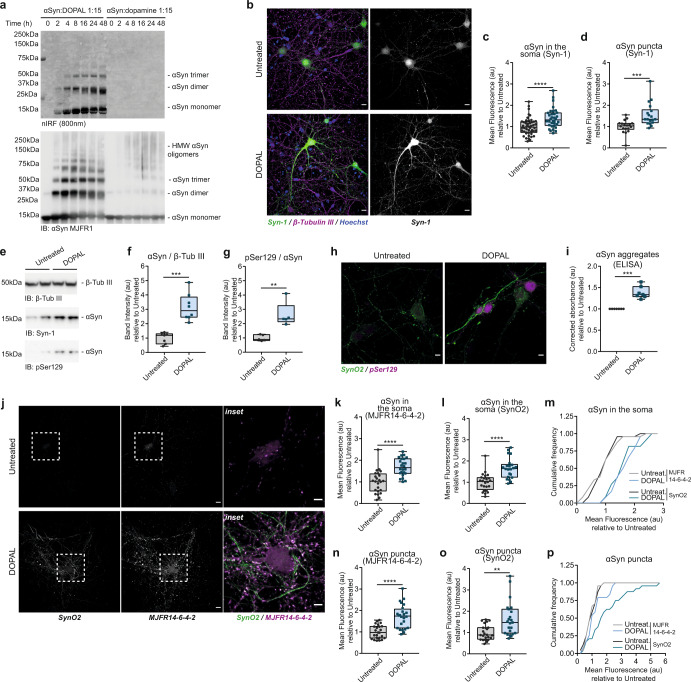
DOPAL affects αSynuclein proteostasis in primary mouse neurons. **a** In vitro aggregation assay of recombinant αSyn incubated with 1:15 DOPAL or dopamine at different time-points, resolved by SDS-Page. The signal derived from oxidized DOPAL covalently bound to αSyn is acquired by nIRF at 800 nm (top panel), whereas total αSyn oligomers are detected by immunoblot (bottom panel). **b**–**p** Imaging and biochemical studies of DOPAL-induced αSyn buildup in untreated and 100 μM DOPAL-treated (for 24 h) wild-type primary mouse cortical neurons. **b** Immunostaining of αSyn (Syn-1, green) and β-Tubulin III (magenta). Nuclei are stained with Hoechst (blue). Scale bar: 10 μm. DOPAL-induced αSyn accumulation is expressed as mean fluorescence intensity **c** in neuronal soma and **d** αSyn-positive puncta. **e**–**g** Monomeric αSyn and pSer129 levels analyzed by western blot. Band intensities are normalized to β-Tubulin III as loading control. Data from three independent cultures are normalized to each untreated sample, pooled together, and analyzed by Mann–Whitney test (**p < 0.01, ***p < 0.001, ****p < 0.0001). **h** Co-immunostaining of aggregated (SynO2) and phosphorylated (pSer129) in the same experimental conditions. Scale bar: 10 μm. **i** Quantification of aggregated αSyn in neuron cell lysates by ELISA expressed as fold change in DOPAL-treated neurons relative to untreated paired samples. Data from seven biological replicates are analyzed by One-sample t-test (***p < 0.001). **j** Co-immunostaining of anti-aggregated αSyn antibodies SynO2 and MJFR14-6-4-2. Scale bar: 10 μm, 5 μm in the inset. αSyn buildup is expressed as mean fluorescence intensity **k**, **l** in neuronal soma and **n**, **o** αSyn-positive puncta. Data from two independent cultures are normalized to each untreated sample, pooled together, and analyzed by Mann–Whitney test (**p < 0.01, ***p < 0.001, ****p < 0.0001). In the same images, cumulative frequency distribution of fluorescence intensities derived from the immunostaining with SynO2 and MJFR14-6-4-2 antibodies measured **m** in the soma and **p** in the synaptic puncta. Data are analyzed by Two-way ANOVA with Tukey’s multiple comparison test: **m** untreated MJFR14-6-4-2/SynO2 vs DOPAL MJFR14-6-4-2/SynO2: ****p < 0.0001; **p** untreated MJFR14-6-4-2 vs DOPAL MJFR14-6-4-2: ns, untreated SynO2 vs DOPAL SynO2: ****p < 0.0001, DOPAL MJFR14-6-4-2 vs DOPAL SynO2: ****p < 0.0001. **c**, **d**, **f**, **g**, **I**, **k**, **l**, **n**, **o** Data are displayed as box and whiskers plot showing the minimum and maximum points (whiskers), the first quartile, median and third quartile (box lines).

Previous findings support the capability of oxidized dopamine to induce αSyn oligomerization by a non-covalent interaction of its catechol group with the _125_YEMPS_129_ motif at the C-terminus of αSyn^27,28^. However, the additional presence of the aldehyde moiety on DOPAL has a diverse and higher reactivity that leads to a covalent and irreversible modification of multiple lysines on αSyn^24,26^. Using an in vitro αSyn aggregation assay following the time-course of the reaction (Fig. 1a), we observed substantial DOPAL-induced covalent modification of αSyn monomers detected by nIRF^29^, and generation of SDS-resistant αSyn oligomers. These species displayed a completely different aggregation pattern as compared to that obtained upon incubation with dopamine, where the pool of monomeric αSyn remains essentially unaltered.

Considering the many potential neurotoxic outcomes associated with the accumulation of αSyn oligomers in the intracellular milieu, we set out to dissect the impact of DOPAL buildup on different processes that regulate αSyn proteostasis, including trafficking, subcellular localization, and clearance in the neuronal environment. To this aim, we employed diverse cellular models exposed to DOPAL treatment, including primary mouse and rat cortical neurons and the human neuroblastoma-derived BE(2)-M17 cells, to single out the effects elicited by DOPAL. Exogenously administered DOPAL was synthetized in our laboratory adapting Fellman’s protocol^30^, which resulted in a compound with 95% purity (Supplementary Fig. 1a–d). The detection of the nIRF signal derived from DOPAL adducts^29^, present in both the detergent-soluble and insoluble fractions of BE(2)-M17 cells following an overnight treatment at increasing DOPAL concentrations, confirmed that DOPAL enters the cells (Supplementary Fig. 1e, f). Furthermore, the specificity of the DOPAL-derived nIRF signal was confirmed by the quenching of DOPAL-mediated protein reactivity when co-treated with an excess of primary amines (i.e., aminoguanidine, AMG) in the cell medium (Supplementary Fig. 1g).

On these premises, we next investigated the consequence of DOPAL exposure on αSyn steady-state levels in mouse primary neurons. Following a 24-h treatment with 100 µM DOPAL, αSyn significantly increased in neuronal cell bodies as well as neurites and synapses (Fig. 1b–d, Supplementary Fig. 2a, b), here identified as the scattered puncta detected in the field of view (see Fig. 3g for co-localization with the pre-synaptic marker VAMP2). αSyn elevation also correlated with an increased fraction of protein phosphorylation at serine 129 (pSer129), a recognized hallmark associated to αSyn pathological species, across different cellular models (Fig. 1e–h, Supplementary Fig. 2c–g). Moreover, we performed an ELISA assay on neuron lysates using the anti-aggregated αSyn (MJFR14-6-4-2) antibody to confirm the presence of αSyn multimers (cell lysate from primary astrocytes was used as negative control in the essay). In all the tested samples, we measured a systematic increase in αSyn aggregates in DOPAL-treated neurons as compared to the untreated paired samples, in the range of 10–40 pg of αSyn/μg of total proteins (Fig. 1i, Supplementary Fig. 2h–j). Consistently, immunostaining with the conformation-specific αSyn SynO2 and MJFR14-6-4-2 antibodies (which displayed a similar neuronal signal distribution as compared to the Syn-1 antibody staining) independently confirmed the DOPAL-induced accumulation of αSyn multimeric species both in the cell bodies and synaptic puncta (Fig. 1j–o, Supplementary Fig. 2k–m). Both SynO2 and MJFR14-6-4-2 antibodies display good affinity towards both oligomeric and aggregated αSyn, being generated by different immunogens^31^. Of note, the immunostaining with the two antibodies highly correlated in the detection of αSyn species, both in the analysis of the total fluorescence in the field of view and locally in the soma, where both oligomers and fibrillar aggregated forms accumulate (Fig. 1m, Supplementary Fig. 2n, o). When specifically analyzing the distribution of the fluorescence intensities at the synaptic puncta, the signal increase was observed only with the SynO2 immunostaining in DOPAL-treated neurons, overall suggesting an accumulation of peculiar αSyn aggregates specifically at synapses (Fig. 1p).

**Fig. 2:**
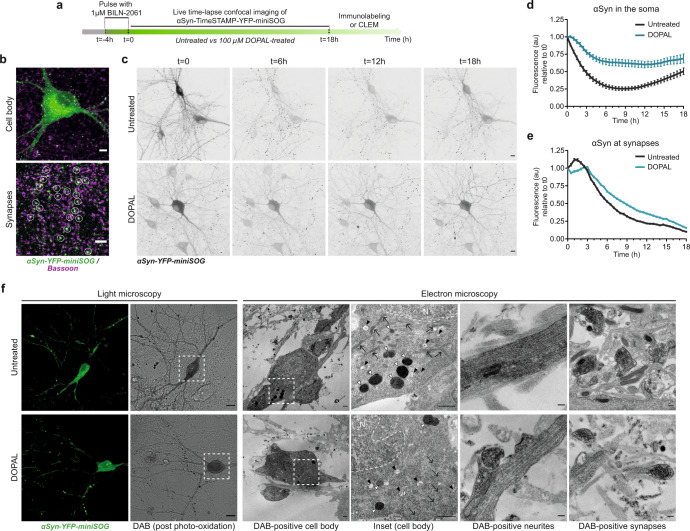
DOPAL impacts αSynuclein subcellular distribution in primary rat neurons. **a** Schematic representation of the experimental setup of the pulse-chase experiment in αSyn-TimeSTAMP-YFP-miniSOG overexpressing primary rat cortical neurons. See also Fig. S3. **b** Illustrative immunofluorescence images showing the αSyn-YFP-miniSOG localization in the cell body (green, upper image) and in the peripheral synapses, where it co-localizes with the pre-synaptic marker Bassoon (magenta, bottom image, white circles). Scale bar: 5 μm. **c** Snapshots at different time points of live time-lapse confocal imaging in untreated and 100 μM DOPAL-treated neurons. Scale bar: 10 μm. **d**, **e** Quantification of the YFP fluorescence variations in the soma and at synapses, where the fluorescence intensity of each cell body/synapse is normalized to *t* = 0. Data are pooled together from three independent experiments (untreated: 41 cell bodies, 3320 puncta; DOPAL-treated: 38 cell bodies, 3712 puncta), shown as mean ± SEM each time-point, and analyzed by Two-way ANOVA with Bonferroni’s multiple comparison test: **d** interaction ****p < 0.0001, treatment ****p < 0.0001; **e** interaction ****p < 0.0001, treatment ****p < 0.0001. **f** CLEM of rat primary cortical neurons expressing αSyn-TimeSTAMP-YFP-miniSOG, at *t* = 24 h after BILN-2061 pulse. Photo-oxidation in untreated and 100 μM DOPAL-treated neurons (scale bar: 10 μm) and representative electron micrographs of the DAB-positive cell bodies (scale bar: 1 μm), neurites and pre-synaptic terminals (scale bar: 200 nm). N: nucleus; arrows: mitochondria; black arrowheads: MVBs; white arrowheads: lysosomes.

To gain insights into the mechanisms and consequences of DOPAL-modified αSyn accumulation, we next evaluated the kinetics of αSyn trafficking and subcellular distribution upon DOPAL treatment. We performed a pulse-chase experiment coupled to live-cell time-lapse confocal imaging by applying a CLEM approach using the αSyn-TimeSTAMP-YFP-miniSOG probe (Time – Specific Tag for the Age Measurement of Proteins)^32,33^ in primary rat neurons (Fig. 2a, Supplementary Fig. 3a). Briefly, the application of 1 µM BILN-2061 (here for 4 h) induced the YFP-miniSOG fluorescent labeling of the newly produced αSyn, which progressively distributed within the soma where the protein is synthetized, then along the neurites and in the periphery, where it co-localized with the pre-synaptic scaffold protein Bassoon (Fig. 2b). Following BILN-2061 wash-out, live-cell time-lapse confocal imaging allowed to study the trafficking of the fluorescent αSyn subpopulation along the cell body and the synaptic boutons by analyzing the changes in the YFP-miniSOG signal over time (18-h time-course) in the untreated condition or in the presence of 100 µM DOPAL (Fig. 2c–e, Supplementary Files 2–3). In the first 8 h, untreated neurons exhibited a rapid 75% decrease of the fluorescence signal of αSyn-YFP-miniSOG in the soma due to the progressive outward trafficking to the pre-synaptic terminals, that was followed by a progressive rerise to 50% in the following 10 h (Fig. 2d). Consistently, the fluorescence variations in the peripheral terminals indicated that, in these cells, the new fluorescent αSyn arriving from the soma quickly increased at the synapse showing a peak within the first 3 h, which was followed by a progressive decrease (Fig. 2e). This trend is consistent with a combination of local clearance of αSyn both in the soma and the processes, together with a retrograde trafficking from the synapses^34^. Conversely, DOPAL-treated neurons exhibited a slower and less pronounced decrease of the fluorescence signal in the soma over time (Fig. 2d), and no increase in fluorescence within the first 3 h at synaptic terminals, where the subsequent decrease of the protein was delayed as compared to untreated cells (Fig. 2e). These results indicate that DOPAL-treated neurons exhibited impaired αSyn trafficking as well as a less efficient degradation, as only 30% of the soma signal disappeared within the 18 h analyzed.

### DOPAL-induced αSynuclein buildup affects synapse integrity

To understand the consequences of DOPAL buildup on αSyn subcellular localization and the organization of the neuronal ultrastructure, we exploited the miniSOG tag to perform correlated light and electron microscopy (CLEM) studies^35^ in untreated and DOPAL-treated neurons at *t* = 24 h post BILN-2061 pulse-chase (Fig. 2a). The darker signal of the polymerized DAB imaged by EM reflected αSyn labeling and revealed its presence in the soma, in the neurites and in association with the membrane of synaptic vesicles (Fig. 2f), as previously described^36^.

**Fig. 3:**
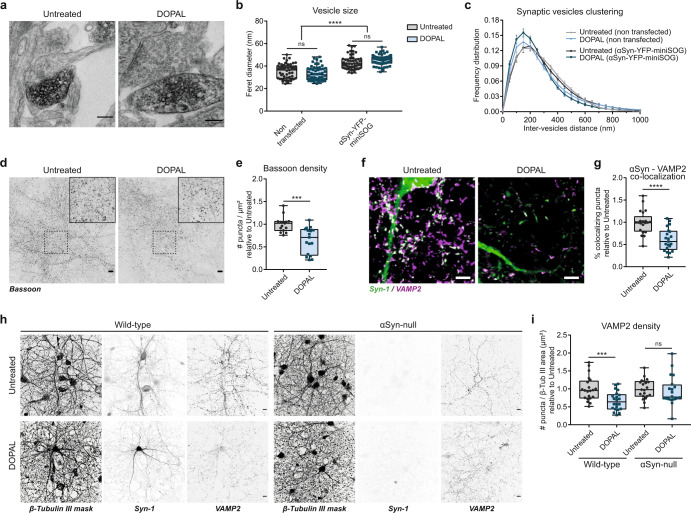
DOPAL-induced αSynuclein buildup affects synaptic integrity. **a** In the CLEM experiment in Fig. 2f, representative electron micrographs of pre-synaptic terminals in photo-oxidized areas showing αSyn-miniSOG-positive terminals in close proximity to terminals from non-transfected neurons. Scale bar: 200 nm. **b** Quantification of synaptic vesicles mean size measured as Feret diameter (nm) and **c** frequency distribution of synaptic vesicles clustering expressed as inter-vesicles distance (nm). Data are pooled from two independent experiments, three photo-oxidized areas (non-transfected _ untreated: 51 synapses; non-transfected _ DOPAL-treated: 54 synapses; αSyn-miniSOG-positive _ untreated: 49 synapses; αSyn-miniSOG-positive _ DOPAL-treated: 59 synapses), and analyzed by Two-way ANOVA: **b** αSyn-overexpression ****p < 0.0001, treatment *p* > 0.05; **c** interaction ****p < 0.001, αSyn-overexpression *p* > 0.05. **d** Immunostaining of Bassoon in untreated and 100 μM DOPAL-treated (for 24 h) primary rat cortical neurons. Scale bar: 10 µm. **e** Relative quantification of Bassoon-positive puncta per area unit (µm^2^). **f** Immunostaining of αSyn (green) and VAMP2 (magenta) in untreated and 100 μM DOPAL-treated (for 24 h) wild-type primary mouse cortical neurons. Scale bar: 5 µm. **g** Relative quantification of the percentage of αSyn-positive puncta colocalizing with VAMP2. **h** Immunostaining of β-Tubulin III (converted to binary mask), αSyn, and VAMP2 in wild-type and αSyn-null primary mouse cortical neurons with the same treatments. Scale bar: 10 µm. **i** The synaptic density is expressed as number of VAMP2-positive puncta normalized to the β-Tubulin III area (µm^2^). Data from **e** three and **g**, **i** four independent cultures are normalized to each untreated sample, pooled together, and analyzed by Mann–Whitney test (***p < 0.001, ****p < 0.0001). **b**, **e**, **g**, **i** Data are displayed as box and whiskers plot showing the minimum and maximum points (whiskers), the first quartile, median and third quartile (box lines). **c** Data are shown as mean ± SEM each point.

We first studied the pre-synaptic terminals, where αSyn is thought to exert its physiological function^37,38^. As αSyn has been reported to modulate the dynamics and the membrane curvature of synaptic vesicles^9,39^, we compared the size and clustering of vesicles in the synapses of non-transfected and αSyn-YFP-miniSOG expressing neurons (Fig. 3a). The measurement of synaptic vesicle size revealed an αSyn overexpression-driven increase in vesicle diameter (untreated neurons: non-transfected 35.3 ± 0.9 nm; αSyn-overexpressing 43.3 ± 0.9 nm). Interestingly, while DOPAL treatment (DOPAL-treated: non-transfected neurons 33.3 ± 0.9 nm; αSyn-overexpressing 45.4 ± 0.6 nm) did not significantly affect synaptic vesicle size (Fig. 3b), it did influence to some extent the synaptic vesicle clustering by shortening the inter-vesicle distance in an αSyn-dependent manner (Fig. 3c).

To further test the impact of DOPAL on pre-synaptic structures, we assessed synaptic density by immunofluorescence, detecting a considerably reduced number of Bassoon-positive puncta in primary rat neurons after 24-h of 100 µM DOPAL treatment (Fig. 3d, e). Under the same treatment condition, a DOPAL-induced synaptic loss of comparable magnitude was independently confirmed by the analysis of the VAMP2-positive puncta density in primary mouse neurons. Also, the co-localization of αSyn peripheral puncta with this pre-synaptic marker showed a similar decrease (Fig. 3f, g). Notably, the DOPAL-induced synaptic loss relied on the presence of αSyn, as a non-significant reduction in VAMP2-positive puncta was observed in primary neurons isolated from mice with αSyn-null background (Fig. 3h, i). Moreover, we verified that the effect on synaptic density was not related to a DOPAL-induced cell loss, as showed by the analysis of the number of nuclei per area unit (Supplementary Fig. 3b, c).

Overall, the data indicate that DOPAL-induced αSyn accumulation affects the synaptic integrity, altering vesicle organization and reducing synapse density.

### DOPAL affects αSynuclein turn-over and promotes αSynuclein accumulation in endo-lysosomal pathway

Next, we sought to investigate the impact of DOPAL on αSyn turn-over, considering that in vitro DOPAL covalent modification of αSyn generates monomeric and oligomeric species that were more resistant to limited proteolysis by Proteinase K (PK) (Supplementary Fig. 4a, b).

To assess whether DOPAL affects αSyn clearance also in the cellular environment, we performed a pulse-chase experiment using the HaloTag labeling technology (transient overexpression of αSyn-HaloTag construct) in BE(2)-M17 cells. Following an overnight 100 µM DOPAL treatment and a 3-h pulse with 5 µM biotin ligand^40^, αSyn-HaloTag was isolated by pull-down with streptavidin-coated beads at different time points (Supplementary Fig. 4c). Here, DOPAL treatment resulted in consistent accumulation of both oligomeric and monomeric species, also phosphorylated at Ser129, which were not fully degraded after 30 h of time-course (Supplementary Fig. 4d–f).

**Fig. 4:**
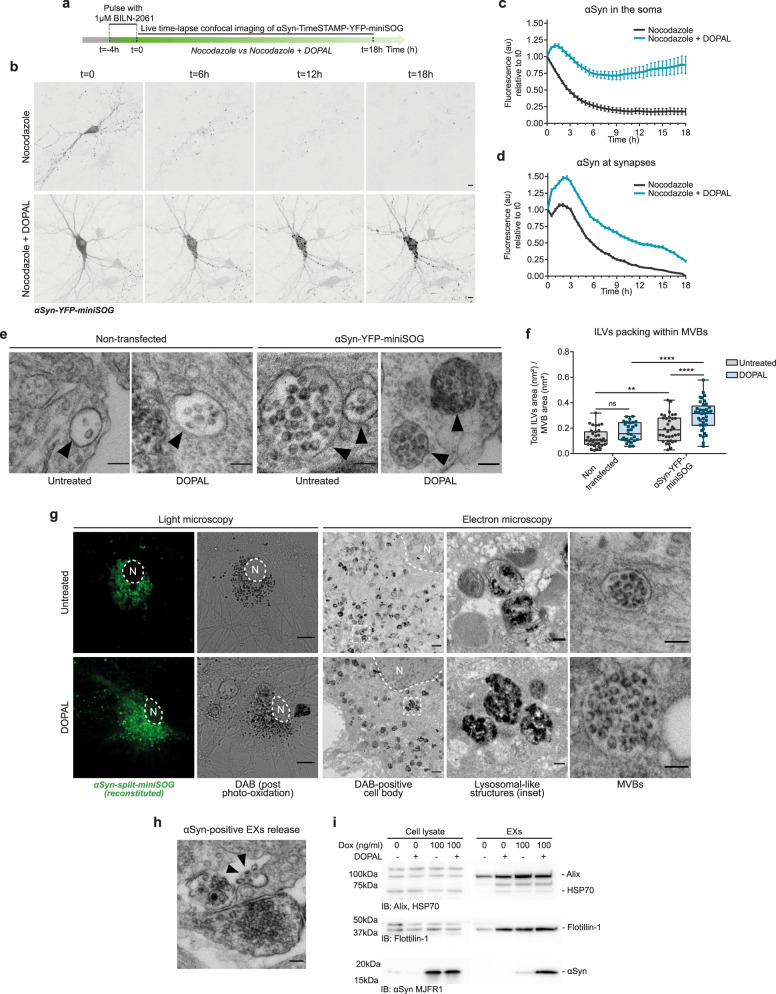
DOPAL promotes αSynuclein accumulation in the endo-lysosomal pathway and exosomal spreading. **a** Schematic representation of the pulse-chase experiment in αSyn-TimeSTAMP-YFP-miniSOG-overexpressing primary rat cortical neurons in the presence of 5 µg/ml Nocodazole in the cell medium, −/+100 µM DOPAL, and **b** representative snapshots at different time points. Scale bar: 10 μm. **c**, **d** Quantification of the αSyn fluorescence variations in the soma and at synapses, where the fluorescence intensity of each cell body/synapse is normalized to *t* = 0. Data are pooled together from three independent experiments (Nocodazole: 32 cell bodies, 2154 puncta; Nocodazole + DOPAL: 23 cell bodies, 2600 puncta), shown as mean ± SEM each time-point, and analyzed by Two-way ANOVA with Bonferroni’s multiple comparison test: **c** interaction ****p < 0.0001, treatment ****p < 0.0001; **d** interaction ****p < 0.0001, treatment ****p < 0.0001. **e** In the CLEM experiment in Fig. 2f, representative electron micrographs of MVBs (indicated by the black arrowheads) in non-transfected and αSyn-miniSOG-positive neurons. Scale bar: 100 nm. **f** Quantification of ILVs density expressed as total area of ILVs occupied in each MVB. Data are pooled from two independent experiments, three photo-oxidized areas (non-transfected _ untreated: 35 MVBs; non-transfected _ DOPAL-treated: 29 MVBs; αSyn-miniSOG-positive _ untreated: 36 MVBs; αSyn-miniSOG-positive _ DOPAL-treated: 37 MVBs) and analyzed by Two-way ANOVA with Sidak’s multiple comparison test (**p < 0.01, ****p < 0.0001). Data are displayed as box and whiskers plot showing the minimum and maximum points (whiskers), the first quartile, median and third quartile (box lines). **g** CLEM of rat primary cortical neurons expressing αSyn-split-miniSOG. Photo-oxidation of reconstituted αSyn-split-miniSOG in untreated and 100 μM DOPAL-treated neurons for 24 h (scale bar: 10 μm), with corresponding electron micrographs of the DAB-positive cell body (N: nucleus; scale bar: 1 μm) and higher magnification of perinuclear lysosomes and MVBs (scale bar: 100 nm). **h** Electron micrograph of MVB fused with the plasma membrane (black arrowheads) and αSyn-miniSOG-positive EXs extracellular release. Scale bar: 100 nm. **i** Immunoblot of cell lysates and exosomal fraction from non-induced and αSyn-overexpressing (100 ng/ml dox) BE(2)-M17-αSyn cells, untreated and 100 μM DOPAL-treated for 48 h. EXs purification was assessed by the enrichment in the exosomal markers Alix, HSP70, and Flotillin-1, while αSyn loading was detected by anti-αSyn MJFR1 antibody.

We then transposed this experiment to the study of αSyn turn-over in primary neurons, with the analysis of an additional level of complexity of subcellular compartmentalization. Hence, we performed pulse-chase live-cell imaging experiments in primary rat neurons and measured the fluorescence signal variations of αSyn-TimeSTAMP-YFP-miniSOG in the presence of Nocodazole^34^, which disrupts the microtubule network to exclude the confounding effect of the axonal trafficking component (Fig. 4a). Under these conditions, the impact of DOPAL on αSyn-YFP-miniSOG fluorescence decay was more pronounced, maintaining the fluorescence variations consistently higher in both the soma and at the synapses when compared to the control condition (Nocodazole only) (Fig. 4b–d, Supplementary Files 4–5). In the control neurons (not exposed to DOPAL), where the fluorescent αSyn-YFP-miniSOG was confined in the soma or in the peripheral terminals by Nocodazole, the protein was gradually degraded reaching 15% of the initial signal in 18 h in the cell body, and it almost completely disappeared in the peripheral terminals. This allowed us to differentially estimate αSyn-YFP-miniSOG half-life in the range of 2.6 ± 0.4 h in the soma and 6.3 ± 0.2 h at the synapses, consistent with a rapid turnover of αSyn-YFP-miniSOG under the efficient neuronal protein quality control. Conversely, in DOPAL-treated neurons, the decrease of αSyn-YFP-miniSOG fluorescence in the soma was considerably lower than in control cells, as the 85% of αSyn-YFP-miniSOG signal was still detected after 18 h in the cell bodies (Fig. 4c). Meanwhile at the synapses, a rapid increase of the fluorescent signal in the first 3 h was possibly due to the clustering of αSyn-YFP-miniSOG in DOPAL-induced aggregates, which appeared to be more resistant to proteolysis as their fluorescence decayed with a slower kinetics as compared to the untreated neurons, down to 20% of the starting protein signal at 18 h (Fig. 4d).

Interestingly, in the CLEM experiments conducted in rat primary neurons using the αSyn-TimeSTAMP-YFP-miniSOG probe, we observed a consistent DAB-derived signal in Multi-Vesicular Bodies (MVBs) in the processes of both untreated and DOPAL-treated conditions, indicating the presence of αSyn in the lumen of Intra-Luminal Vesicles (ILVs) (Fig. 4e). By measuring ILVs density in MVBs (expressed either as number of ILVs per MVB or the fraction of area occupied by ILVs in the MVB lumen), αSyn overexpression appeared to increase ILVs loading as compared to MVBs of non-transfected cells, without affecting MVB size (Supplementary Fig. 4g, h). Moreover, DOPAL treatment significantly increased the αSyn-positive ILVs packing within MVBs, when compared to MVBs in untreated and non-transfected neurons (Fig. 4f), thus highlighting an additive DOPAL effect on MVBs driven by the expression levels of αSyn. Consistent with this, we observed the effect of DOPAL buildup on MVBs formation in a stable and inducible BE(2)-M17 cell line overexpressing αSyn under a dox-inducible promoter (Supplementary Fig. 4i–n). Here, we further demonstrated that the αSyn accumulation due to DOPAL treatment correlated with an increase in MVB-related structures traced by EGFP-tagged CD63, a tetraspanin enriched in ILV membranes (Supplementary Fig. 4k–n).

Based on these observations of αSyn loading in MVBs, we hypothesized the engagement of the endo-lysosomal pathway in the clearance of αSyn aggregated species from the periphery to the soma. To investigate the distribution and accumulation of αSyn oligomers, we implemented a CLEM complementation assay using the αSyn-split-miniSOG probe^41^, which confirmed the labeling of αSyn oligomeric species in MVBs of DOPAL-treated neurons as well as in lysosomal-like compartments in the cell body (Fig. 4g). Accordingly, longer DOPAL treatment (100 µM DOPAL for 48 h) in primary mouse neurons resulted in buildup of aggregated αSyn clusters in the soma and along the neurites in proximity to the cell body, detected by the MJFR14-6-4-2 antibody and quantified as decreased nearest neighbor distance (Nnd) among clusters (Supplementary Fig. 5a, b). Whereas, in untreated neurons, the αSyn staining was still consistent with the physiological distribution of the protein among the soma and the pre-synaptic terminals (sparser puncta) as also observed for the αSyn staining with the Syn-1 antibody (Supplementary Fig. 5c).

Of note, it has been observed that, when the lysosomes are clogged, the fusion of MVBs with the plasma membrane and vesicles release are promoted^42^, thus endorsing the exosomal pathway among the spreading mechanisms for αSyn toxic species^43^. In Fig. 4h, we reported a representative electron micrograph of MVB fusion with the plasma membrane and release of αSyn-positive ILVs in the extracellular space. Hence, we isolated exosomes (EXs) from the cell culture medium of untreated and 100 µM DOPAL-treated BE(2)-M17-αSyn cells, comparing non-induced cells and αSyn-overexpression with 100 ng/ml dox. By monitoring the exosomal markers Alix, HSP70, and Flotillin-1, we observed both a substantial DOPAL- and αSyn-dependent effect in EXs enrichment, as well a significant increase in αSyn loading into EXs derived from αSyn-overexpressing DOPAL-treated cells (Fig. 4i). Also, to evaluate the impact of DOPAL-modified αSyn on EXs, we isolated vesicles secreted from HEK293T cells overexpressing αSyn-EGFP after an overnight 100 µM DOPAL treatment. The presence of αSyn-EGFP, phosphorylated at serine 129, was confirmed by western blot (Supplementary Fig. 5d, e), and a limited PK-proteolysis experiment demonstrates that αSyn-EGFP is loaded within the EXs, being digested only when the EXs membrane was dissolved in the presence 1% Triton-X (Supplementary Fig. 5d). Moreover, EM imaging of isolated vesicles allowed to measure the structural variations induced by the DOPAL-modified αSyn species present in exosomal lumen, namely the increased vesicle size and decreased EXs circularity (Supplementary Fig. 5f–h).

**Fig. 5:**
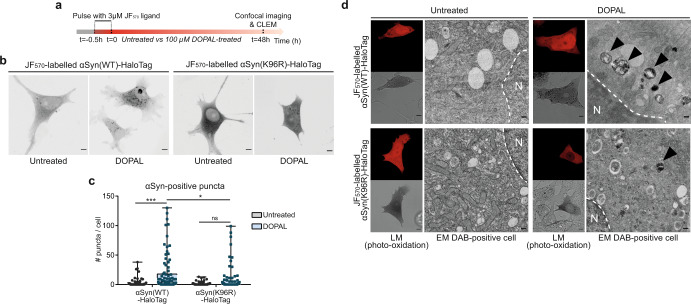
K96 is required for DOPAL-αSynuclein endo-lysosomal degradation. **a** Schematic representation of the pulse-chase experiment using the fluorescent JF_570_ HaloTag ligand in αSyn-HaloTag-overexpressing BE(2)-M17 cells. **b** Representative confocal images of BE(2)-M17 cells expressing αSyn(WT)-HaloTag and αSyn(K96R)-HaloTag, labeled with JF_570_ HaloTag ligand, in untreated and 100 μM DOPAL-treated cells for 48 h. Scale bar: 5 μm. **c** Incidence of αSyn-positive cytoplasmic puncta. Data from three independent experiments are pooled together and analyzed by Two-way ANOVA with Sidak’s multiple comparison test (*p < 0.05, ***p < 0.001). Data are displayed as box and whiskers plot showing the minimum and maximum points (whiskers), the first quartile, median and third quartile (box lines). **d** CLEM of BE(2)-M17 cells expressing αSyn(WT)-HaloTag and αSyn(K96R)-HaloTag, following protocol experiment as in (**a**). LM images (confocal and bright field, scale bar: 5 μm) and corresponding EM micrographs of DAB-positive cells (N: nucleus; scale bar: 200 nm) showing a prevalence of αSyn-positive lysosomal-like structures (black arrows) in DOPAL-treated αSyn(WT)-HaloTag-expressing cells.

To further analyze the molecular mechanism of DOPAL-induced αSyn loading in the endo-lysosomal pathway and clearance of αSyn oligomers via autophagy, we adapted the HaloTag pulse-chase experiment to an imaging approach using the fluorescent JF_570_ ligand^44^. Following a 30-min pulse with 3 µM JF_570_ fluorescent HaloTag ligand to covalently label an αSyn-HaloTag subpopulation (Fig. 5a), 48-h 100 µM DOPAL treatment led to the formation of αSyn-positive cytoplasmic puncta (Fig. 5b, c), which corresponded to αSyn accumulation in lysosomal-like compartments, as detected by CLEM (Fig. 5d). Moreover, knowing that the ubiquitination on lysine 96 by the ubiquitin-ligase Nedd4 is key in targeting αSyn to the endo-lysosomal degradation route^45^, we designed the αSyn(K96R)-HaloTag construct to prevent this specific post-translational modification on αSyn. With these premises, a time-course in BE(2)-M17 expressing αSyn(WT)-HaloTag or αSyn(K96R)-HaloTag in the presence of 50 μM cycloheximide (CHX), an inhibitor of de novo protein synthesis, was designed to study the differential turnover of the two isoforms. While αSyn(WT)-HaloTag displayed a half-life of 18.8 h, the one-phase decay for αSyn(K96R)-HaloTag estimated a shorter half-life of 7.3 h but a plateau at 30% of protein level, thus suggesting an intrinsic reduced degradative capacity of the K96R mutant (Supplementary Fig. 5i, j). Interestingly, reduced αSyn(K96R)-HaloTag cytosolic inclusions and lysosomal accumulation were observed by CLEM in DOPAL-treated cells as compared to αSyn(WT)-HaloTag (Fig. 5b–d), revealing compromised targeting of the mutant αSyn oligomers to the autophagic route, rather remaining diffused in the cytoplasm. These observations were further confirmed by the study of αSyn-HaloTag steady-state levels in BE(2)-M17 cells following DOPAL overnight treatment concomitant to the manipulation of the main degradative pathways, namely proteasome inhibition by 20 μM MG132, autophagy blocking by 50 μM chloroquine and autophagy activation by serum starvation (Supplementary Fig. 5k). Here, αSyn(WT)-HaloTag resulted in significant buildup after proteasome inhibition, suggesting the proteasomal degradation as the preferential pathway for αSyn clearance under physiological conditions. While DOPAL per se induced significant accumulation of the protein, the co-treatment of DOPAL and MG132 did not affect αSyn(WT)-HaloTag levels suggesting the engagement of the autophagic pathway for the degradation of DOPAL-modified αSyn species, as confirmed by starvation which promoted the degradation of a significant fraction of the DOPAL-αSyn (Supplementary Fig. 5l). Instead, both DOPAL treatment and the inhibition of proteasome or autophagy resulted in a significant accumulation of αSyn(K96R)-HaloTag, with starvation failing to facilitate protein clearance in the DOPAL-treated cells (Supplementary Fig. 5m). Collectively, the data indicate that DOPAL affects αSyn turnover and K96 is required for the targeting to endo-lysosomal degradation of DOPAL-modified αSyn species.

### DOPAL and αSynuclein act in concert to hinder neuronal proteostasis

Since we observed that DOPAL promotes αSyn oligomers to be engulfed by the peripheral endosomal systems (Fig. 4), we then aimed at assessing whether an interplay between DOPAL-induced oligomeric αSyn burden and DOPAL-mediated protein modification could progressively affect neuronal proteostasis. We thus studied the levels and spatial distribution of different read-outs of degradative pathways in primary neurons following treatment with 100 µM DOPAL for 24 h.

**Fig. 6:**
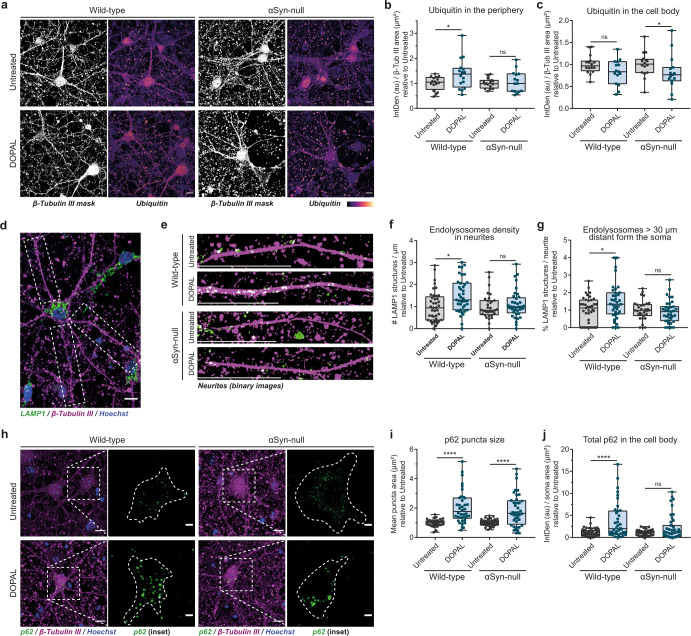
αSynuclein and DOPAL act in concert to hinder neuronal proteostasis. Immunofluorescence analysis of proteostasis markers in untreated and 100 μM DOPAL-treated (for 24 h) wild-type and αSyn-null primary mouse cortical neurons. **a** Immunostaining of β-Tubulin III (converted to binary mask) and Ubiquitin (heatmap). Scale bar: 10 µm. Quantification of the Ubiquitin fluorescence **b** in the periphery and **c** the cell bodies, both normalized to β-Tubulin III area (µm^2^). **d** Immunostaining of β-Tubulin III (magenta) and LAMP1 (green) in primary mouse neurons. Nuclei are stained with Hoechst (blue). The insets provide examples of the criteria for neurite identification for endolysosomal density analysis in neuronal projections. Scale bar: 10 µm. **e** In the enlargement of neurites, both β-Tubulin III and LAMP1 fluorescence signals are converted to binary mask to emphasize the overlay (puncta in white). In the bottom left part of the images, the scale bar corresponds to 30 µm. **f** Endolysosomes density in neurites expressed as number of LAMP1 structures/µm^2^ and **g** percentage of endolysosomes in proximity to the soma (<30 µm) in each neurite. **h** Immunostaining of β-Tubulin III (magenta) and p62 (green). Nuclei are stained with Hoechst (blue). Scale bar: 10 µm. In the inset, the p62 fluorescence signal in the cell body is enlarged and the dotted line defines the soma boundaries (scale bar: 3 µm). **i** Quantification of the p62-positive puncta mean size (µm^2^) in each soma and **j** total p62 signal normalized to the soma area. Data from three independent experiments are normalized to each untreated sample, pooled together, and analyzed by Mann–Whitney test (*p < 0.05, ****p < 0.0001). **b**, **c**, **f**, **g**, **i**, **j** Data are displayed as box and whiskers plot showing the minimum and maximum points (whiskers), the first quartile, median and third quartile (box lines).

First, we evaluated whether DOPAL treatment in the presence (wild-type mouse neurons) or in the absence of αSyn (αSyn-null mouse neurons) equally affects protein ubiquitination, which drives protein quality control through ubiquitin proteasome system (UPS)-operated degradation as well as endosomal protein sorting and selective autophagy. Interestingly, we observed a significant DOPAL-induced accumulation of ubiquitinated proteins only in the periphery of wild-type neurons as opposed to the soma (Fig. 6a–c, Supplementary Fig. 6a), supporting that αSyn synaptic accumulation is the key event driving the engulfment of quality control machineries following DOPAL buildup.

We then assessed whether the increased protein ubiquitination in the neuronal processes correlated with the upregulation of the endosomal pathway to transport aberrant proteins to the lysosomal degradation in the soma, as we observed for the MVBs by CLEM. Consistently, an increased density of lysosomal associated protein1 (LAMP1)-positive endolysosomal structures was observed in the neurites of DOPAL-treated wild-type neurons when compared to the αSyn-null neurons (Fig. 6d–f), without significant difference in the detected organelle size (Supplementary Fig. 6b). Of note, the endolysosomes in DOPAL-treated wild-type neurons accumulated mainly in the distal portion of the neurites (distance >30 µm from the soma, Fig. 6g), supporting our hypothesis of impaired peripheral degradation systems by the DOPAL buildup-induced αSyn accumulation. Conversely, no significant difference in either organelle density or LAMP1-mean fluorescence intensity was observed in the soma endolysosomes, with the sole exception of a decrease in the average size of LAMP1-positive structures in αSyn-null neurons (Supplementary Fig. 6c–f).

In addition to the endosomal pathway, more substrates are targeted to lysosomal degradation via macroautophagy, a system involved in the clearance of misfolded aggregates, where the adapter protein p62 binds ubiquitinated proteins and organelles to be engulfed in the autophagosomes that further fuse with the lysosomes. Interestingly, DOPAL treatment had a marked effect on p62 in the soma, generating larger and brighter puncta as compared to untreated neurons (independently on the genotype), synonymous of altered autophagic pathway (Fig. 6h, i, Supplementary Fig. 6g). Although DOPAL promoted a comparable increase in size and fluorescence of p62 puncta for both wild-type and αSyn-null neurons, a significant decreased number of p62 puncta was measured upon DOPAL-treatment in the αSyn-null cells, while in wild-type neurons the p62 puncta density did not differ between untreated and DOPAL-treated neurons (Supplementary Fig. 6h). Thus, this led to a significant increase in the overall p62 levels only when both DOPAL and αSyn accumulate (Fig. 6j). Collectively, these data show that DOPAL neurotoxicity strongly depends on its interaction with αSyn, whose oligomerization leads to impaired neuronal proteostasis.

### Impaired DOPAL detoxification leads to αSynuclein accumulation, dopaminergic neuron loss and motor dysfunction

According to the evidence presented so far, DOPAL buildup affects αSyn and the whole cellular proteostasis, representing a putative key molecular mechanism of enhanced dopaminergic neuronal vulnerability. Therefore, we aimed at assessing this working hypothesis in both in vitro and in vivo models of endogenous DOPAL buildup through the impairment of aldehydes detoxification.

**Fig. 7:**
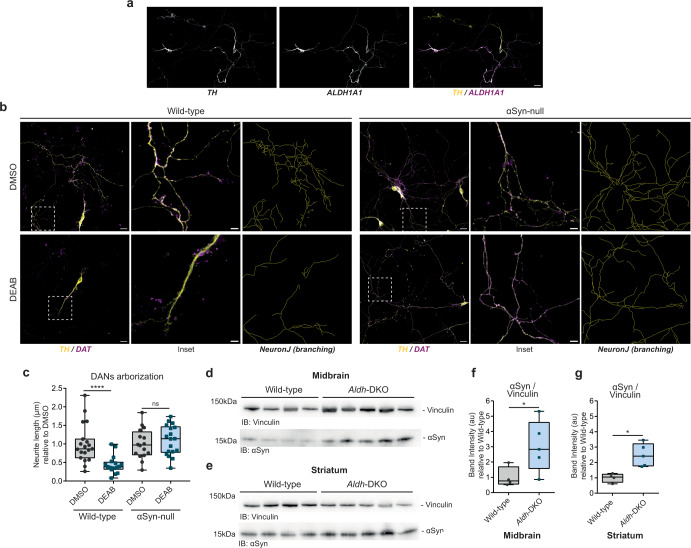
In vitro and in vivo mouse models of impaired DOPAL detoxification. **a** Immunostaining with dopaminergic markers in primary wild-type mouse mesencephalic neurons (DIV11), highlighting the co-localization between TH and ALDH1A1 in DANs. Scale bar: 20 μm. **b** Immunostaining with anti-TH and anti-DAT antibodies in primary mesencephalic neurons (DIV11) from wild-type and αSyn-null embryonic mice, after chronic exposure (4 days) to DMSO or 100 nM DEAB. Scale bar: 20 μm and 5 μm (in the inset). In the bottom row, the corresponding traces of neurite arborization obtained with NeuronJ. **c** Quantification of DANs total neurite length in each field of view. Data from two independent experiments are normalized to each DMSO-treated sample, pooled together, and analyzed by Mann–Whitney test (****p < 0.0001). Western blot of αSyn levels **d**, **f** in the midbrain and **e**, **g** in the *striatum* of 12-month-old wild-type (*n* = 4) and *Aldh*-DKO (*n* = 5) mice. Band intensities are normalized to Vinculin as loading control. Data are normalized to the mean value of the wild-type and analyzed by Mann–Whitney test (*p < 0.05). **c**, **f**, **g** Data are displayed as box and whiskers plot showing the minimum and maximum points (whiskers), the first quartile, median and third quartile (box lines).

We first obtained primary mesencephalic neuron preparations from embryonic wild-type mice, in which dopaminergic neurons (DANs) were identified as positive for the staining with the anti-TH antibody, also colocalizing with ALDH1A1 (Fig. 7a). Interestingly, when studying αSyn levels in neurons by immunofluorescence, we measured a significant increase of the protein in the soma of DANs as compared to non-DANs (Supplementary Fig. 7a, b), which was consistent with previous report^46^. Following a 4-day exposure to the ALDH inhibitor 4-diethylaminobenzaldehyde (DEAB, IC_50_ = 0.057 µM for hALDH1 and IC_50_ = 0.16 µM for hALDH2^47^) at the final concentration of 100 nM, we also observed a more pronounced accumulation of αSyn in the soma of DANs as compared to non-DANs (Supplementary Fig. 7c, d), suggesting the specificity of the impaired aldehyde catabolism in a dopaminergic system where αSyn accumulates as a consequence of DOPAL buildup. Moreover, we compared the extension of neurite branching in wild-type DANs versus DANs obtained from αSyn-null background mice (Fig. 7b). Of note, we measured a significant shrinking of the axonal arborization only in the wild-type neurons, consistent with our data highlighting the neurotoxic interplay between DOPAL and αSyn (Fig. 7c). Also, DEAB-treated wild-type DANs appeared to lose the co-localization between TH and DAT (Fig. 7b, in the insets), whereas DMSO-exposed wild-type and αSyn-null DANs, as well as DEAB-treated αSyn-null DANs, retained the expression of this other dopaminergic marker along their neurites.

We then aimed at testing our paradigm in in vivo models of endogenous DOPAL accumulation. Hence, we analyzed brain tissues from *Aldh1a1*^*−/−*^*/Aldh2*^*−/−*^ double knockout (*Aldh*-DKO) mice (Supplementary Fig. 7e, f), which were previously showed to display an age-dependent increased striatal DOPAL concentration, dopaminergic neurodegeneration, and parkinsonian-like phenotype when compared to wild-type littermates^48^. Here, we complemented this pathological picture with the analysis of αSyn levels, which resulted significantly increased in both the midbrain and *striatum* at 12 months-age (Fig. 7d–g).

**Fig. 8:**
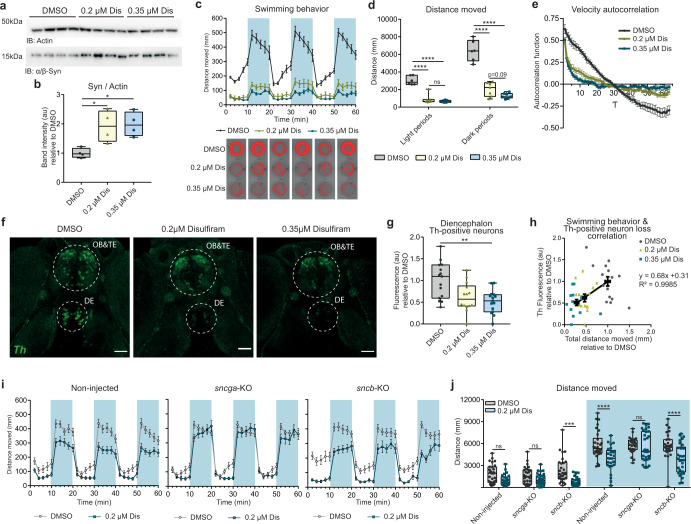
In vivo zebrafish model of impaired DOPAL detoxification. Biochemical, behavioral, and histological analysis in DMSO-, 0.2 µM Dis-, and 0.35 µM Dis-treated wild-type zebrafish larvae from 2 dpf to 5 dpf. **a** Immunoblot of Synuclein in lysates of zebrafish larvae heads, and **b** relative quantification. Data from four biological replicates are normalized to the mean value of the DMSO-treated samples and analyzed by Kruskall–Wallis test with Dunn’s multiple comparison test (*p < 0.05). **c** Time-course of the distance moved during the light-dark locomotion test acquired at DanioVision™. In the graph, the blue areas indicate the three dark stimuli of 10 min each. Data are presented as mean ± SEM from seven independent experiments. In the bottom part, representative tracks for each 10-min segment and each treatment are displayed. **d** Quantification of the total distance moved during the three light periods and the three dark periods. Data are analyzed by Two-way ANOVA with Sidak’s multiple comparison test (****p < 0.0001; interaction ****p < 0.0001, dark stimulus ****p < 0.0001, Disulfiram treatment ****p < 0.0001, subjects matching **p < 0.01). **e** Autocorrelation function of the time-course of velocity (10 second-bin) of movement. Data are presented as mean ± SEM from three independent experiments (DMSO: 15 larvae; 0.2 µM Dis: 14 larvae; 0.35 µM Dis: 14 larvae). **f** Immunostaining with the anti-TH antibody, which displays the dopaminergic neuron clusters OB&TE and DE. Scale bar: 50 µM. **g** Quantification of TH fluorescence signal in the dopaminergic neuron cluster in the DE. Data from three independent experiments pooled together and analyzed by Kruskall–Wallis non-parametric test with Dunn’s multiple comparison test (**p < 0.01). **h** Correlation of swimming behavior of single larvae with the relative TH-fluorescence intensity in the DE neuron cluster. Data are collected from three independent experiments (DMSO: 15 larvae; 0.2 µM Dis: 14 larvae; 0.35 µM Dis: 14 larvae), where both parameters are normalized to the mean value of the DMSO-treated sample, and the black dots represent the mean values (±SEM). **i** Time-course of the distance moved by DMSO- and 0.2 µM Dis-treated zebrafish larvae during the light-dark locomotion test. The three graphs correspond to the swimming behavior of non-injected larvae, and larvae injected with sgRNAs-Cas9 complexes to generate *sncga*-KO or *sncb*-KO F0. **j** Relative quantification of the total distance moved during the three light periods and the three dark periods (indicated by the light blue quadrant) of the swimming behavior analysis. Data from three independent experiments are analyzed by Two-way ANOVA with Sidak’s multiple comparison test (***p < 0.001, ****p < 0.0001). **b**, **d**, **g**, **j** Data are displayed as box and whiskers plot showing the minimum and maximum points (whiskers), the first quartile, median and third quartile (box lines). **c**, **e**, **i** Data are shown as mean ± SEM each time-points.

In a second in vivo model, we focused on the effect of the pharmacological inhibition of the Aldh enzyme in the brain of zebrafish larvae exposed to disulfiram (IC_50_ = 0.15 µM for hALDH1 and IC_50_ = 1.45 µM for hALDH2^49^), which hinders DOPAL detoxification and was reported to induce basal ganglia lesions after prolonged administration^50,51^. Here, zebrafish larvae were administered 0.2–0.35 µM disulfiram in water from 2 dpf to 6 dpf. Consistent with the data in the mouse tissues and in the mesencephalic neurons upon ALDHs inhibition, the disulfiram-exposed larvae displayed a significant accumulation of synuclein in the protein lysates extracted from the cephalic region (Fig. 8a, b). Although zebrafish lack of αSyn expression due to the putative loss of the ancestral *snca* locus during evolution^52^, both β-Synuclein (Sncb) and Ƴ1-Synuclein (Sncga) isoforms are expressed in zebrafish dopaminergic neurons and are known to regulate physiological striatal dopamine release and movement regulation^53^. In particular, β-Synuclein displays the highest sequence homology with human, mouse, and rat αSyn at the N-terminus (where the epitope of the antibody used in immunoblot – aa 2–25 – maps) (Supplementary Fig. 8a), whereas Ƴ1-Synuclein has been reported to be the functional homolog to αSyn^54^. Importantly, most of the lysine residues in the αSyn sequence are conserved in both zebrafish β-Synuclein and Ƴ1-Synuclein, therefore accessible to DOPAL modification.

As a second aspect, we assessed a disulfiram-induced impairment in the swimming behavior during the light-dark locomotion test^54^ (Fig. 8c). While the control larvae reacted to the dark stimulus by starting to swim faster in response to the sudden switching off of the light, the treated larvae were unable to behave likewise, and they covered a significantly reduced distance over the entire duration of the test, with the highest difference during the dark periods (Fig. 8d, Supplementary Fig. 7g, h). Moreover, the autocorrelation analysis of the velocity tracks during the light-dark locomotion test indicated that the disulfiram-exposed larvae had the tendency to display an intermittent swimming pattern compared to the DMSO-treated ones, suggesting a substantial impairment of the control of the voluntary movement (Fig. 8e, Supplementary Fig. 7i, Supplementary File 7). We then asked whether the resulting dysfunctional motor phenotype correlated with the loss of dopaminergic neurons of the diencephalon (DE), that is considered the nigral system of zebrafish^55,56^. The treatment with disulfiram resulted in the selective dose-dependent loss of the Th-positive neuron cluster in the DE (Fig. 8f, g), while the Th-positive neurons of the olfactory bulb and telencephalon (OB&TE) did not display a significant variation (Supplementary Fig. 7j). More importantly, we were able to correlate the quantification of the swimming phenotype of the zebrafish individual larvae with their level of Th in the DE neuron cluster, thus confirming the specific neurotoxic effect due to the Aldh inhibition in the dopaminergic diencephalon neurons (Fig. 8h).

Thereafter, we aimed at corroborating in vivo whether the lack of expression of Synuclein attenuates the neurotoxicity induced by an impaired DOPAL catabolism. To this end, we generated F0 zebrafish larvae lacking the expression of either *sncga* or *sncb*, exploiting a CRISPR-Cas9 KO approach previously described^57^. Briefly, zebrafish wild-type embryos at single-cell developmental stage were injected with gRNAs targeting each gene in three different exons in association to Cas9 protein (Supplementary File 1), which resulted in approximatively 50% and 80% decrease in *sncga* and *sncb* mRNA levels, respectively (Supplementary Fig. 8b, c). While the injection of gRNAs for *sncb* resulted in a non-significant decrease in *sncga* mRNA, the KO of *sncga* induced an increase in *sncb* expression levels, probably due to a compensatory effect in the whole brain tissue. Both control larvae (non-injected) and larvae injected with gRNAs for *sncga* or *sncb* where then exposed to either DMSO or 0.2 μM disulfiram from 2 dpf to 5 dpf, that we confirmed did not result in macroscopic morphological alterations (Supplementary Fig. 8d). We could not appreciate any variation in overall Synuclein protein levels by western blot, possibly due to the ability of the antibody we used to recognize both isoforms and the compensatory effect (Supplementary Fig. 8e). Nevertheless, we studied the swimming behavior during the light-dark locomotion test of non-injected, *sncga*-KO and *sncb*-KO larvae. Although the decreased expression of either *sncga* or *sncb* did not affect the motor phenotype in DMSO-exposed condition as compared to non-injected larvae, it emerged that the genetic ablation of the sole *sncga* gene completely prevented the impairment in motor performance and in the ability to respond to the dark stimulus upon disulfiram treatment (Fig. 8i, j, Supplementary Fig. 8f). According to the literature, while β-Synuclein is predominantly expressed in the OB&TE, Ƴ1-Synuclein has been reported to be predominantly expressed in the DE^53^, which corresponds to the region where we observed the selective loss of Th-positive neurons upon disulfiram exposure, thus indicating Ƴ1-Synuclein as the most relevant isoform for this paradigm. Overall, these data support in different in vitro and in vivo models that DOPAL buildup can lead to aberrant Synuclein accumulation, progressive dysfunction of the nigrostriatal pathway and severe motor phenotype.

### Detection of DOPAL-modified αSynuclein in human brains

Our data showed that DOPAL covalently modifies αSyn generating toxic oligomeric species which have great impact on neuronal physiology, leading dopaminergic neuron loss in vivo. Given the relevance of the proposed pathological mechanism for the preferential vulnerability of nigrostriatal neurons, we aimed at assessing its translational value in human brain from PD patients.

To address this issue, we developed a new rabbit monoclonal antibody designed to specifically detect the DOPAL modification on human αSyn by western blot. As epitope, we used synthetic peptides containing the αSyn repeat motif KTKEGV, where we introduced the covalent modification by DOPAC on a lysine residue by an amide bond (Supplementary Table 1). The rationale of this choice is that the amide bond between the carboxyl group of DOPAC and the amine group of lysine has a higher stability than the Schiff-base reaction generated by DOPAL. Furthermore, DOPAC has a high structural similarity with DOPAL allowing for an effective immunogenic response once injected in the rabbit. Several clones were obtained from the splenocytes isolated from the immunized rabbit and they were screened for their specific response against DOPAL-modified αSyn as compared to non-modified αSyn or non-specific targets (data not shown). Among all, the OBI-1-F1-10 clone supernatant was selected and tested by western blot against recombinant DOPAL-modified αSyn oligomers obtained in vitro and DOPAL-modified αSyn in 100 μM DOPAL-treated BE(2)-M17 cells overexpressing αSyn under a dox-inducible system (Supplementary Fig. 9a, b). Of note, in the DOPAL-treated cells, with equal amount of total αSyn, the signal detected by the OBI-1-F1-10 clone supernatant displayed some difference between the two samples, suggesting a certain degree of variability in the fraction of DOPAL-modified αSyn being a stochastic reaction. The then purified recombinant antibody was tested again by western blot against recombinant DOPAL-induced αSyn oligomers, presenting higher affinity towards the DOPAL-modified αSyn multimeric species as compared to the monomers (Supplementary Fig. 9c). Furthermore, we generated different αSyn oligomers obtained by the incubation with various catechols (L-DOPA, dopamine, DOPAL, DOPAC) and the lipid peroxidation product 4-hydroxynonenal (4-HNE), in a 1:15 αSyn:molecule ratio. Although all the molecules induced a rapid αSyn oligomerization on different levels as showed by the western blot with the MJFR1 antibody, the immunoblot with the purified OBI-1-F1-10 antibody recognized the DOPAL-induced αSyn oligomers with higher efficiency as compared to the other conditions (Supplementary Fig. 9d). Also, the comparison among the colors of the reactions suggested different degrees of catechol oxidation (yellow/brown shades as read out), with L-DOPA and dopamine reactions resulting in the highest level of oxidation and possibly polymerization in neuromelanin-like molecules (Supplementary Fig. 9d). DOPAL, instead, displayed a lower degree of oxidation, which might also indicate that the molecule covalently modified αSyn lysines, as validated by the nIRF detection, rather than undergo self-polymerization (Supplementary Fig. 9d).

**Fig. 9:**
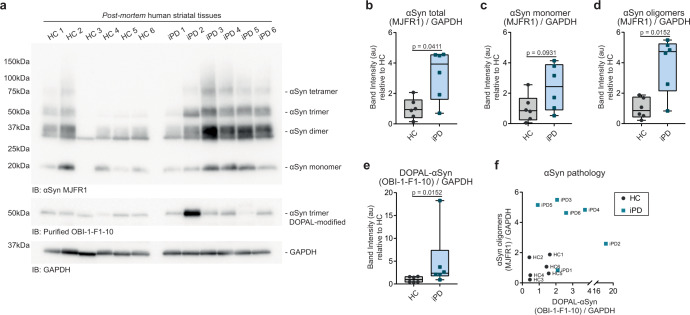
Detection of DOPAL-modified αSynuclein in human post-mortem striatal tissues. **a** Western blot of human post-mortem striatal tissues from six idiopathic PD patients (iPD1-6) and six age-matched healthy controls (HC1-6). The MJFR1 antibody detected monomeric and high-molecular weight oligomeric αSyn species, while the purified recombinant OBI-1-F1-10 RabMab antibody developed in this study detected DOPAL-modified αSyn trimers. GAPDH was used as loading control. Relative quantification of **b** total αSyn, **c** monomeric αSyn, **d** oligomeric αSyn, and **e** DOPAL-modified αSyn. Data are normalized to the mean value of healthy controls and analyzed by Mann–Whitney test. Data are displayed as box and whiskers plot showing the minimum and maximum points (whiskers), the first quartile, median and third quartile (box lines). **f** Correlation of the levels of DOPAL-modified αSyn (x-axis) and oligomeric αSyn (y-axis) in each sample.

Finally, we used the purified recombinant OBI-1-F1-10 antibody in lysates of human *post-mortem* striatal tissues isolated from the brains of six idiopathic PD patients (iPD) and six age-matched healthy controls (HC) (Supplementary Table 2). The western blot analysis using the MJFR1 antibody by Abcam revealed significantly elevated αSyn pathology in the iPD samples, mostly due to the presence of higher levels of oligomeric αSyn (Fig. 9a–d). When we incubated the membrane with the OBI-1-F1-10 antibody, we detected a single band at 50 kDa molecular weight which putatively corresponds to DOPAL-modified αSyn trimers, suggesting this αSyn form might be the more stable and therefore more likely to be detected by the OBI-1-F1-10 in brain-derived tissue lysates (Supplementary Fig. 9e, Fig. 9a). Importantly, the levels of DOPAL-modified αSyn were significantly higher in the iPD samples as compared to HC samples, with a positive correlation with the amount of αSyn oligomers detected by the MJFR1 antibody (Fig. 9e, f). Even though some healthy controls presented relatively high levels of αSyn pathology, which we speculated to be due to aging or to other unspecified conditions, five out of six iPD samples reported systematic increased levels of DOPAL-modified αSyn, suggesting consistent dyshomeostasis of the dopaminergic pathway which indeed affected αSyn proteostasis in those patients.

## Discussion

In this study, we provided evidence on the impact of DOPAL-induced αSyn oligomerization on neuronal proteostasis, showing that DOPAL endotoxicity strongly depends on its interaction with αSyn and is exacerbated at synapses and neuronal projections. This eventually results in a reduced axonal arborization and dopaminergic neuron loss, recapitulating the main pathological hallmarks of PD.

The notion that dopamine and αSyn can synergistically act to cause degeneration of *SNpc* neurons has been reported and substantiated in animal models of altered dopamine metabolic pathways^10,23,58^. Increasing levels of oxidized dopamine were demonstrated to generate αSyn spherical oligomers that are not able to evolve into fibrils^59,60^. However, the dopamine-induced αSyn aggregates are non-covalent adducts, mainly driven by conformational changes in the αSyn C-terminus^10,60–62^. Although in vitro incubation of recombinant αSyn generates oligomeric species, the precise mechanism of dopamine modification of αSyn in vivo has not been defined yet. Here, the aggregation assay presented in Fig. 1a demonstrated a faster kinetics for DOPAL-dependent αSyn oligomerization (less than 24 h) as compared to dopamine incubated in the same molar ratio (up to 6 days^63^). Even though, in 24 h, dopamine could generate a small fraction of αSyn oligomers detectable by western blot, it is likely that the number of dopamine molecules interacting with the protein is still below the detection limit for the instrument used to measure the nIRF signal, in contrast to the high degree of DOPAL-covalent modification on αSyn lysines.

Although the ability of DOPAL to covalently modify αSyn and trigger its oligomerization has been previously demonstrated^23–26^, the unraveling of a specific DOPAL effect on aSyn aggregation, localization, and clearance among neuronal compartments is missing. As we observed a significant increase in αSyn levels in all neuronal districts, the DOPAL-induced alterations in the live-cell time-lapse imaging experiments potentially derived from a combination of an impaired mobility of αSyn along the neurites and a decreased degradation efficiency both in the periphery and the soma, where different pathways are involved. At synaptic level, αSyn monomers and small *on-pathway* oligomers are known to be degraded by the UPS and local proteases, whereas in the cell body the autophagic pathway participates as additional backup, especially for the clearance of larger aggregates^64^. Interestingly, this might be reflected by the αSyn-YFP-miniSOG half-lives that we estimated in the two different regions by live imaging in the presence of Nocodazole, where the αSyn-YFP-miniSOG turnover was about 2.5 times longer in the periphery as compared to the soma. Although we determined a t½ that differs from those observed in previous research reports^45,65^, this may be ascribed to the specific cellular model and/or αSyn probe and pulse-chase strategy used. On the same line, we measured a much longer αSyn turn-over in αSyn-HaloTag expressing BE(2)-M17, however, the absolute values of αSyn half-life, being contest-dependent, may have a limited significance. Nevertheless, its variation among different subcellular compartments in primary neurons, likely related to the local machinery for protein quality control, may be of relevance for untangling protein homeostasis in physio-pathological conditions.

DOPAL-induced αSyn oligomers have been defined *off-pathway*, as they do not acquire a unique structural conformation nor undergo fibrillation^24^. Moreover, the DOPAL modification on αSyn lysines is likely to interfere with the UPS-operated degradation. Hence, we speculated that the synaptic protein quality control machineries might not be fast enough to rapidly dispose the buildup of DOPAL-αSyn toxic species, thus requiring the engagement of the endosomal pathway to redirect these aggregates to the autophagic clearance in the soma. In support of this mechanism, in DOPAL-treated neurons, we observed an overload of oligomeric αSyn in MVBs, which are retrogradely transported to the soma where they fuse with the lysosomes to degrade their content. Interestingly, it has been recently demonstrated that neurons can deliver active lysosomes to distal axons to contribute to αSyn degradation from synapses, further supporting our hypothesis of the engagement of the autophagy-lysosomal pathway in the clearance of aberrant αSyn species in the neuronal projections^66^. Consistently, we observed a massive deposition of αSyn aggregates in lysosomal-like compartments in DOPAL-treated primary neurons and BE(2)-M17 cells by CLEM, suggesting a challenged autophagic pathway. Also, the K96R mutation, which has been found as a benign variation in a small fraction of the population with a global minor allele frequency of 0.0016 (source: gnomad.broadinstitute.org), displayed a decreased loading of αSyn along the endo-lysosomal pathway in DOPAL-treated cells, thus significantly affecting protein clearance. Not only lysine 96 is required for αSyn ubiquitination by Nedd4 for the endosomal degradation^45^, but it is within the recognition motif for chaperone-mediated autophagic (CMA) degradation of αSyn^67^. Both dopamine-induced αSyn oligomers and phosphorylated αSyn at Ser129 were showed to block the LAMP2A receptor, thus affecting CMA activity towards other substrates and contributing to neuronal protein dyshomeostasis^67^. In this frame, DOPAL-modified αSyn, which we also found to be more phosphorylated, could contribute to CMA impairment. Of note, the clogging of lysosomes and disruption of axonal network are reported to promote the fusion of the MVBs with the plasma membrane, releasing their vesicles in the extracellular space^42,68^, consistent with our observations in αSyn-overexpressing DOPAL-treated cells. This is extremely relevant as exosomes represent a spreading pathway of αSyn toxic oligomers to neighbor neurons and glial cells^43^, thus augmenting both neurodegeneration and neuroinflammation.

We further revealed that DOPAL buildup triggers αSyn-mediated neurotoxicity, compromising neuronal resilience. Interestingly, the greatest effects were observed at the neuronal distal regions and at the synaptic level, where αSyn concentration is higher. Here, αSyn reasonably represents a preferential target of DOPAL reactivity, as 10.7% of αSyn sequence is composed of lysine residues, a percentage which is higher than the average value of the lysine fraction in synaptic proteins (around 5%)^69^. At synapses, the DOPAL-induced αSyn accumulation appeared to affect synaptic vesicle organization and clustering, and we previously demonstrated that DOPAL-buildup induced a redistribution of synaptic vesicles from the ready-releasable pool to the resting pool^24^. Taken together, these observations suggest that DOPAL and αSyn pathological interplay can lead to synaptic dysfunction, eventually reducing synapse density as reported by the VAMP2- and Bassoon-positive puncta quantification. It has been recently corroborated that αSyn synaptic pathology causes alterations in the firing activity of midbrain dopaminergic neurons and dopamine release at the striatum with a time dependent progression, recapitulating a distant dopaminergic degeneration and the dying-back paradigm in the early stages of PD^70^. Future studies will address functional alterations of synaptic activity induced by αSyn-DOPAL interplay.

The distal regions in neurons are also more vulnerable to the accumulation of misfolded and aggregated proteins, as they are less equipped with machineries for protein quality control and homeostasis^5^. A consistent pool of ubiquitin monomers is constantly available in the neuronal periphery, to ensure a rapid response in stress conditions and mediate the degradation of aberrant proteins by both the proteasome and the selective autophagy^71,72^. Of note, we detected increased ubiquitin levels in the neuronal projections only in DOPAL-treated wild-type neurons, correlating with a higher density of LAMP1-positive structures in the distal portion of neurites and the overload of αSyn-positive MVBs. Both the synaptic injury and the overwhelmed endolysosomal pathway were observed to be dependent on αSyn expression, considering that the DOPAL-associated effects were limited in neurons with the αSyn-null background, thus corroborating the unique toxic interplay between DOPAL and αSyn.

On another note, it has to be considered that DOPAL presents a broad spectrum of reactivity and an impaired proteostasis induced by DOPAL modification would be more obvious on proteins with fast turn-over and prone to aggregation, as we observed for p62. In this case, a combination of DOPAL direct modification of p62 on lysines and cysteines (via the aldehyde and catechol moieties, respectively), p62 increased expression induced by antioxidant responses, and a blocked autophagic flux might all contribute to p62 accumulation and oligomerization^73^, which need further investigations. At the same time, we cannot completely exclude an indirect effect on αSyn accumulation and aggregation due to DOPAL modification of different targets, possibly in an oxidation-dependent manner, which might influence αSyn proteostasis itself. However, to single out this mechanism a mutant form of αSyn lacking the DOPAL modification sites should be used. However, the substitution of the many lysine residues on αSyn sequence would completely alter the nature and the physiological properties of the protein itself.

As aggregated αSyn, highly phosphorylated at Ser129, ubiquitin, p62, and lipid membranes are the main constituents of Lewy Bodies (LBs)^74–76^, these observations become of interest in the context of PD pathology. It may be speculated that, as αSyn-DOPAL oligomers burden impedes degradative pathways and neuronal proteostasis in general, this might lead to αSyn accumulation with enhanced *on-pathway* fibrillation in the cell body, further contributing to LBs formation.

A consideration is due on our in vitro models, as we mainly used non-dopaminergic neuronal cultures that did not allow to induce endogenous DOPAL buildup. This choice has its rationale in circumventing experimental limits and the impaired dopamine catabolism in PD. First, the in vitro paradigm aimed at modeling in a short time span the neurotoxic outcomes that would result from a chronic accumulation of endogenous DOPAL in a dopaminergic system. A substantial body of evidence demonstrated a decreased expression and activity of ALDH1A1 in PD patients’ nigrostriatal neurons, highly affecting dopamine catabolism and increasing DOPAL levels^14^. Hence, the non-dopaminergic neurons fairly represent a similar scenario as they only express the mitochondrial ubiquitous ALDH2 which has lower affinity for DOPAL as compared to ALDH1A1^77^. Here, we applied DOPAL treatments to the cell culture medium using a concentration that would ensure a significant increase in the amount of intracellular DOPAL, as assessed by nIRF detection of DOPAL-modified proteins in the cell lysate. Also, we previously demonstrated that, following overnight administration (at a concentration of 100 μM), DOPAL can in fact interact with αSyn in cells and covalently modify its lysines, as detected by mass spectrometry^24^. At the same time, it is very difficult to obtain pure dopaminergic primary neuronal cultures in which dopamine catabolic pathway can be modulated and it may compromise the possibility to detect an effect. Nevertheless, we managed to obtain primary mesencephalic dopaminergic neurons in which we could demonstrate that an impaired DOPAL detoxification by ALDH inhibition dramatically affects axonal arborization in an αSyn-dependent mode, overall supporting a contributing mechanism for the dying-back hypothesis of dopaminergic neurodegeneration. Furthermore, we observed a reduced co-localization between TH and DAT only in the DEAB-exposed wild-type neurons, which we speculated to be an indication of synapse dysfunction accompanied by the axonal degeneration. Of note, αSyn aggregates have been previously demonstrated to interact with DAT affecting its levels and trafficking at the striatal terminals^78,79^. Hence, it will be of importance to untangle whether DOPAL-αSyn oligomers can interfere with DAT localization and function in the early synaptic degeneration.

Finally, to fully validate the relevance of our in vitro results, we tested our working hypothesis in two independent in vivo models of defective endogenous DOPAL degradation, as a molecular mechanism to enhance dopaminergic susceptibility in PD. Both aged *Aldh*-DKO mice, previously shown to display dopaminergic neurodegeneration and motor impairment^48^, and zebrafish larvae exposed to disulfiram, a potent ALDHs inhibitor, presented a significant accumulation of Synuclein in the brain tissues. Disulfiram-exposed zebrafish larvae recapitulated a parkinsonian motor phenotype, displaying decreased distance traveled during the light-dark locomotion test and a discontinuous pattern of movement. We also demonstrated that the impaired swimming behavior strongly correlated with the selective loss of Th-positive neurons in the diencephalon. More importantly, the specific knock-out for Ƴ1-Synuclein, the relevant isoform for the Th-positive neurons of the DE in zebrafish, completely prevented the disulfiram-induced motor phenotype, once again corroborating the neurotoxic αSyn-DOPAL interplay. Although our analysis covered some of the aspects of DOPAL neurotoxicity in vivo, our evidence emerged as proof-of-concept of the impaired αSyn proteostasis when DOPAL detoxification is hampered, which reveals to be detrimental for the highly vulnerable dopaminergic neurons.

The mechanism proposed in this study acquires significance if one considers its specificity for dopaminergic nigrostriatal neurons, at the early stages of PD neurodegeneration. The relevance of our results also hinge on epidemiological studies that correlated an increased risk of developing PD with the exposure to drugs, pesticides, and chemicals that act as ALDHs inhibitors^14,55^, enhanced by some genetic variations on *Aldh1a1* and *Aldh2* genes that were recently identified^14,80,81^. Unfortunately, only a few studies investigated the presence of DOPAL-modified αSyn species in PD autoptic samples, mostly because of the lack of reliable tools for their detection. Only one recent work reported increased levels of “αSyn dopaminylation” in the plasma of PD patients^82^. Here, we put great efforts in the development of a specific monoclonal antibody for the detection of DOPAL-modified αSyn in human *post-mortem* brain samples, which we used to demonstrate significantly higher levels of DOPAL-modified αSyn species in the striatal regions of idiopathic PD patients as compared to healthy controls, correlating with αSyn pathology. Hence, this tool will represent, when validated in larger cohorts, a key step forward for improving the histopathological characterization of PD patients and, eventually, the development of new diagnostic strategies.

We believe that the identification of specific pathological pathways would contribute to the definition of effective translational approaches for early clinical interventions in PD patients. In this frame, the implementation of genetic screenings and suitable biomarkers oriented to the identification of subjects with increased risk of pathological DOPAL accumulation would be crucial for the early diagnosis of PD^83^. Such novel patient stratification strategies would also lead to a reconsideration of MAO inhibitors as therapeutic approach which, in past studies, did not match the requisites of disease-modifiers for PD.

## Methods

### DOPAL synthesis

DOPAL was produced following the method by Fellman, with slight modification. Briefly, 6 ml 85% ortho-phosphoric acid preheated to 120 °C in an oil bath were added to 400 mg of (±)-epinephrine hydrochloride (E4642, Sigma-Aldrich) in a 2-dram glass scintillation vial. The epinephrine-acid mixture was then vortexed until dissolved, re-submerged in the oil bath until it changed from yellow to a dark orange color and then added to 60 ml of H_2_O in a separating funnel. The water mixture was then extracted with 20 ml of ethyl acetate twice, washed with 10 ml of H_2_O and the combined organic layers were evaporated in a Savant SpeedVac concentrator until a constant mass was obtained. The resulting gel was first resuspended in 100% methanol, followed by evaporation by an N_2_ stream. DOPAL was then resuspended in distilled H_2_O and concentration was determined by assuming the same extinction molar coefficient of dopamine and L-DOPA (ε_280nm_ = 2.63 cm^−1^ mM^−1^). The quality of the DOPAL preparation was analyzed by reverse-phase high pressure liquid chromatography (RP-HPLC) on a Phenomenex Jupiter column (300 Å/5 μm, 250 mm × 4.6 mm), using linear gradients of solvent B in eluent A (A: 0.1% trifluoroacetic acid (TFA) in H_2_O, B: 0.08% TFA in acetonitrile; gradient: 5% B to 65% B in 20 min) and purity was calculated to be about 95%, based on the absorbance measured at 280 nm (Supplementary Fig. 1a). DOPAL unique retention time and profile in RP-HPLC was compared to other catechols (DOPET, DOPAC, epinephrine, norepinephrine, dopamine, L-DOPA, homovanillic acid), confirming the efficacy of the synthesis protocol in converting the epinephrine into DOPAL and in removing the reaction sub-products (Supplementary Fig. 1b). Consistently, both the absorbance spectrum and the RP-HPLC profile of the synthetized DOPAL perfectly overlap with DOPAL purchased from Santa Cruz Biotechnology (SCBT; sc-391117) that was used as a reference (Supplementary Fig. 1c, d). Stocks of 100 mM DOPAL in H_2_O were stored at −80 °C until used.

### Recombinant αSynuclein purification

Recombinant human αSyn was purified as previously described^24^. Briefly, the αSyn gene was cloned in pET-28a plasmid (Novagen) and expressed in *Escherichia coli* BL21(DE3) strain. Bacteria were grown to an OD_600nm_ of 0.3–0.4 and induced with 0.1 mM isopropyl b-D-1-thiogalactopyranoside (IPTG). After 5 h, cells were collected by centrifugation and recombinant proteins recovered from the periplasm by osmotic shock. Subsequently, the periplasmic homogenate was boiled for 15 min and the soluble αSyn-containing fraction was subjected to a two-step (35 and 55%) ammonium sulfate precipitation. The pellet was then resuspended, extensively dialyzed against 20 mM Tris–HCl, pH 8.0, loaded into a 6 ml Resource Q column (Amersham Biosciences), and eluted with a 0–500 mM gradient of NaCl. Proteins were then dialyzed against water, lyophilized, and stored at −20 °C.

### In vitro DOPAL-induced αSynuclein oligomerization

Recombinant αSyn was resuspended in phosphate-buffered saline (PBS) and concentration was determined by measuring the absorbance at 276 nm and using the ε_276nm_ = 5.8 cm^−1^ mM^−1^. αSyn oligomers were produced by incubating recombinant αSyn to a final concentration of 20 µM with 300 µM DOPAL or dopamine (Sigma-Aldrich) in PBS, in a ratio 1:15 αSyn:catechol which corresponds to 1:1 lysine:catechol. The solutions were incubated for 0-2-4-8-16-24-48 h shaking at 350 rpm at 37 °C. At each time-point, 30 µl of the reactions (10 µg of αSyn) were collected and the reaction was stopped by the addition of loading buffer. Monomeric αSyn and αSyn oligomers were then resolved by SDS-PAGE into a gradient 4–20% SDS-Page gel (GenScript) and transferred to polyvinylidenedifluoride (PVDF) membranes (BioRad), through a semi-dry Trans-Blot® Turbo™ Transfer System (BioRad). Membrane was scanned at the LiCor Odissey for the near Infra-Red Fluorescence (nIRF) detection at 800 nm, followed by the immunoblot with the rabbit anti-αSyn MJFR1 (ab138501, Abcam) antibody. For the limited proteolysis assay with Proteinase K (PK), DOPAL-αSyn oligomers were produced by overnight incubation of 20 µM αSyn with 600 µM DOPAL in PBS. Monomeric αSyn and DOPAL-αSyn oligomers were then incubated with 50 nM PK (Invitrogen) in PBS for 10-20-30 min, and reactions were stopped by the addition of loading buffer. Proteins were then separated by SDS-Page, followed by staining with Coomassie brilliant blue (0.15% Coomassie Brilliant Blue R, 40% ethanol), followed by destaining with 10% isopropanol, 10% acetic acid. Image analysis was then performed with Fiji software.

### Plasmids for mammalian cell expression

The following constructs for primary neuronal cultures and cell line transfection were generated at NCMIR (UCSD): pCAGGS.αSyn-miniSOG full-length; pCAGGS.αSyn-TimeSTAMP-YFP-miniSOG; pCAGGS.αSyn-miniSOG_1-94_(Fragment A) and pCAGGS.αSyn-miniSOG-Jα_95-140_(Fragment B). The pEGFP-N1.αSyn and the pHT2.αSyn-HaloTag for cell line transfection were generated in Prof. Bubacco’s lab (UNIPD) by cloning the αSyn-encoding sequence into the pEGFP-N1 empty vector (Novagen) and the pHT2 vector, respectively, previously described^24,40^. The αSyn K96R variant was generated using the Quick-Change II site-directed mutagenesis kit (Stratagene) according to manufacturer’s instructions, using the following primers: FOR: 5’ ATTGGCTTTGTCAGAAAGGACCAGTTGG 3’; REV: 5’ CCAACTGGTCCTTTCTGACAAAGCCAGTG 3’. αSyn(WT)-HaloTag and αSyn(K96R)-HaloTag cDNA were then cloned in the pLKO.DEST.hygro (Addgene, #32685) lentiviral vector, and αSyn cDNA in the pInducer20 lentiviral vector (Addgene, #44012^84^) using Gateway Recombination Cloning Technology (Thermo Fisher), generating the pLKO.αSyn(WT)-HaloTag.hygro, the pLKO.αSyn(K96R)-HaloTag.hygro and the pInd.αSyn lentiviral constructs, respectively. The pEGFP-CD63 (#62964), pCMV-VSV-G (#8454) and pCMV-dR8.2 (#8455) plasmids were purchased at Addgene.

### Primary mouse cortical neuron preparation and treatments

C57BL/6LOlaHsd and C57BL/6JRccHsd mice, αSyn-null and wild-type respectively, were purchased from Envigo Srl. Mouse genotyping was performed with the Phire™ Tissue Direct PCR Master mix (F-170S, ThermoFisher) using primers previously described (mouse Snca exon 6: AAGACTATGAGCCTGAAGCCTAAG and AGTGTGAAGCCACAACAATATCC, 266 bp fragment; deletion junction, termed D6Slab17: TTGATAGTTCCACTGTTCTGGC and GTAACAATACAGCAAGAGATAC, 179 bp fragment)^85^. Animals were maintained and experiments were conducted according to the Italian Ministry of Health and the approval by the Ethical Committee of the University of Padova (Protocol Permit #200/2019-PR). Cortical neurons were dissociated by papain from αSyn-null and wild-type mice postnatal day 0-1 (P0-1) pups as previously reported^24^ and cultured in Neurobasal A medium (Life Technologies) supplemented with 2% v/v B27 Supplements (Life Technologies), 0.5 mM L-glutamine (Life Technologies), 100 U/mL penicillin, and 100 µg/mL streptomycin (Life Technologies) for 12 days prior to imaging and western blot analysis, refreshing half medium every 3 days. Treatments with 100 µM DOPAL were performed in complete medium for 24 h.

### Primary embryonic mouse ventral mesencephalic neurons

Primary embryonic mouse ventral mesencephalic cells were isolated from C57BL/6J (wild-type) and C57BL/6JOlaHasd (αSyn-null) mice at the animal house facility at the Department of Molecular and Translational Medicine of University of Brescia (Italy). All experiments were made in accordance with Directive 2010/63/EU of the European Parliament and of the Council of 22 September 2010 on the protection of animals used. All experimental procedures were conformed to the National Research Guide for the Care and Use of Laboratory Animals and were approved by the Animal Research Committees of the University of Brescia (Protocol Permit 719/2015-PR). Briefly, ventral mesencephalic tissues were dissected from wild-type and αSyn-null mice at embryonic day 13.5 (E13.5) as previously reported^86^, and cultured onto poly-D-lysine-coated glass coverslips in 24-wells plates in Neurobasal medium (Gibco) containing 100 μg/ml penicillin, 100 μg/ml streptomycin (Sigma-Aldrich), 2 mM glutamine (EuroClone) and 1% B27 supplement (Gibco). Treatments with DMSO (Sigma-Aldrich) or 100 nM 4-diethylaminobenzaldehyde (DEAB, D86256, Sigma-Aldrich) dissolved in DMSO, were performed from DIV7 to DIV11, refreshing half medium every day. At the end of chronic exposure to ALDH inhibitor, cells were fixed and analyzed by immunofluorescence using the anti-TH antibody to identify dopaminergic neurons (DANs).

### Primary rat cortical neuron preparation, transfection, and treatments

Cortical neurons were dissociated by papain from P2 Sprague-Dawley rats as previously reported^36^ and according to the animal procedures approved by the Institutional Animal Care and Use Committee of UC San Diego. Neurons were transfected by electroporation using an Amaxa Nucleofection Device (Lonza) at day-in-vitro 0 (DIV0) and cultured in Neurobasal A medium (Life Technologies) supplemented with 1X B27 Supplements (Life Technologies), 2 mM GlutaMAX (Life Technologies), 20 U/mL penicillin, and 50 mg/mL streptomycin (Life Technologies) for 10–15 days prior to imaging, refreshing half medium every couple of days. In the αSyn-split-miniSOG experiments, neurons were co-transduced with lentiviral vectors HIV1 expressing αSyn-miniSOG_1-94_(Fragment A) and αSyn-miniSOG-Jα_95-140_(Fragment B) at DIV 7. Treatments with 100 µM DOPAL, 1 µM BILN-2061 (ACME Synthetic Chemical) and 5 µg/ml Nocodazole (487928, Millipore) were performed in complete medium for the time frame specified in each experiment.

### BE(2)-M17 cell line maintenance, transfection, and treatments

Neuroblastoma-derived BE(2)-M17 cells (ATCC CRL-2267) were cultured in 50% of Dulbecco’s modified Eagle’s medium (DMEM, Life Technologies) and 50% of F-12 Nutrient Mix (Life Technologies), supplemented with 10% v/v fetal bovine serum (FBS) and 1% Penicillin/Streptomycin (Life technologies). Before transfection and treatments, cells were maintained in complete medium supplemented with 10 µM retinoic acid (RA, Sigma-Aldrich) for three days. When at 80% of confluency, cells were transiently transfected using Lipofectamine 2000 (Invitrogen) with a DNA(µg):Lipofectamine(µl) ratio of 1:2. Cells were then treated and processed 24-to-72 h post transfection. DOPAL treatments were performed at the final concentration of 100 µM in OptiMEM, unless indicated otherwise. 100 µM DOPAL and 10 mM aminoguanidine hydrochloride (AMG, 396494, Sigma-Aldrich) co-treatments, as well as treatments with 50 μM cycloheximide (CHX, Sc-3508, SCBT), 20 μM MG132 (Sc-201270, SCBT), 50 μM chloroquine (C6628, Sigma-Aldrich) were performed in OptiMEM overnight (unless indicated otherwise). Starvation was induced by an overnight incubation with serum-free medium.

### Generation of inducible stable BE(2)-M17-αSyn cell line

HEK293-FT cells, used for lentivirus production, were cultured in DMEM (Voden) supplemented with 10% FBS (Corning) and 1% Penicillin/Streptomycin (Life technologies). Cells were co-transfected with pdR8.2, pVSV-G, and pInd.αSyn or pLKO.αSyn(WT)-HaloTag.hygro and pLKO.αSyn(K96R)-HaloTag.hygro constructs (2:1:4) using Polyethylenimine (23966-1, Polysciences Inc) a DNA(µg):Lipofectamine(µl) ratio of 1:4. 72 h post transfection, cell medium was collected and centrifuged for 10 min at 1000 × *g* at 4 °C to remove cell debris. After filtration with 0.45 µm sterile filters (Sarstedt), the lentivirus isolation was obtained by ultracentrifugation at 50,000 × *g* for 2 h at 4 °C. Lentiviral pellet was then resuspended in PBS supplemented with 5% bovine serum albumin (BSA) and stored at −80 °C until used. BE(2)-M17 at 40% confluency were transduced with the lentiviral particles and infected cells were positively selected by the addition of 500 µg/ml G418 (InvivoGen, for pInd.αSyn lentivirus) or 250 μg/ml hygromycing (InvivoGen, for pLKO.αSyn-HaloTag.hygro) in the cell medium for 48 h, thus generating a polyclonal stable BE(2)-M17 cell lines. In the inducible BE(2)-M17-αSyn cell line, αSyn overexpression was induced by the addition of 100 ng/ml doxycycline (dox) in the cell medium for 48 h, while the non-induced cells were used as control (0 ng/ml dox).

### Catechols detection by nIRF in cell lysate

Catechol-modification of proteins was assessed in DOPAL-treated cells as described in Mazzulli et al. with slight modification^29^. Untreated BE(2)-M17 cells and DOPAL-treated cells were harvested in radioimmunoprecipitation assay (RIPA) buffer (Cell Signaling Technology) supplemented with protease inhibitors cocktail (Roche). After lysates centrifugation at 20,000 × *g* at 4 °C, the cleared supernatant was collected and the protein content in the soluble fraction was determined using the Pierce® BCA Protein Assay Kit (Thermo Scientific) following the manufacturer’s instructions. The insoluble fraction in the pellet of the cell lysate was resuspended in 1 M NaOH (volumes were adjusted according to the protein quantification in the soluble fraction), followed by incubation at 37 °C for 30 min, sonication at room temperature for 20 min (bath sonicator, 100% power and 80 Hz frequency) and centrifugation at maximum speed at room temperature for 20 min. The pellets were then dried at 95 °C and resuspended in 10 µl of water. Both the soluble (40 µg of proteins) and the insoluble (4 µl) fractions were spotted on nitrocellulose membrane (BioRad). The near infrared fluorescence (nIRF) signal was acquired by scanning the membrane at the LiCor Odissey using the 800 nm filter and images were analyzed using the Fiji software.

### Western blot

Primary mouse cortical neurons and BE(2)-M17 cells were harvested in RIPA buffer (Cell Signaling Technology) supplemented with protease inhibitors cocktail (Roche). Lysates were clarified by centrifugation at 20,000 × *g* at 4 °C. Protein concentration in the cleared supernatant was determined using the Pierce® BCA Protein Assay Kit (Thermo Scientific) following the manufacturer’s instructions and protein samples were loaded on gradient 4–20% Tris-MOPS-SDS gels (GenScript). The resolved proteins were then transferred to PVDF membranes (BioRad), through a semi-dry Trans-Blot® Turbo™ Transfer System (BioRad). PVDF membranes were subsequently blocked in Tris-buffered saline plus 0.1% Tween (TBS-T) and 5% non-fat dry milk for 1 h at 4 °C and then incubated over-night at 4 °C with primary antibodies diluted in TBS-T plus 5% non-fat milk. The following primary antibodies were used: mouse anti-α-Tubulin (T6074, Sigma-Aldrich), mouse anti-αSyn 211 (S5566, Sigma-Aldrich), mouse anti-αSyn pSer129 81/A (825702, BioLegend), mouse anti-β-Tubulin III (T8578, Sigma-Aldrich), mouse anti-Syn-1 (610787, BD Transduction Laboratories), rabbit anti-αSyn pSer129 EP1536Y (ab51253, Abcam), rabbit anti-αSyn MJFR1 (ab138501, Abcam), mouse anti-β-Actin (A1978, Sigma-Aldrich), rabbit anti-Alix (ABC40, Millipore), mouse anti-HSP90 (SPA830, ENZO Life Sciences), mouse anti-HSP70 (SPA810, ENZO Life Sciences), mouse anti-Flotillin-1 (610822, BD Transduction Laboratories), rabbit anti-CD9 (ab92726, Abcam), rabbit anti-Vinculin (AB6039, Millipore), rabbit anti-ALDH1A1 (GTX123973, GeneTex), rabbit anti-ALDH2 (GTX101429, GeneTex), rabbit anti-α/β.Synuclein (1280 002, SYSY), mouse anti-GAPDH (CSB-MA000195, Cusabio). After incubation with hose-radish peroxidase (HRP)-conjugated secondary antibodies (goat anti-rabbit-HRP and goat anti-mouse-HRP, Sigma-Aldrich) at room temperature for 1 h, immunoreactive proteins were visualized using Immobilon® Classico Western HRP Substrate (Millipore) or Immobilon® Forte Western HRP Substrate (Millipore) by Imager CHEMI Premium detector (VWR). The densiometric analysis of the detected bands was performed by using the Fiji software. All blots or gels derive from the same experiment and that they were processed in parallel.

### ELISA assay on neuron lysates

Quantification of αSyn aggregates in the neuron lysates was performed by custom-made enzyme-linked immunosorbent assay (ELISA) as described by Lassen et al.^87^ with slight modifications. Briefly, F96 MaxiSorp Nunc-Immuno Plate (Thermo Scientific) was coated overnight at 4 °C with 100 µl/well of 62.5 ng/ml rabbit anti-aggregated αSyn MJFR14-6-4-2 (ab209538, Abcam), used as capturing antibody, diluted in 0.1 M NaHCO_3_ in PBS pH 9.5. The following day, the plate was washed four times in TBS – 0.05% Tween (TBS-T) and saturated in 1X ELISPOT blocking solution (00-4202-56, Invitrogen) for 3 h at room temperature. After four washes in TBS-T, 20 µg of proteins diluted in 100 µl of 1X ELISPOT were incubated in each well overnight at 4 °C. Seven paired untreated and DOPAL-treated neuron samples were measured in triplicate and lysate from primary astrocytes was used as negative control of absence of αSyn. Increasing amounts (1-10-100-1000 pg) of recombinant monomeric αSyn and αSyn-DOPAL oligomers were included as positive controls. The following day, the plate was washed four times in TBS-T and incubated with 100 µl/well of 0.5 µg/ml of mouse anti-Syn-1 (610787, BD) used as detection antibody, diluted in 100 µl of 1X ELISPOT for 2 h at room temperature. After six washes in TBS-T, the plate was incubated with 1:50,000 goat anti-mouse-HRP (Sigma-Aldrich) secondary antibody diluted in 100 µl of 1X ELISPOT for 2 h at room temperature. Finally, after eight washes in TBS-T, the plate was incubated with 100 µl/well of 1X TMB substrate solution (00-4201-56, Invitrogen) for 20 min at room temperature in the dark, following the addition of 50 µl/well of 2 N H_2_SO_4_ to stop the colorimetric reaction and the absorbance was measured at Victor multi-wells plate reader. For the analysis, the signal at 450 nm was corrected by subtracting the absorbance measured at 560 nm and the signal measured in the wells where only the antibodies were incubated without any protein sample.

### Immunofluorescence and confocal microscopy

Rat and mouse primary cortical neurons and BE(2)-M17 cells were fixed using 4% paraformaldehyde (PFA, Sigma-Aldrich) in PBS pH 7.4 for 20 min at room temperature. Cells were permeabilized in PBS-0.3% Triton-X for 5 min, followed by a 1-h saturation step in blocking buffer [1% Bovine serum Albumin (BSA) Fraction V, 2% goat serum for neurons or FBS for BE(2)-M17 cells, 0.1% Triton-X and 50 mM Glycine in PBS]. Incubations with primary and secondary antibodies were performed in working solution (1:5 dilution of the blocking buffer) for 1 h at room temperature, following both incubations with three washing steps in working solution. The following antibodies were used: mouse anti-pSer129 81/A (825702, BioLegend), mouse anti-Syn-1 (610787, BD), chicken anti-β-Tubulin III (302 306, SYSY), rabbit anti-αSyn pSer129 EP1536Y (ab51253, Abcam), mouse anti-aggregated αSyn SynO2 (847601, BioLegend), rabbit anti-aggregated αSyn MJFR14-6-4-2 (ab209538, Abcam), mouse anti-Bassoon (ab82958, Abcam), rabbit anti-VAMP2 (gifted by Prof. Montecucco’s lab, UNIPD), rabbit anti-αSyn MJFR1 (ab138501, Abcam), rat anti-LAMP1 [1D4B] (ab25245, Abcam), rabbit anti-p62 (ab109012, Abcam), mouse anti-Ubiquitin (P4D1 sc-8017, Santa Cruz Biotech.), rabbit anti-TH (AB152, Millipore), rabbit anti-ALDH1A1 (GTX123973, GeneTex), rat anti-DAT (sc-32258, SCBT), goat anti-mouse-Alexa Fluor 488 (A11029, Invitrogen), goat anti-mouse-Alexa Fluor 568 (A11004, Invitrogen), goat anti-rabbit-Alexa Fluor 488 (A11034, Invitrogen), rabbit-Alexa Fluor 568 (A11036, Invitrogen), goat anti-rat-Alexa Fluor 647 (A21247, Invitrogen), goat anti-chicken-Alexa Fluor 647 (A21449, Invitrogen). For the immunolabeling with the anti-pSer129 antibody, an antigen retrieval step was introduced before saturation by incubating cells with citrate buffer (10 mM sodium citrate, 0.05% Tween-20, pH 6) at 90 °C for 5 min or at 70 °C for 10 min. For the nuclei staining, cells were incubated with Hoechst 33258 (Invitrogen, 1:2000 dilution in PBS) for 5 min. Before coverslips mounting on glass slides, cells were washed three times in PBS and rinsed in distilled water. Confocal immunofluorescence z-stack images were acquired on the Olympus Fluoview 1000 laser scanning confocal microscope using a 60X oil immersion objective with numerical aperture 1.42, on the Zeiss LSM700 laser scanning confocal microscope using a 63X oil immersion objective or on the Leica SP5 laser scanning confocal microscope using a 40X oil immersion objective. To image the fluorescence of αSyn immunolabeling at the confocal microscope, the laser intensity was set based on the αSyn-null neurons to acquire αSyn-specific signal.

### Immunofluorescence image analysis

Confocal fluorescence images were processed and analyzed with the Fiji software (https://imagej.net/Fiji). Z-stacks images were converted to maximum intensity z-projections. For the quantification of pSer129 intensity in BE(2)-M17 cells, the Integrated Density (IntDen) was measured on fixed areas in the cytoplasm of transfected cells, normalizing the signal from the immunostaining with the anti-pSer129 antibody to the miniSOG fluorescence (considered as total αSyn). For primary mouse neuron images, immunostaining with anti-β-Tubulin III was used to specifically identify neuronal cells and processes. β-Tubulin III signal was converted to a binary image and used as a mask to measure the neuronal area (converted to µm^2^) for further normalization. For the total (Syn-1 immunolabeling) and aggregated (SynO2 and MJFR1-6-4-2 immunolabeling) αSyn fluorescence intensity, the raw IntDen was normalized to the β-Tubulin III area in the field of view, while levels in the soma were measured as Mean Intensity in the region of interest (ROI). To analyze the αSyn puncta fluorescence intensity in the periphery, a fluorescence threshold was set, and the Analyze Particle tool was used. For the quantification of aggregated αSyn puncta clustering (MJFR14-6-4-2 immunolabeling), the distance among the x,y coordinates of each puncta centroid was measured by the Nearest Neighbor Distance (Nnd) plugin. For the detection of Bassoon-, αSyn- and VAMP2-positive puncta and the percentage of colocalizing puncta, the single channel images were converted to binary images (setting a threshold) and the ComDet v.0.4.2 plugin was used (4.00 pixels particle size, 20.00 intensity threshold, segmentation of larger particles). For the total αSyn and EGFP-CD63 levels in BE(2)-M17-αSyn cells, the Mean Intensity in the cell area (defined as region of interest, ROI) was measured. For the total Ubiquitin fluorescence intensity, the β-Tubulin III mask was subtracted to the Ubiquitin channel and the raw IntDen was normalized to the β-Tubulin III area in the field of view. The Ubiquitin levels in the soma were measured as sum of the IntDen values of the somata in the field of view, normalized to the total area of the somata; the peripheral Ubiquitin signal was obtained by subtracting the normalized fluorescence in the cell bodies to the total per field of view. For the LAMP1-positive endolysosomes in the neurites, the β-Tubulin III and the LAMP1 channels were converted to binary images (setting a threshold). The images were rotated to visualize each neurite horizontally, and the length was calculated considering the neurite sprout from the soma as X = 0 coordinate. Based on the β-Tubulin III binary image, a ROI along the neurite was defined and the LAMP1-positive structures were identified by the Analyze Particle tool (minimum area 0.1 µm^2^) and annotated in terms of number, area, and X, Y coordinates of the centroid. The endolysosomal density in the neurites was determined as number of LAMP1-positive particle/neurite length, whereas the endolysosomal proximity to the soma was calculated as percentage of LAMP1-positive particle with X coordinate <30 µm in each neurite. To analyze the LAMP1- and p62-positive particles in the soma, a fluorescence threshold was set, and the Analyze Particle tool was used to quantify the particle number, mean size (µm^2^), and mean Fluorescence Intensity. For the analysis of the αSyn levels in the mesencephalic neuronal preparations, the Mean Fluorescence Intensity was measured in ROIs corresponding to the somata of DANs and non-DANs. Finally, for the analysis of neuronal arborization of DANs, the TH channel was converted to a binary image and the total neurite length per field of view was measured using the NeuronJ plugin upon manual annotation of each neurite.

### Live neuron time-lapse imaging

For live time-lapse recordings, neurons transfected with the αSyn-TimeSTAMP-YFP-miniSOG construct received a pulse with 1 µM BILN-2061 inhibitor for 4 h in complete medium at 37 °C. After three washes in Hank’s Balanced Salt Solution (HBSS), neurons were incubated with imaging medium (HBSS containing 1X B27 Supplements, 25 mM glucose, 1 mM pyruvate, and 20 mM HEPES) in control conditions or with the addition of DOPAL and/or Nocodazole administration. Neurons were imaged with the inverted Olympus Fluoview 1000 laser scanning confocal microscope using a 40X oil immersion objective lens with numerical aperture 1.3 at 0.2% laser power to minimize photo-toxicity. During the time-lapse recordings, cells were maintained at 37 °C in controlled atmosphere. Z-stack images were acquired with 200 µm of confocal aperture and 1 µm step size (17–24 steps in average) every 30 min for 18 h. Nine different areas were imaged for each independent experiment. Time-lapse movies were edited using Imaris software package. Fluorescence intensity in the cell body was then quantified using IntDen parameter in the Fiji software, whereas the αSyn-positive synapses and relative fluorescence intensity were identified by a custom-made MatLab script (available upon request to the corresponding authors). Only the new terminals identified in the first 3 h of the time-lapse were considered, assuming t0 as common starting point. For the quantification of the αSyn half-life in the soma and synapses in Nocodazole-treated neurons, the fluorescence variations over time in each individual soma and synapse were fitted by a one-phase decay and the mean value ± SEM was calculated.

### CLEM of miniSOG-tagged αSynuclein

Neurons transfected with the αSyn-TimeSTAMP-YFP-miniSOG construct received a pulse with 1 µM BILN-2061 inhibitor for 4 h in complete medium. After three washes in complete medium, neurons were kept in the incubator at 37 °C in fresh complete medium with or without 100 µM DOPAL. After 24 h from the BILN-2061 pulse, neurons were fixed and processed for photo-oxidation and EM processing (next sections below). In the protein fragment complementation assay^41^, neurons that were previously transduced with the two fragments of the αSyn-split-miniSOG, were treated with 100 µM DOPAL in complete medium for 24 h in the incubator at 37 °C. At the end of the treatment, the growth medium was replaced with imaging medium in both the treated and untreated samples and live images of the reconstituted split-miniSOG complex were acquired using the Leica SPE II inverted confocal microscope. Position coordinates were saved. Cells were then fixed in place, and the photo-oxidation protocol followed.

### Confocal fluorescence imaging and photo-oxidation

For the CLEM experiments, cells were fixed using pre-warmed (37 °C) 2.5% (w/v) glutaraldehyde (Electron Microscopy Sciences) in 0.1 M sodium cacodylate buffer, pH 7.4 (Ted Pella Incorporated) for 5 min at room temperature and then transferred on ice for 1 h. Subsequently, cells were rinsed on ice 3–5 times using chilled cacodylate buffer and treated for 30 min with a blocking solution (50 mM glycine, 10 mM KCN, and 5 mM aminotriazole in 0.1 M sodium cacodylate buffer, pH 7.4) to reduce non-specific background precipitation of 3-3’-diaminobenzidine (DAB). Cells were imaged using a Leica SPE II inverted confocal microscope outfitted with a stage chilled to 4 °C and then photo-oxidized using a 150 W Xenon lamp. Confocal fluorescence and transmitted light images were acquired with minimum exposure to identify transfected cells, with care to avoid sample photo-bleaching. For photo-oxidation, oxygenated DAB (Sigma-Aldrich) was dissolved in 0.1 N HCl at a concentration of 5.4 mg/ml and subsequently diluted ten-fold into sodium cacodylate buffer (pH 7.4, with a final buffer concentration of 0.1 M), mixed, and passed through a 0.22 μm syringe filter before use. DAB solutions were freshly prepared on the day of photo-oxidation and placed on ice and protected from light before being added to cells. The cells were then illuminated through a standard FITC filter set (EX470/40, DM510, BA520) for miniSOG photo-oxidation, with 100% intense light from a 150 W xenon lamp. Illumination was stopped as soon as an optically-dense reaction product began to appear in place of the fluorescence, as monitored by transmitted light (typically 3–8 min, depending on the initial fluorescence intensity, the brightness of the illumination, and the optics used). Confocal images were analyzed with the Fiji software.

### Electron microscopy

Multiple areas on a single dish were photo-oxidized as described in the previous section. Subsequently, plates with cells were placed on a bed of ice and washed using ice-cold cacodylate buffer to remove unpolymerized DAB. After washing, cells were post-fixed with 1% osmium tetroxide (Electron Microscopy Sciences) in 0.1 M sodium cacodylate buffer for 30 min on ice, then washed with ice-cold cacodylate buffer (5 times, 1 min each) and rinsed once in ice-cold distilled water. An additional staining step with filtered 2% uranyl acetate (UA) in distilled water was performed by overnight incubation at 4 °C. The day after, the UA was removed and washed-out with ice-cold distilled water (3 times, 3 min each). The samples were then dehydrated with an ice-cold graded ethanol series (20%, 50%, 70%, 90%, and 100% twice) for 3 min each step and washed once in room temperature anhydrous ethanol. Samples were then infiltrated with Durcupan ACM resin (Electron Microscopy Sciences) using a 1:1 solution of anhydrous ethanol:resin for 30 min on a platform with gentle rocking, then with 100% resin overnight with rocking. The next day, the resin was removed from dishes (by decanting and gentle scraping with care to avoid touching cells), replaced with freshly prepared resin (3 times, 30 min each with rocking), and polymerized in a vacuum oven at 60 °C for 48 h. Subsequently, photo-oxidized areas of interest were identified by transmitted light, sawed out using a jeweler’s saw, and mounted on dummy acrylic blocks with cyanoacrylic adhesive. The coverslip was carefully removed, and ultrathin sections (80 nm thick) were cut using a diamond knife (Diatome). Electron micrographs were acquired using a FEI Technai 12 (Spirit) transmission electron microscope operated at 80 kV; micrographs were produced using a Tietz 2k by 2k CCD camera and collected using the SerialEM package. Images were then processed and analyzed using Fiji software. Scale bars were adjusted according to the magnification in each image and ultrastructural elements (synaptic vesicles, ILVs and MVBs) were manually annotated. Synaptic vesicles size was expressed as Feret diameter. For the synaptic vesicles clustering analysis, the distance between each pair of vesicles in each pre-synaptic terminal was determined by a custom-made MatLab script (available upon request to the corresponding authors) using the X, Y coordinates of the centroid of each vesicle. The inter-vesicle distances were then visualized by cumulative frequency (bin width 50 nm).

### Pulse-chase experiments with JF_570_-HaloTag ligand

At 24 h post-transfection, BE(2)-M17 expressing the αSyn(WT)-HaloTag or the αSyn(K96R)-HaloTag constructs, cells were subjected to a pulse with 3 µM JF_570_-HaloTag ligand^44^, diluted in complete medium for 30 min. After extensive washes in HBSS to remove the excess of ligand, cells were incubated with 100 µM DOPAL in OptiMEM. After 48 h, cell medium was washed out and cells were fixed with 4% PFA for 20 min at RT. Samples were then washed with PBS and mounted on glass slides with Gelvatol mounting medium (in-house reagent). Z-stacks of JF_570_-positive cells were acquired with the Olympus Fluoview 1000 laser scanning confocal microscope using a 60X oil immersion objective lens with numerical aperture 1.42. Images were analyzed with the Fiji software and the Analyze Particle plugin was used to study αSyn-HaloTag-positive cytoplasmic inclusions. Alternatively, for the CLEM experiment, cells were fixed with glutaraldehyde following the protocol described for miniSOG, proceeding with the confocal fluorescence imaging at Leica SPEII, photo-oxidation using a ReAsH filter set (P/N:mCherry-A-L01-ZERO, Ex:FF01-562/40(542-582), DM:FF593-Di02, Em:FF01-641/75 (604-679)) and sample processing for EM.

### Pulse-chase experiment with biotin-HaloTag ligand

At 36 h post-transfection, BE(2)-M17 cells expressing the αSyn(WT)-HaloTag construct were treated overnight with 100 µM DOPAL in OptiMEM. At the end of the treatment, cells were subjected to a pulse with 5 µM biotin-HaloTag ligand (G828A, Promega) diluted in complete medium for 3 h. After three washes in HBSS to remove the excess of ligand, cells were incubated with complete growth medium. At different time-points (*t* = 0-4-8-12-24-30 h), cells were collected, spinned down and the cell pellets were stored at −80 °C. At the end of the time course, all samples were thawed, and cell were harvested in lysis buffer (20 mM Tris–HCl pH 7.5, 150 mM NaCl, 1 mM EDTA, 2.5 mM sodium pyrophosphate, 1 mM β-glycerophosphate, 1 mM sodium orthovanadate, 1% Triton® X-100), supplemented with a protease inhibitor cocktail (Sigma-Aldrich). After lysates clarification by centrifugation at 20,000 × *g* at 4 °C, protein concentration was determined with the Pierce® BCA Protein Assay Kit (Thermo Scientific) following the manufacturer’s instructions. 150 μg of proteins for each sample were diluted in TBS buffer (Tris–HCl 50 mM pH 7.4, NaCl 150 mM) to a final volume of 300 μl and incubated with 10 μl of medium slurry of streptavidin-coated magnetic beads (GE Healthcare) for 2 h at room temperature under continuous rotation. The unbounded proteins were then separated by the isolated biotin-labeled αSyn-HaloTag by a magnetic rack and the flow-through was collected separately. The beads were then washed three times with TBS-Urea 2 mM, incubated with 10 μl of loading buffer 1X and boiled at 95 °C for 10 min. Samples were loaded in gradient 4–20% Tris-Glycine-SDS gels (BioRad) and western blot was performed as follows using the anti-αSyn MJFR1 antibody for the immunoblot.

### Exosomes purification and analysis

Exosomes (EXs) were purified from cell culture medium of both BE(2)-M17-αSyn and HEK293T cells transiently transfected with αSyn-EGFP construct and cultured in 15 cm-dishes (two dishes/condition). 48 h post-transfection or dox addition to cell medium, cells were treated with 100 µM DOPAL in OptiMEM (the untreated cells were maintained in OptiMEM overnight as well) for 48 h. For EXs release stimulation, cells were incubated for 30 min in the presence of 2 μM ionomycin (Sigma) at 37 °C, following the addition of 4 mM EGTA (Sigma) to stop the reaction. Cell medium was collected and remaining cells and debris were pelleted by sequential centrifugations at 700 × *g* for 5 min at room temperature and 1500 × *g* for 20 min at 4 °C. Then EXs were isolated form the supernatant by ultracentrifugation at 50,000 × *g* for 2 h (in 24 ml tubes, rotor 70Ti, XL90 Ultracentrifuge Beckman). The resulting EXs pellet was lysed in 50 μl of RIPA buffer supplemented with protease inhibitors and analyzed by Western blot for αSyn content and exosomal markers Flotillin-1, HSP70, HSP90, Alix. For the limited proteolysis assay, PK was serially diluted and added to EX samples to final concentrations of 0-0.5-1.5 μg/ml. The samples were then incubated at 37 °C for 30 min, following by 5 mM phenylmethylsulfonyl fluoride (PMSF) addition. For detergent treatment, Triton® X-100 (1%) was added to the vesicle preparations. The samples were then incubated on ice for 30 min before they were subjected to proteinase K digestion as described above. Samples were then analyzed by Western blot with the anti-αSyn MJFR1, anti-HSP90, and anti-CD9 antibodies. For the ultrastructural analysis, EXs resuspended in PBS were absorbed onto a carbon-coated copper grid and were then negative stained with 0.05% uranyl acetate solution. EXs were imaged with a Tecnai G2 (FEI) TEM operating at 100 kV and images were captured with a Veleta (Olympus Soft Imaging System) digital camera. Image analysis was performed using Fiji software, using the Feret diameter and circularity parameters to study EXs size and shape, respectively.

### *Aldh1a1*^*−/−*^*/Aldh2*^*−/−*^ mice and brain tissue preparation

Mice with homozygous deletions of both *Aldh1a1* and *Aldh2* genes (double knock-out mice) on a C57BL/6 background were generated and genotyped as previously described^48^. Animals were maintained and experiments were conducted according to the National Institute of Health “Guide for the Care and Use of Laboratory Animals” and the approval by the Institutional Animal Care and Use Committee of the University of Texas Health Science Center at San Antonio. Age-matched male *Aldh1a1*^*−/−*^*/Aldh2*^*−/−*^ and wild-type mice were sacrificed between 11 and 12 months of age. Brains were rapidly collected following brief carbon dioxide anesthesia and decapitation. For western blot assays, brain tissues were rapidly dissected on an ice-cold glass plate, collecting the midbrain and striatum separately; tissues were then snap-frozen on dry ice and transferred to a −80 °C freezer for storage until assayed. Samples were homogenized in freshly prepared ice-cold RIPA buffer (Cell Signaling Technology) and supplemented with phosphatase inhibitors cocktail (Life Technologies) and protease inhibitors (Roche) cocktails. Clarified lysates were then analyzed by SDS-PAGE and immunoblotting.

### Zebrafish treatment

Wild-type zebrafish were staged and maintained according to standard procedures^88^. Embryos were obtained by natural mating and raised at 28.5 °C in Petri dishes containing fish water [50X: 25 g Instant Ocean (Aquarium Systems, SS15-10), 39.25 g CaSO_4_ and 5 g NaHCO_3_ for 1 l] with a photoperiod of 13 h light/11 h dark. All zebrafish experiments were carried out at the Zebrafish Facility of the University of Padova (Italy), in accordance with the European (EU 2010/63 directive) and Italian Legislations, under authorization number 407/2015-PR from the Italian Ministry of Health. Randomly chosen zebrafish larvae were exposed to 0.2–0.35 µM disulfiram (86720, Sigma-Aldrich) from 2 days post-fertilization (dpf) to 6 dpf, refreshing the treatment every 24 h in the morning. The compound was freshly dissolved in DMSO (also used as control) in 20,000X stocks to avoid vehicle-induced toxicity. At 6 dpf, zebrafish larvae were subjected to behavioral analysis followed by whole-mount immunostaining. Alternatively, larvae were euthanized with 0.3 mg/ml tricaine added in fish water and zebrafish heads were isolated by decapitation. The isolated tissues (about 30 heads each group) were homogenized in RIPA buffer supplemented with PMSF and protease inhibitor cocktails (Roche) in ice. Lysates were clarified by centrifugation and stored at −80 °C until western blot analysis as described above, using the mouse anti-βActin and rabbit anti-α/β-Synuclein as primary antibodies.

### Swimming behavioral analysis

At 6 dpf, zebrafish larvae were transferred to a 48-wells plate (Sarstedt) 1 h prior to behavioral analysis. Swimming performance was studied using a DanioVision™ automated tracking system from Noldus Information Technology (Wageningen, the Netherlands) during the light-dark routine according to Lulla et al.^54^, with slight modification. Briefly, larvae were placed inside the Danio Vision Observation chamber (maintained at 28 °C) for 30 min in the dark for habituation. The swimming activity was then assessed during three cycles of alternating 10-min light and dark periods. The acquired track files were analyzed using the Ethovision X.T. 8.5.614 software (Noldus Information Technology, Wageningen, the Netherlands) for the total distance moved on periods of 2 min. Also, the delta distance moved during the light-to-dark stimuli and the total distance moved over the 60 min of acquisition were compared among treatments. The experiment was repeated 7 times, using *n* = 8–16 larvae per group for each biological replicate. For the autocorrelation analysis, about 15 larvae per condition from three independent experiments were analyzed. The velocity tracks (10 second-bin) of the movement acquired during the light-dark routine test were analyzed by Simple Time Series Analysis plugin of OriginPro 2020 with Bartlett’s approximation and plotted as AutoCorrelation Function (ACF) ± SEM. Raw data are reported in Supplementary File 6.

### Zebrafish whole-mount immunofluorescence

After swimming behavioral analysis, zebrafish larvae were euthanized with 0.3 mg/ml tricaine and fixed overnight at 4 °C in 4% PFA (Sigma) in PBS while still in the 48-wells plate. Larvae were then post-fixed in 100% methanol and separately transferred to 1.5 ml-tubes keeping track of their position in the plate of the behavioral analysis. After rehydration with graded methanol series (75-50-25% in PBS) and wash in 0.2% Triton in PBS (PBT) for 5 min at room temperature, larvae were incubated in 3% H_2_O_2_–5% KOH in water for 20 min at room temperature for depigmentation. Larvae were then permeabilized in ice-cold 100% acetone for 15 min at −20 °C, following washes in distilled water and PBT. After a saturation step in 1% BSA, 2% goat serum in PBT (PBT-B) for 4 h at room temperature, larvae where incubated with rabbit anti-TH primary antibody (AB152, Millipore) diluted 1:100 in PBT-B for 24 h at 4 °C. The following day, larvae were washed 4 times in PBT for 20 min each at room temperature prior to overnight incubation with goat anti-rabbit Alexa Fluor 488 (A11034, Invitrogen) diluted 1:100 in PBT-B at 4 °C in the dark. After extensive washes in PBT, larvae were embedded in 0.8% low-melting agarose and placed on depression slides. TH-positive neuronal clusters in the brain were imaged by z-stacks (about 25 steps, 10 µm step size) under a 10X dry objective at the Zeiss LSM700 laser scanning confocal microscope. The acquired images were then analyzed with Fiji software. The fluorescence intensity (maximum intensity z-projections) of the TH-positive neuronal clusters in the diencephalon (DE) and the olfactory bulb & telencephalon (OB&TE) was measured as IntDen on a fixed area among samples, subtracting the background signal to each image.

### Synuclein sequences alignment

Protein sequences of mammalian αSyn (P37840_Human, O55042_Mouse, P37377_Rat) and zebrafish Synuclein isoforms (Q7SX92_Sncb, Q568R9_Sncga, Q502J6_Sncgb) were obtained from UniProtKB (https://www.uniprot.org). Multiple sequence alignment was performed by CLUSTALW (https://www.genome.jp/tools-bin/clustalw) and visualized by Jalview software (https://www.jalview.org).

### Generation of zebrafish F0 knock-out for *synuclein* genes

Genetic ablation of *sncga* and *sncb* genes in zebrafish wild-type larvae was obtained by CRISPR-Cas9 strategy according to Kroll et al.^57^, with slight modifications. Three guide RNAs (gRNAs) for each gene were designed to target three different exons to increase the efficiency of knock-out (see Supplementary File 1 for details). At the day of injection, gRNAs-Cas9 complexes were freshly prepared. Briefly, 10 μM sgRNA (purchased at Synthego) were prepared and the three sgRNAs were pooled together in equal ratio for each gene. 1 μl of sgRNAs mix was then added to 1 μl of 10 μM Alt-R S.p. HiFi Cas9 Nuclease V3 (#1081060, IDT) and diluted with water to a final volume of 2.4 μl. sgRNAs-Cas9 complexes were incubated at 37 °C for 5 min, cooled in ice following the addition of 0.3 μl Danieau buffer 10X and 0.3 μl Phenol Red 10X. For each group, about 120 larvae were injected at single-cell embryonic stage. After screening for fertilized eggs, non-injected, *sncga*-KO and *sncb*-KO embryos were treated with DMSO or 0.2 μM disulfiram from 2 dpf to 5 dpf, refreshing the treatment every 24 h in the morning. At 5 dpf, larvae were either analyzed at Danio Vision for the swimming behavior or decapitated to analyze Synuclein levels in the heads by Western blot, as described above. In addition, about 15 heads/group (untreated) were harvested at 5 dpf to analyze *sncga* and *sncb* mRNA levels. Specifically, samples were lysed in 500 μl of Trizol and RNA was extracted using the miRNeasy kit (1038703, Qiagen) according to manufacturer’s instructions. 1 μg/sample of purified mRNA was then incubated at 37 °C for 30 min with RQ1 RNase-Free DNase (M6101, Promega) and cDNA synthesis was obtained using the MultiScribe™ Reverse Transcriptase kit (4311235, Thermo Fisher). *sncga* and *sncb* expression levels were measured by real-time PCR with SybrGreen method of CFX384 Touch-Real Time PCR Detection System (BioRad) and the 5x HOT FIREPol EvaGreen qPCR Mix Plus (08-36-00001, Solis BioDyne). The amplification protocol consisted of 95 °C for 12 min followed by 40 cycles at 95 °C for 15 s, 60 °C for 25 s, and 72 °C for 25 s. *rplp0* and *eef1a1l1* genes were used as internal standards in each sample. Threshold cycles (Ct) and melting curves were generated automatically by CFX384 Touch-Real Time PCR Detection System (BioRad). Relative gene expression levels were quantified using the comparative Ct method (2^–ΔΔCt^), analyzing each gene in triplicate from samples of three independent experiments. Primer sequences for all genes tested are reported in Supplementary File 1.

### Purified recombinant rabbit monoclonal antibody against DOPAL-modified αSyn

Rabbit monoclonal antibody against DOPAL-modified αSyn (OBI-1-F1-10) was developed by the University of Padova and Abcam, Inc. (Cambridge, UK). Briefly, αSyn-derived peptides containing the KTKEGV repeat were synthetized and covalently modified on a lysine residue by DOPAC (850217, Sigma-Aldrich) through an amide bond (Supplementary Table 1). Two versions were generated by the addition of a Cys-amino hexanoic acid moiety either on the N-terminus or the C-terminus, while the remaining free terminal residues were blocked by an N-terminal acetyl group or a C-terminal amide. Peptides were synthetized, modified, purified by RP-HPLC and confirmed by mass spectrometry by the Peptide Synthesis Facility at UNIPD. Peptides were then conjugated to keyhole limpet hemocyanin (KLH), BSA or blue carrier (BC) proteins (Thermo Scientific) via the cysteine residue at the N-terminus or C-terminus prior to immunization.

Two New Zealand White three-months old female rabbits were immunized with the two antigens using a standard protocol consisting of four injections, a control bleeding and a pre-bleeding boost. All animal procedures were approved by the University of Padova and the Italian National Ministry of Health under authorization number 335/2019-PR. Briefly, two subcutaneous injections were performed using the KLH-conjugated antigens, followed by two subcutaneous injections using the BSA-conjugated antigens. For each injection, a new aliquot of each antigen was thawed and combined with Complete Freund’s Adjuvant (Sigma-Aldrich) (1:1 v/v, initial immunization) or with incomplete Freund’s Adjuvant (1:1 v/v, for the subsequent injections). Serum bleeds of 5 ml were obtained after the fourth immunizations and antibody response was evaluated by dot-blot. Both rabbits were chosen for monoclonal antibody generation and were intravenously boosted with BC (Blue Carrier) immunogen 4 days prior to splenectomy. Splenocytes were harvested from the immunized rabbits, frozen in FBS supplemented with 10% DMSO and stored at −80 °C until used.

Splenocytes were then received and processed at Abcam for hybridoma fusion and positive selection following their standardized protocols. Briefly, after splenocytes fusion with rabbit plasmacytoma cells 240E-W2 and selection by HAT (hypoxanthine, aminopterin, and thymidine), supernatants from 30 clones were collected and screened for antigen binding by ELISA by Abcam and by western blot by UNIPD. OBI-1-F1-10 hybridoma was finally selected for expansion, cloning, and recombinant purification.

### Human samples

*Post-mortem* human striatal tissues from six idiopathic PD patients (iPD) and six age-matched healthy controls (HC) were obtained from Queen Square Brain Bank (London, UK). Caudate and putamen were isolated and homogenized in RIPA buffer supplemented with protease and phosphatase inhibitors (Roche) and stored at −80 °C until western blot analysis as described above. *Post-mortem* human brains were collected under human tissue authority license #12198. Limited sample demographics are listed in Supplementary Table 2 and detailed in Mamais et al.^89^.

### Image preparation and statistical analysis

All images were prepared for illustration using Adobe Illustrator CC 2015. The quantitative and statistical analysis of the collected data, as well as the proper graphical visualization, were performed by the GraphPad Prism Software Inc. (version 7). In general, data were collected from at least *n* = 3 independent experiments, with multiple technical replicates for each group. Within each biological replicate, data were normalized to the mean value of the untreated condition and plotted as fold-change variations of the treated samples compared to controls. Data from independent experiments were then pooled together and represented as box plots showing median, 25th and 75th percentiles, minimum and maximum values. The statistical analysis among mean values was performed by two-tailed non-parametric Mann–Whitney test (two datasets) or by the two-tailed non-parametric Kruskall–Wallis test with the Dunn’s multiple comparisons test (more than two datasets). For grouped analysis, the two-way ANOVA test with the Sidak’s or Tukey’s or Bonferroni’s multiple comparison test was performed. Statistical significance was defined for *p*-value < 0.05 (ns *p* > 0.05, *p < 0.05, **p < 0.01, ***p < 0.001, ****p < 0.0001). Additional details on the data analysis are reported in the legend of each figure and summarized in Supplementary File 7.

### Reporting summary

Further information on research design is available in the Nature Research Reporting Summary linked to this article.

## Supplementary information

Supplementary File 1

Supplementary File 6

Supplementary File 7

Reporting Summary

Supplementary File 2

Supplementary File 3

Supplementary File 4

Supplementary File 5

Supplementary Files

## Data Availability

All data generated or analyzed are included in the article and its Supplementary Information files. Additional datasets and analyses, as well as unique reagents (e.g., plasmids) and relevant information reported in this paper will be shared by the corresponding authors upon request.

## Code availability

Original codes generated in this study are available from the corresponding authors upon request.
