# Supplementary material for: DOPAL initiates αSynuclein-dependent impaired proteostasis and degeneration of neuronal projections in Parkinson’s disease
Source: NPJ Parkinsons Dis. 2023 Mar 25;9:42. doi: 10.1038/s41531-023-00485-1 (PMC10039907; doi:10.1038/s41531-023-00485-1)
Supplement: Supplementary file 1 [file 41531_2023_485_MOESM1_ESM.pdf]

Supplementary Figures

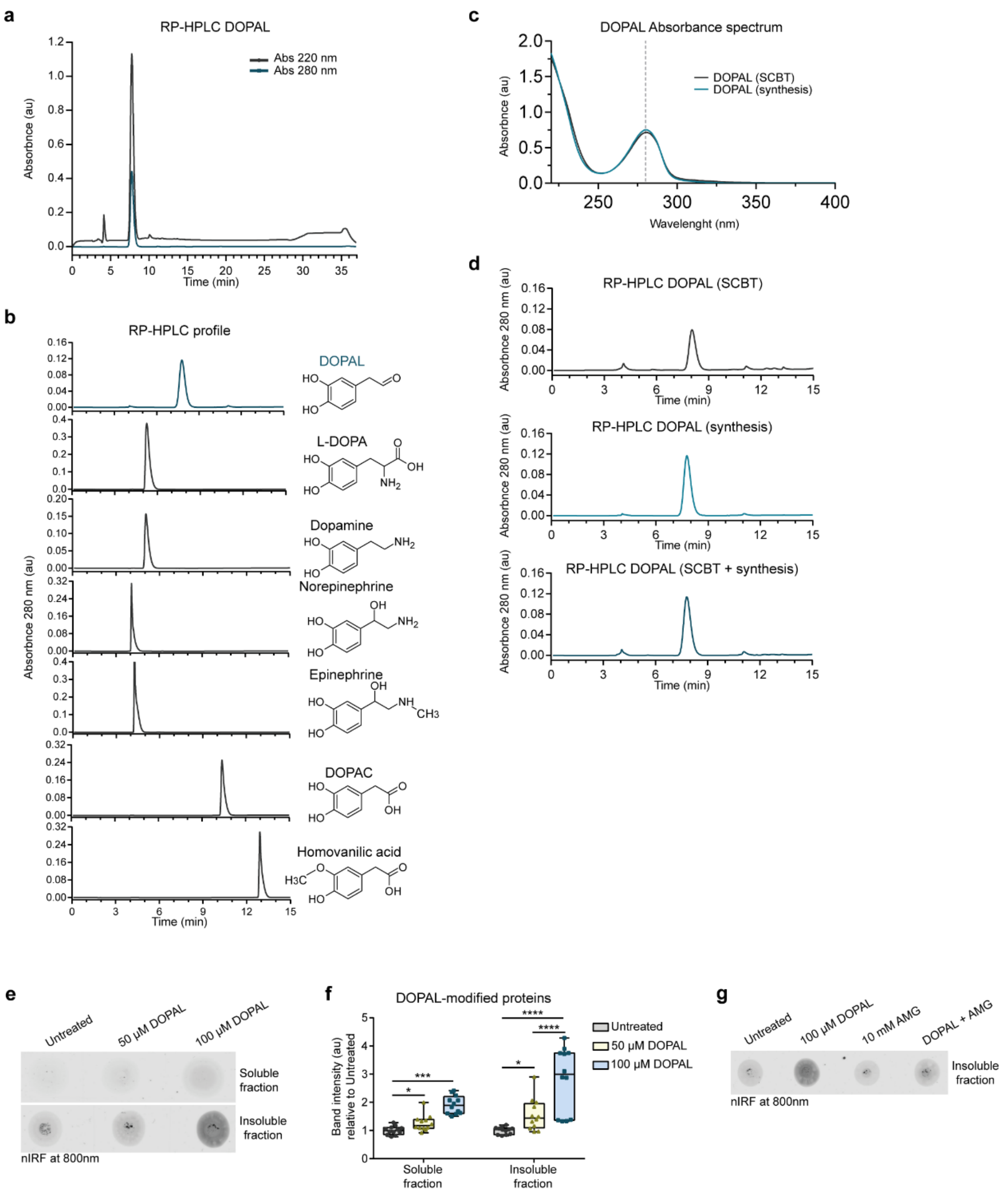

Supplementary Figure 1 DOPAL quality control analysis and cellular treatments **a** RP-HPLC of the synthetized DOPAL with the absorbance profiles at 220 nm (in grey) and 280 nm (in green). **b** Comparison of the RP-HPLC retention time and profile (absorbance at 280 nm) among the synthetized DOPAL and other catechols. The chemical structures of the different molecules are indicated on the right side of the corresponding RP-HPLC profile. **c** Overlap between the absorbance spectra of the synthetized DOPAL (in green) and the DOPAL purchased from Santa Cruz BioTech. (SCBT, in grey). The dotted line indicates the absorbance peak at 280 nm. **d** Comparison of the RP-HPLC retention time and profile among the synthetized DOPAL, the DOPAL by SCBT and the combination of the two. **e** DOPAL-derived nIRF signal in detergent-soluble and insoluble fractions of BE(2)-M17 after an overnight treatment with 0-50-100 µM DOPAL, and **f** corresponding quantification. Data from three independent experiments (with n=4 technical replicates each, pooled together) are normalized to each untreated sample and analyzed by Two-way ANOVA with Sidak's multiple comparison test (* $p<0.05$, *** $p<0.001$, **** $p<0.0001$). Data are displayed as box and whiskers plot showing the minimum and maximum points (whiskers), the first quartile, median and third quartile (box lines). **g** DOPAL-derived nIRF signal in detergent-insoluble fraction of BE(2)-M17 after an overnight treatment with 100 µM DOPAL or the co-treatment with 10 mM AMG.

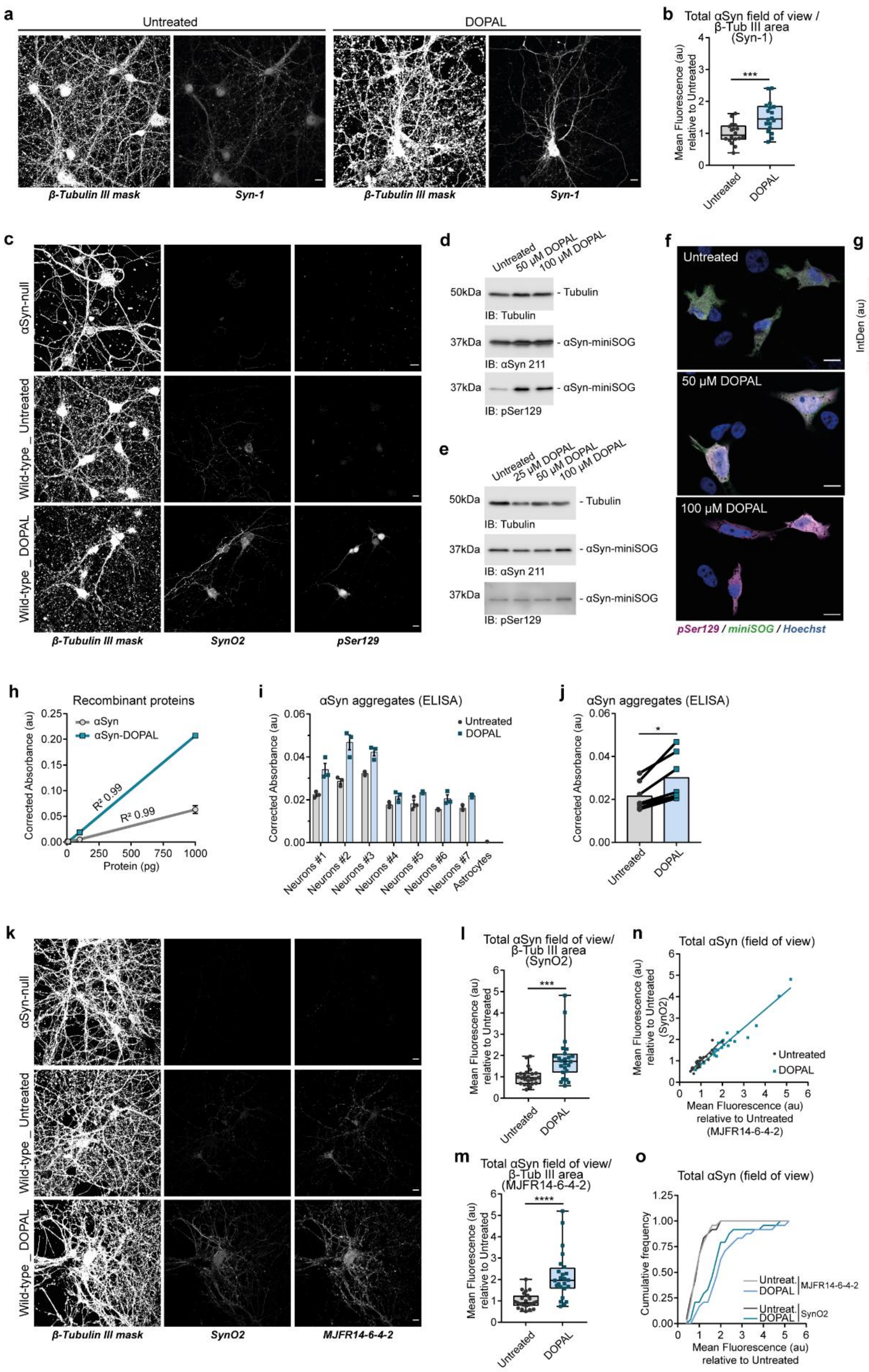
a
Untreated
DOPAL
β-Tubulin III mask
Syn-1
b
Total αSyn field of view / β-Tub III area (Syn-1)
Mean Fluorescence (au) relative to Untreated
***
Untreated
DOPAL
c
αSyn-null
Wild-type _ Untreated
Wild-type _ DOPAL
β-Tubulin III mask
SynO2
pSer129
d
Untreated
50 μM DOPAL
100 μM DOPAL
50kDa
- Tubulin
IB: Tubulin
37kDa
- αSyn-miniSOG
IB: αSyn 211
IB: pSer129
e
25 μM DOPAL
f
50 μM DOPAL
100 μM DOPAL
pSer129 / miniSOG / Hoechst
g
pSer129 / miniSOG
IntDen (au) relative to Untreated
h
Recombinant proteins
Corrected Absorbance (au)
αSyn
αSyn-DOPAL
R² 0.99
Protein (pg)
i
αSyn aggregates (ELISA)
Neurons #1
Neurons #2
Neurons #3
Neurons #4
Neurons #5
Neurons #6
Neurons #7
Astrocytes
j
αSyn aggregates (ELISA)
k
MJFR14-6-4-2
l
Total αSyn field of view/ β-Tub III area (SynO2)
m
Total αSyn field of view/ β-Tub III area (MJFR14-6-4-2)
****
n
Total αSyn (field of view)
Mean Fluorescence (au) relative to Untreated (SynO2)
Mean Fluorescence (au) relative to Untreated (MJFR14-6-4-2)
o
Total αSyn (field of view)
Cumulative frequency
Untreat.
SynO2
Mean Fluorescence (au) relative to Untreated

Supplementary Figure 2 DOPAL induces αSynuclein accumulation, increased phosphorylation at Serine 129 and aggregation in neurons **a** Immunostaining of β-Tubulin III (converted to binary mask) and total αSyn (Syn-1) in untreated and 100 μM DOPAL-treated (for 24 hours) wild-type primary mouse cortical (referred to Fig. 1b). Scale bar: 10 μm. **b** DOPAL-induced αSyn accumulation is expressed as total αSyn fluorescence normalized to β-Tubulin III-positive area. **c** Immunostaining of β-Tubulin III (converted to binary mask), oligomeric (SynO2) and phosphorylated (pSer129) αSyn in untreated αSyn-null and wild-type, and 100 μM DOPAL-treated (for 24 hours) wild-type primary mouse cortical neurons. DOPAL dose-dependent increase of αSyn phosphorylation at Ser129 analyzed by western blot in **d** BE(2)-M17 cells and **e** primary rat cortical neurons overexpressing αSyn-miniSOG after an overnight treatment with 0-25-50-100 μM DOPAL. **f-g** In the same experimental condition, the increased pSer129 was confirmed in BE(2)-M17 cells overexpressing αSyn-miniSOG by immunofluorescence. The fluorescence intensity of the immunolabeling with the anti-pSer129 antibody normalized to the miniSOG fluorescence in each cell. Nuclei are stained with Hoechst (blue). Scale bar: 10 μm. **h** Positive control of ELISA assay with 1-10-100-1000 pg of recombinant αSyn monomer and αSyn-DOPAL oligomers. **i-j** Quantification of αSyn aggregates in neuron lysates measured by ELISA assay. **k** Immunostaining of β-Tubulin III (converted to binary mask), aggregated αSyn (SynO2 and MJFR14-6-4-2) in untreated αSyn-null and wild-type, and 100 μM DOPAL-treated (for 24 hours) wild-type primary mouse cortical neurons (referred to Fig. 1f). Scale bar: 10 μm. **l-m** DOPAL-induced multimeric αSyn accumulation is expressed as αSyn fluorescence normalized to β-Tubulin III-positive area for both immunostainings with the two antibodies. **b,j,l,m** Data from three independent cultures are normalized to each untreated sample, pooled together, and analyzed by Mann-Whitney non-parametric test (* $p<0.05$, ** $p<0.01$, *** $p<0.001$, **** $p<0.0001$). **g** Data from three independent experiments are normalized to each untreated sample, pooled together, and analyzed by Kruskall-Wallis non-parametric test with Dunn's multiple comparison test (* $p<0.05$, ** $p<0.01$). **n** Linear correlation between the DOPAL-induced increased fluorescence intensities derived from the immunostaining with SynO2 and MJFR14-6-4-2 antibodies measured in the entire field of view (total). Untreated: slope 0.0977 ± 0.09, R2 0.83; DOPAL: slope 0.853 ± 0.095 R2 0.94. **o** Cumulative frequency distribution of fluorescence intensities derived from the immunostaining with SynO2 and MJFR14-6-4-2 antibodies measured in the entire field of view (total αSyn). Data are analyzed by Two-way ANOVA with Tukey's multiple comparison test: untreated MJFR14-6-4-2/SynO2 vs DOPAL MJFR14-6-4-2/SynO2: **** $p<0.0001$. **b,g,l,m** Data are displayed as box and whiskers plot showing the minimum and maximum points (whiskers), the first quartile, median and third quartile (box lines). **i** Data are shown as Mean ± SEM.

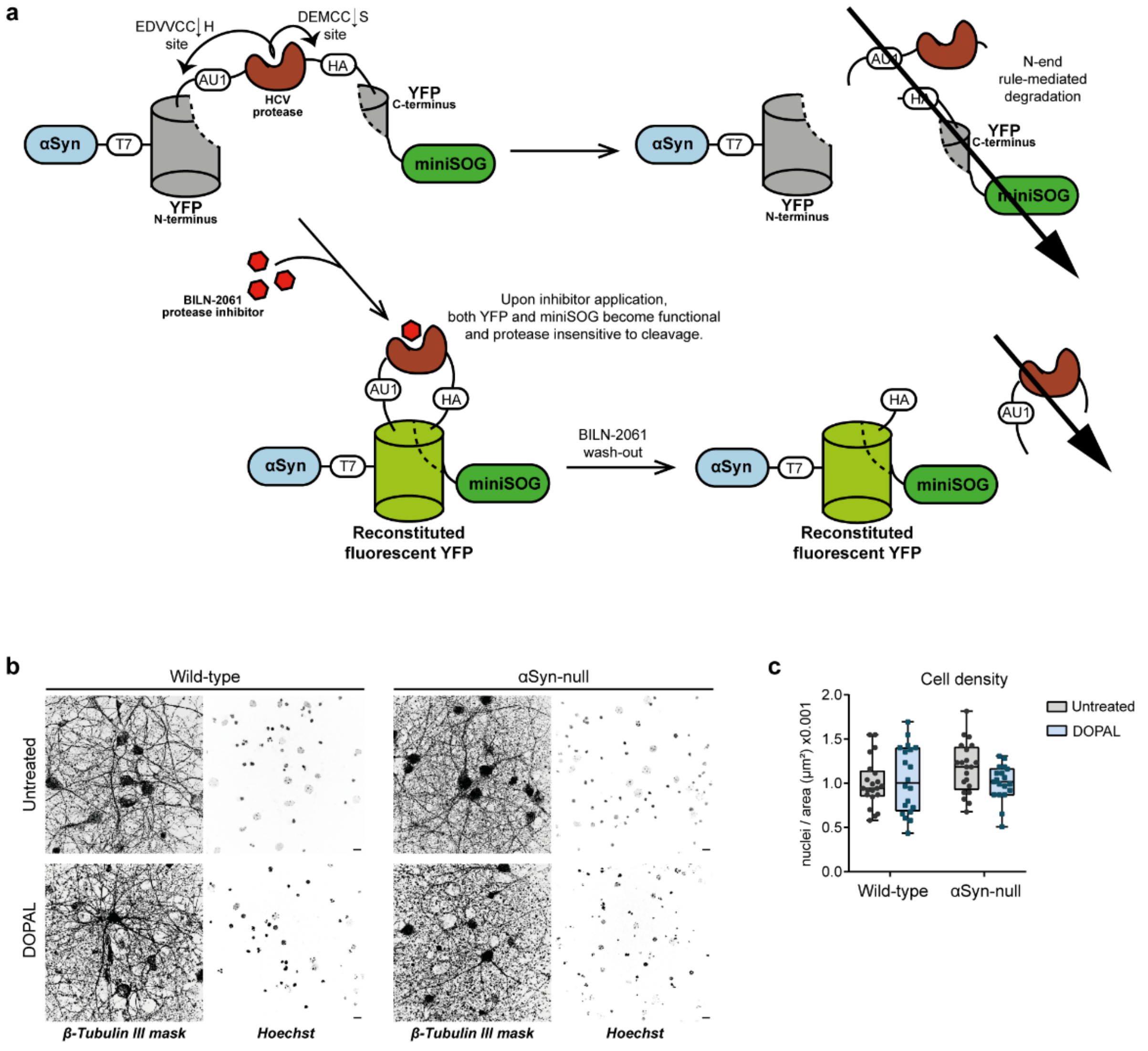

Supplementary Figure 3 a Schematic representation of αSynuclein-TimeSTAMP-YFP-miniSOG. TimeSTAMP (Time-Specific Tag for the Measurement of the Age of Proteins) is a drug-sensitive on-off switch with a cassette comprising the protease domain of hepatitis C virus (HCV) flanked by protease cleavage sites and followed by a split-YFP epitope tag. Upon expression, the protease excises itself and the tag from proteins by default, targeting the two fragments to degradation. However, by applying a cell-permeant protease inhibitor (BILN-2061), αSyn is expressed in frame with a reconstituted and active YFP probe, providing the capability to select and image by LM a newly synthetized protein subpopulation. Here, the TimeSTAMP tag was combined with YFP fluorescent protein and miniSOG, to analyze αSyn trafficking by both live-imaging and CLEM. Adapted from *Butko et al., Nat Neurosci 2012*. **b** Immunostaining of β-Tubulin III (converted to binary mask) and staining with Hoechst to mark up the nuclei in wild-type and αSyn-null primary mouse cortical neurons, both untreated and 100 μM DOPAL-treated for 24 hours. Scale bar: 10 μm. **c** The cell density is expressed as number of nuclei per area (μm2) x0,001. Data are pooled together from three independent experiments and analyzed by Two-way ANOVA with Sidak's multiple comparison test (interaction: ns $p>0.05$; genotype: ns $p>0.05$; treatment: ns $p>0.05$). Data are displayed as box and whiskers plot showing the minimum and maximum points (whiskers), the first quartile, median and third quartile (box lines).

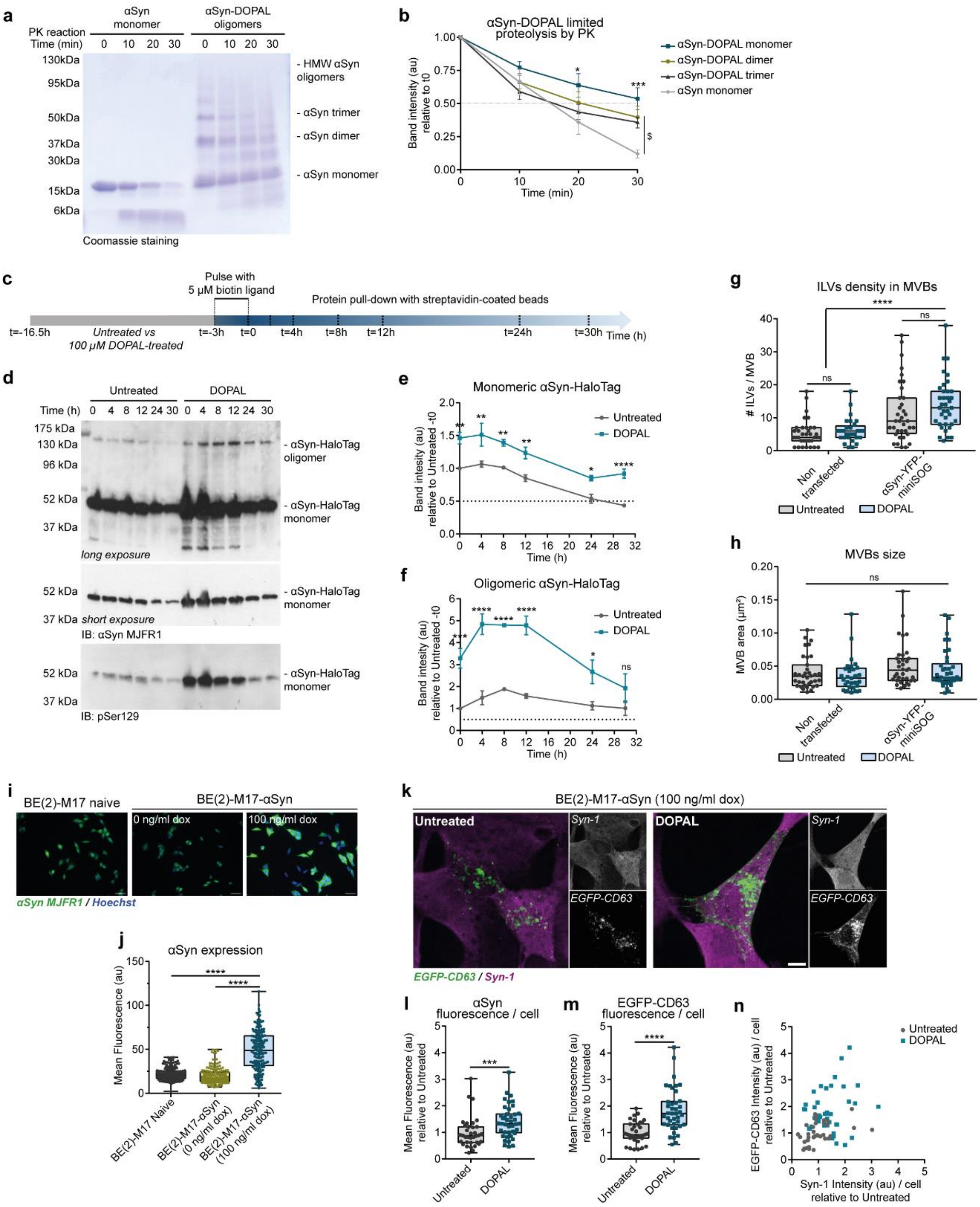

a
αSyn monomer
αSyn-DOPAL oligomers
PK reaction
Time (min) 0 10 20 30 0 10 20 30
130kDa
95kDa
50kDa
37kDa
30kDa
15kDa
6kDa
- HMW αSyn oligomers
- αSyn trimer
- αSyn dimer
- αSyn monomer
Coomassie staining
b
αSyn-DOPAL limited proteolysis by PK
αSyn-DOPAL monomer
αSyn-DOPAL dimer
αSyn-DOPAL trimer
αSyn monomer
Band intensity (au) relative to t0
Time (min)
c
Pulse with 5 µM biotin ligand
Protein pull-down with streptavidin-coated beads
t=-16.5h
Untreated vs 100 µM DOPAL-treated
t=-3h t=0 t=4h t=8h t=12h t=24h t=30h Time (h)
d
Untreated DOPAL
Time (h) 0 4 8 12 24 30 0 4 8 12 24 30
175 kDa
130 kDa
96 kDa
52 kDa
37 kDa
- αSyn-HaloTag oligomer
- αSyn-HaloTag monomer
long exposure
short exposure
IB: αSyn MJFR1
IB: pSer129
e
Monomeric αSyn-HaloTag
Untreated
DOPAL
Band intesity (au) relative to Untreated -t0
Time (h)
f
Oligomeric αSyn-HaloTag
Untreated
DOPAL
Band intesity (au) relative to Untreated -t0
Time (h)
g
ILVs density in MVBs
ILVs / MVB
Non transfected
αSyn-YFP-miniSOG
Untreated
DOPAL
h
MVBs size
MVB area (µm²)
Non transfected
αSyn-YFP-miniSOG
Untreated
DOPAL
i
BE(2)-M17 naive
BE(2)-M17-αSyn
0 ng/ml dox
100 ng/ml dox
αSyn MJFR1 / Hoechst
j
αSyn expression
Mean Fluorescence (au)
BE(2)-M17 Naive
BE(2)-M17-αSyn (0 ng/ml dox)
BE(2)-M17-αSyn (100 ng/ml dox)
k
BE(2)-M17-αSyn (100 ng/ml dox)
Untreated
DOPAL
Syn-1
EGFP-CD63
EGFP-CD63 / Syn-1
l
αSyn fluorescence / cell
Mean Fluorescence (au) relative to Untreated
Untreated
DOPAL
m
EGFP-CD63 fluorescence / cell
Mean Fluorescence (au) relative to Untreated
Untreated
DOPAL
n
Untreated
DOPAL
EGFP-CD63 Intensity (au) / cell relative to Untreated
Syn-1 Intensity (au) / cell relative to Untreated

Supplementary Figure 4 DOPAL affects αSynuclein degradation and promotes MVBs formation **a** SDS-Page and Coomassie staining of the in vitro limited proteolysis assay by PK (0-10-20-30 minutes time-course) of 20 μM recombinant monomeric αSyn and αSyn-DOPAL oligomers obtained by the incubation of 20 μM αSyn with 300 μM DOPAL overnight. **b** In the quantification, the levels of αSyn monomers, dimers and trimers over time are normalized to the amount at t0. Data are presented as mean ± SEM from four independent experiments and analyzed by Two-way ANOVA with Tukey's multiple comparison test (αSyn monomer vs αSyn-DOPAL monomer: * $p<0.05$, *** $p<0.001$; αSyn monomer vs αSyn-DOPAL dimer/trimer: $ $p<0.05$) **c** Schematic representation of the pulse-chase experiment using the biotin HaloTag ligand in αSyn-HaloTag overexpressing BE(2)-M17 cells. **d** Western blot of the degradation of monomeric and oligomeric αSyn(WT)-HaloTag, +/- 100 μM DOPAL treatment. **e-f** In the quantification, the αSyn band intensity was normalized to the starting amount of αSyn monomer / oligomer at t0 in the untreated cells for each αSyn variant. Data are presented as mean ± SEM from n=3 independent experiments and analyzed by Two-way ANOVA with Sidak's multiple comparison test (* $p<0.05$, ** $p<0.01$, *** $p<0.001$, **** $p<0.0001$). In the CLEM experiment of rat primary cortical neurons expressing αSyn-TimeSTAMP-YFP-miniSOG, **g** quantification of ILVs density in MVBs expressed as number of ILVs per MVB and (H) MVBs area. Data are pooled from two independent experiments, three photo-oxidized areas (non-transfected _ untreated: 35 MVBs; non-transfected _ DOPAL-treated: 29 MVBs; αSyn-miniSOG-positive _ untreated: 36 MVBs; αSyn-miniSOG-positive _ DOPAL-treated: 37 MVBs) and analyzed by Two-way ANOVA with Sidak's multiple comparison test (**** $p<0.0001$). **i** Analysis of αSyn overexpression by immunofluorescence (αSyn in green and nuclei staining by Hoechst in blue) in BE(2)-M17-αSyn stable cell line after induction with 100 ng/ml dox, as compared non-induced and naïve BE(2)-M17 cells. **j** In the quantification, αSyn overexpression is expressed as mean fluorescence intensity in each cell. Data are analyzed by Kruskall-Wallis with Dunn's multiple comparison test (**** $p<0.0001$). **k** Immunofluorescence of αSyn levels in 100 ng/ml dox-induced BE(2)-M17-αSyn cells transfected with EGFP-CD63 construct, in untreated and 100μM DOPAL-treated (overnight) condition. Scale bar: 5 μm. In the quantification, **l** αSyn and (M) EGFP-CD63 mean fluorescence were measured and **n** correlated for each cell, revealing a DOPAL-induced accumulation of both αSyn and MVBs. Data are collected from three independent experiments and analyzed by Mann-Whitney non-parametric test (*** $p<0.001$, **** $p<0.0001$). **g,h,j,l,m** Data are displayed as box and whiskers plot showing the minimum and maximum points (whiskers), the first quartile, median and third quartile (box lines).

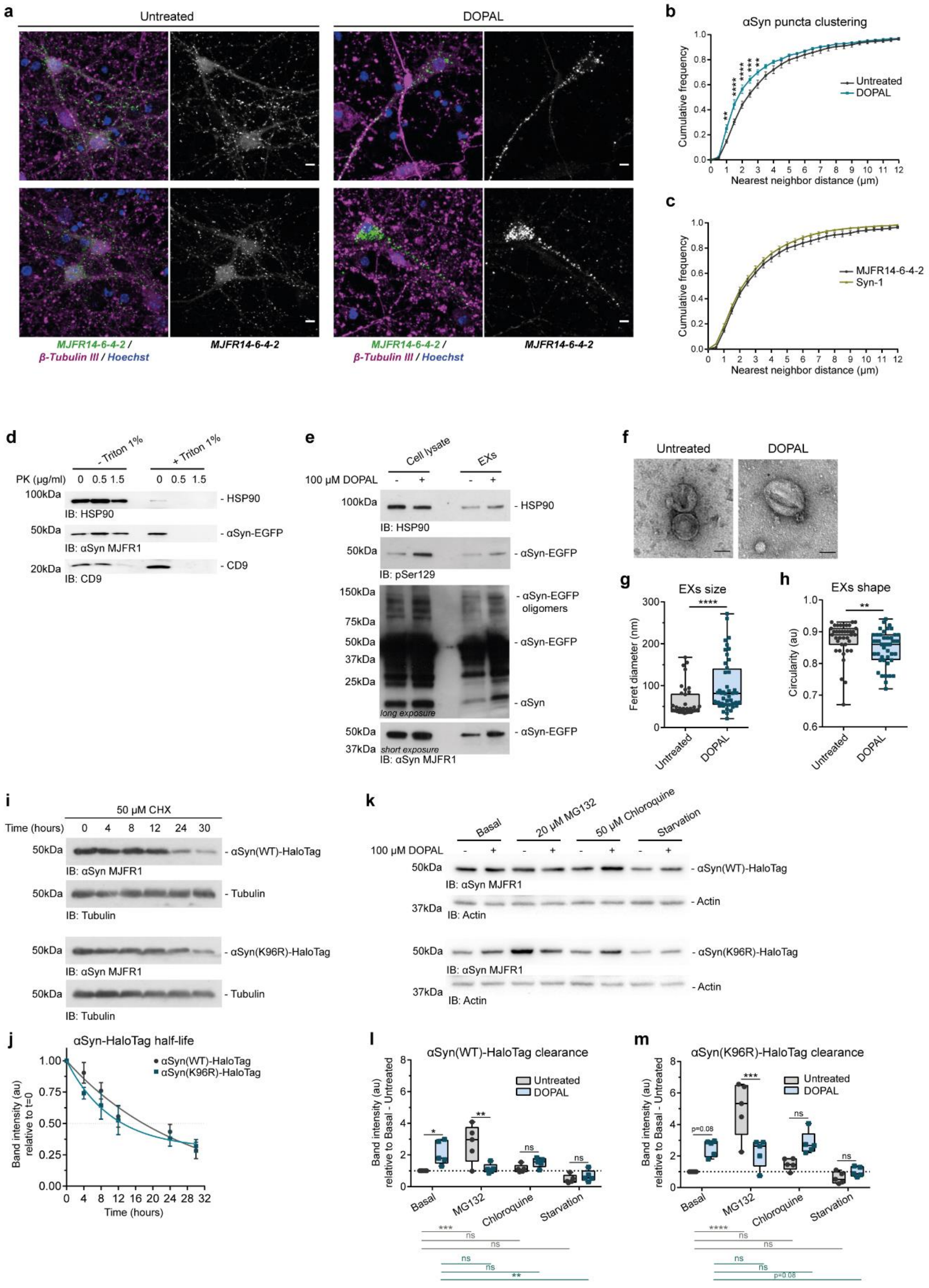

a
Untreated
DOPAL
MJFR14-6-4-2 / β-Tubulin III / Hoechst
MJFR14-6-4-2
b
αSyn puncta clustering
Cumulative frequency
Nearest neighbor distance (µm)
Untreated
DOPAL
c
MJFR14-6-4-2
Syn-1
d
- Triton 1%
+ Triton 1%
PK (µg/ml) 0 0.5 1.5 0 0.5 1.5
100kDa - HSP90
IB: HSP90
50kDa - αSyn-EGFP
IB: αSyn MJFR1
20kDa - CD9
IB: CD9
e
Cell lysate
EXs
100 µM DOPAL - + - +
IB: HSP90
IB: pSer129
αSyn-EGFP oligomers
αSyn
long exposure
short exposure
IB: αSyn MJFR1
f
Untreated
DOPAL
g
EXs size
Feret diameter (nm)
h
EXs shape
Circularity (au)
i
50 µM CHX
Time (hours) 0 4 8 12 24 30
- αSyn(WT)-HaloTag
- Tubulin
IB: Tubulin
- αSyn(K96R)-HaloTag
j
αSyn-HaloTag half-life
αSyn(WT)-HaloTag
αSyn(K96R)-HaloTag
Band intensity (au) relative to t=0
Time (hours)
k
Basal
20 µM MG132
50 µM Chloroquine
Starvation
100 µM DOPAL
- Actin
IB: Actin
l
αSyn(WT)-HaloTag clearance
Band intensity (au) relative to Basal - Untreated
Untreated
DOPAL
Basal
MG132
Chloroquine
Starvation
m
αSyn(K96R)-HaloTag clearance
p=0.08

Supplementary Figure 5 DOPAL promotes αSynuclein loading in the endo-lysosomal pathway **a** Immunostaining of β-Tubulin III (magenta) and aggregated αSyn (MJFR14-6-4-2, green) in untreated and 100 µM DOPAL-treated (for 48 hours) wild-type primary mouse cortical neurons. Nuclei are stained with Hoechst (blue). Scale bar: 5 µm. **b** Clustering of aggregated αSyn-positive puncta is expressed as cumulative frequency of the nearest neighbor distance (µm) between puncta centroids. Data are pooled from three independent cultures and analyzed by Two-way ANOVA with Sidak's multiple comparison test (** p<0.01, *** p<0.001, **** p<0.0001). **c** Conversely the distance distribution among αSyn-positive puncta detected by Syn-1 staining (images refer to Figure 1b) doesn't differ from the puncta detected by the MJFR14-6-4-2 antibody, both analyzed in untreated neurons. **d** Western blot of αSyn-EGFP-containing EXs after incubation with PK at increasing concentrations, while EXs are still intact (- 1 % Triton) or membranes are dissolved by the detergent (+ 1 % Triton). HSP90 is used as intraluminal EXs marker and CD9 as transmembrane protein. **e** Western blot of cell lysates and αSyn-EGFP-containing EXs, purified from the supernatant of untreated and 100µM DOPAL-treated (overnight) HEK293T cells. **f** Representative EM micrographs of EXs purified from cell medium of untreated and DOPAL-treated αSyn-EGFP overexpressing HEK293T cells. Scale bar: 100 nm. In the quantification, **g** EXs size and **h** shape are expressed as Feret diameter and circularity, respectively. Data are pooled from three independent purifications and analyzed by Mann-Whitney non-parametric test (** p<0.01, **** p<0.0001). **i** Time-course experiments in the presence of 50 µg/ml CHX and western blot of BE(2)-M17 cells over-expressing αSyn(WT)-HaloTag and αSyn(K96R)-HaloTag **j** Quantification of αSyn-HaloTag levels at the different time-points, normalized for the relative Tubulin band intensity and for the αSyn-HaloTag amount at t0. Data are presented as Mean ± SEM from n=5 independent experiments per genotype and fitted by one-phase decay equations (WT: y=exp(-0.04x), $t_{1/2}$ 18.8 hours, R^2 0.76; K96R: y=exp(-0.09x)+0.3, $t_{1/2}$ 7.3 hours, R^2 0.70). **k** Western blot of αSyn levels in αSyn(WT)-HaloTag and αSyn(K96R)-HaloTag stable BE(2)-M17 cell lines after +/- 100 µM DOPAL overnight treatment, in basal condition, 20 µM MG132, 50 µM chloroquine and starvation. Relative quantification of **l** αSyn(WT)-HaloTag and **m** αSyn(K96R)-HaloTag levels normalized to untreated – basal condition. Data are collected from five independent experiments and analyzed by Two-way ANOVA with Tukey's multiple comparison test (ns p>0.05, * p<0.05, ** p<0.01, *** p<0.001, **** p<0.0001; untreated vs DOPAL: in black; basal-untreated vs MG132-untreated, chloroquine-untreated, starvation-untreated: in grey; basal-DOPAL vs MG132-DOPAL, chloroquine-DOPAL, starvation-DOPAL: in green). **g,h,l,m** Data are displayed as box and whiskers plot showing the minimum and maximum points (whiskers), the first quartile, median and third quartile (box lines). **b,c,j** Data are shown as Mean ± SEM.

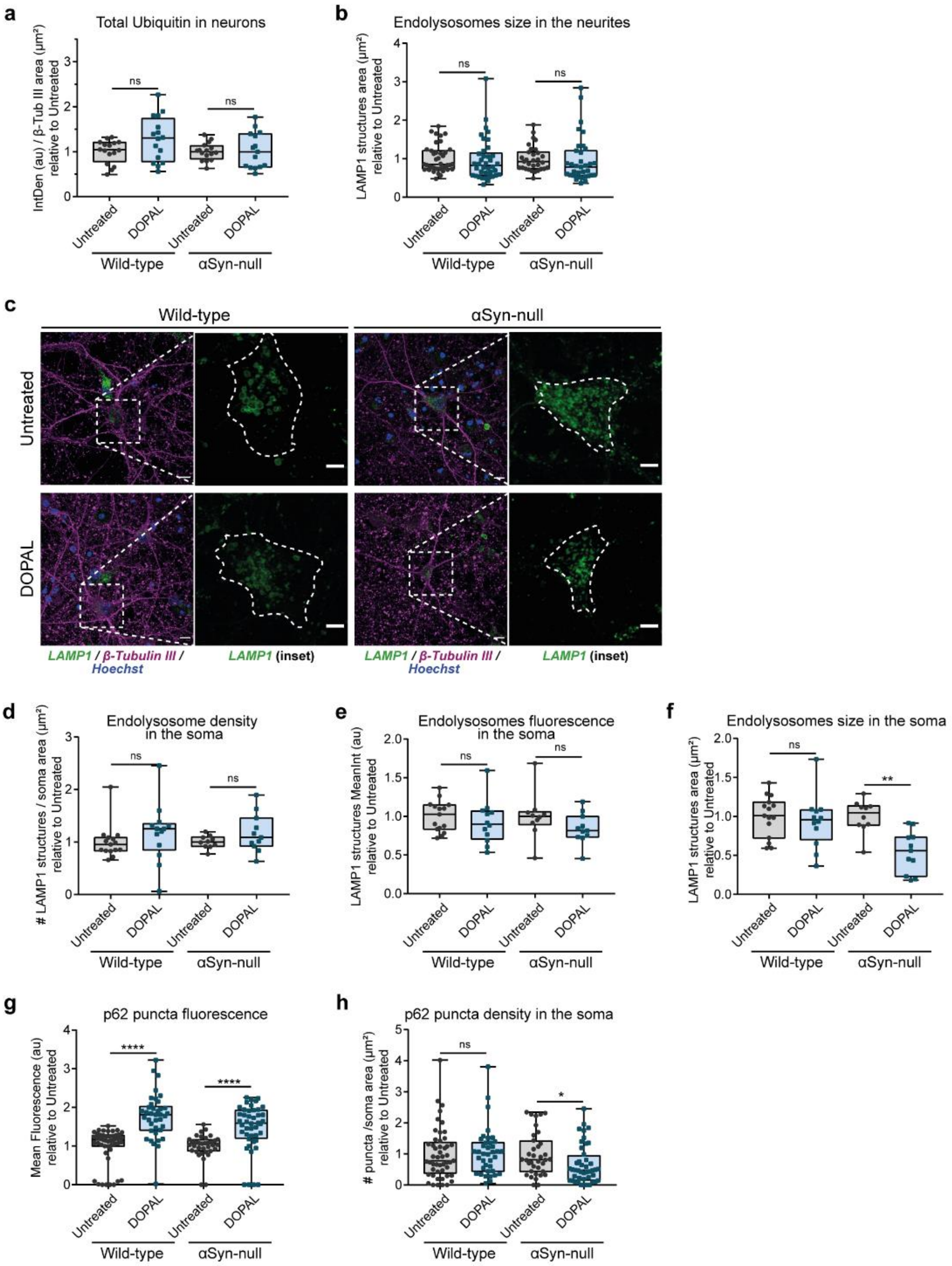

a
Total Ubiquitin in neurons
IntDen (au) / β-Tub III area (µm²) relative to Untreated
ns
ns
Untreated
DOPAL
Untreated
DOPAL
Wild-type
αSyn-null
b
Endolysosomes size in the neurites
LAMP1 structures area (µm²) relative to Untreated
ns
ns
Wild-type
αSyn-null
c
Wild-type
αSyn-null
Untreated
DOPAL
LAMP1 / β-Tubulin III / Hoechst
LAMP1 (inset)
d
Endolysosome density in the soma
LAMP1 structures / soma area (µm²) relative to Untreated
e
Endolysosomes fluorescence in the soma
LAMP1 structures MeanInt (au) relative to Untreated
f
Endolysosomes size in the soma
LAMP1 structures area (µm²) relative to Untreated
**
g
p62 puncta fluorescence
Mean Fluorescence (au) relative to Untreated
****
****
h
p62 puncta density in the soma
puncta /soma area (µm²) relative to Untreated
*

Supplementary Figure 6 Additional quantification of Ubiquitin, LAMP1 and p62 in primary mouse neurons **a** Quantification of the total Ubiquitin fluorescence signal in neurons, normalized to β-Tubulin III area (referred to Fig. 6a). **b** Quantification of the LAMP1 structures mean size (µm2) in each neurite (referred to Fig. 6d). **c** Immunostaining of β-Tubulin III (magenta) and LAMP1 (green) in the soma of untreated and 100 µM DOPAL-treated (for 24 hours) wild-type and αSyn-null primary mouse cortical neurons. Nuclei are stained with Hoechst (blue). Scale bar: 10 µm. In the inset, the LAMP1 fluorescence signal in the cell body is enlarged and the dotted line defines the soma boundaries (scale bar: 5 µm). Quantification of **d** endolysosomes density in the cell body expressed as number of LAMP1 structures / µm2, **e** LAMP1 structures mean fluorescence signal and **f** LAMP1 structures mean size (µm2) in each cell body. Quantification of **g** p62-positive puncta mean fluorescence signal and **h** p62-positive puncta mean size (µm2) in each cell body (referred to Fig. 6g). **a-b, d-h** Data from three independent experiments are normalized to each untreated sample, pooled together, and analyzed by Mann-Whitney non-parametric test (* $p<0.05$, ** $p<0.01$, **** $p<0.0001$). Data are displayed as box and whiskers plot showing the minimum and maximum points (whiskers), the first quartile, median and third quartile (box lines).

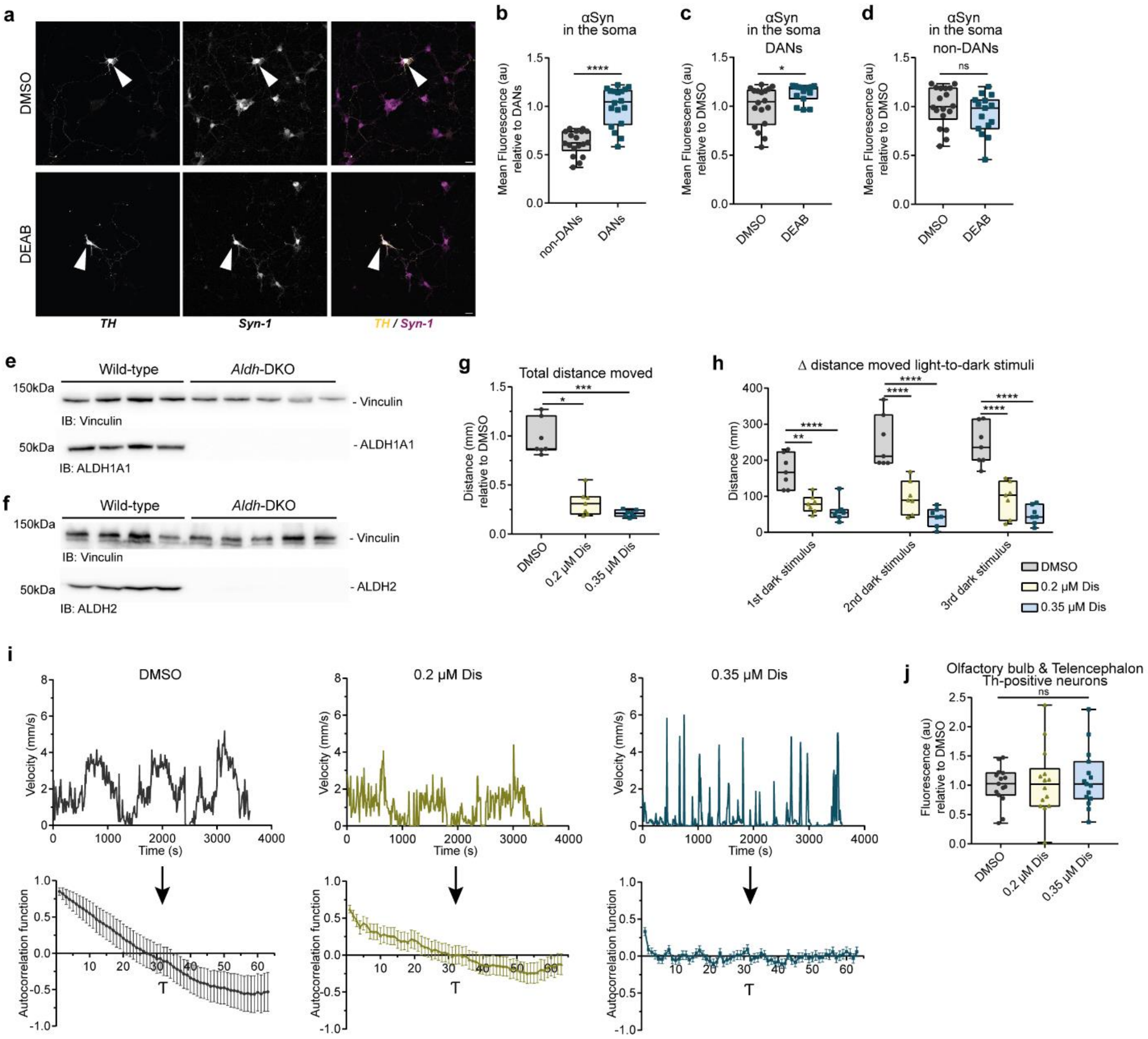

a
DMSO
DEAB
TH
Syn-1
TH / Syn-1
b
αSyn in the soma
Mean Fluorescence (au) relative to DANs
non-DANs
DANs
c
αSyn in the soma DANs
Mean Fluorescence (au) relative to DMSO
d
αSyn in the soma non-DANs
e
Wild-type
Aldh-DKO
150kDa
- Vinculin
IB: Vinculin
50kDa
- ALDH1A1
IB: ALDH1A1
f
- ALDH2
IB: ALDH2
g
Total distance moved
Distance (mm) relative to DMSO
DMSO
0.2 µM Dis
0.35 µM Dis
h
Δ distance moved light-to-dark stimuli
Distance (mm)
1st dark stimulus
2nd dark stimulus
3rd dark stimulus
i
Velocity (mm/s)
Time (s)
Autocorrelation function
τ
j
Olfactory bulb & Telencephalon Th-positive neurons
Fluorescence (au) relative to DMSO

Supplementary Figure 7 Additional data on models of endogenous DOPAL buildup **a** Immunostaining of TH (yellow) and Syn-1 (magenta) from DMSO- and 100 nM DEAB-treated (4 days) wild-type primary mouse mesencephalic neurons. The white arrowheads highlight the soma of dopaminergic TH-positive neurons. Scale bar: 20 μm. Quantification of αSyn fluorescence in the soma in a comparison between **b** non-dopaminergic and dopaminergic neurons in basal condition (DMSO-exposed) **c** DMSO- and DEAB-treated dopaminergic neurons and **d** DMSO- and DEAB-treated non-dopaminergic neurons. Data are normalized to DMSO-treated sample and analyzed by Mann-Whitney non-parametric test (* $p<0.05$, **** $p<0.0001$). Western blot of wild-type (n=4) and ALDH-DKO (n=5) mice **e** striatum and **f** cortex with the antibodies against ALDH1A1 and ALDH2. Immunoblot against Vinculin was used as loading control. **g** Quantification of the total distance moved during the light-dark locomotion test of DMSO-, 0.2 μM Disulfiram- and 0.35 μM Disulfiram-treated zebrafish larvae and **h** the delta distance moved in the first 2 minutes of each light-to-dark stimuli of the swimming behavior analysis (referred to Fig. 8c). Data from seven independent experiments are analyzed by Kruskall-Wallis with Dunn's multiple comparison test and Two-way ANOVA with Tukey's multiple comparison test (* $p<0.05$, ** $p<0.01$, *** $p<0.001$, **** $p<0.0001$). **i** Representative graphs showing the time-course of velocity (10 second-bin) of movement during the light-dark routine test acquired at DanioVision™ and the corresponding autocorrelation function of zebrafish larvae exposed to DMSO, 0.2 μM Disulfiram and 0.35 μM Disulfiram. **j** Quantification of TH fluorescence signal in the dopaminergic neuron cluster in the olfactory bulb & telencephalon (referred to Fig. 8f). Data from three independent experiments are normalized to each DMSO sample, pooled together, and analyzed by Kruskall-Wallis non-parametric test with Dunn's multiple comparison test. **b,c,d,g,h,j** Data are displayed as box and whiskers plot showing the minimum and maximum points (whiskers), the first quartile, median and third quartile (box lines).

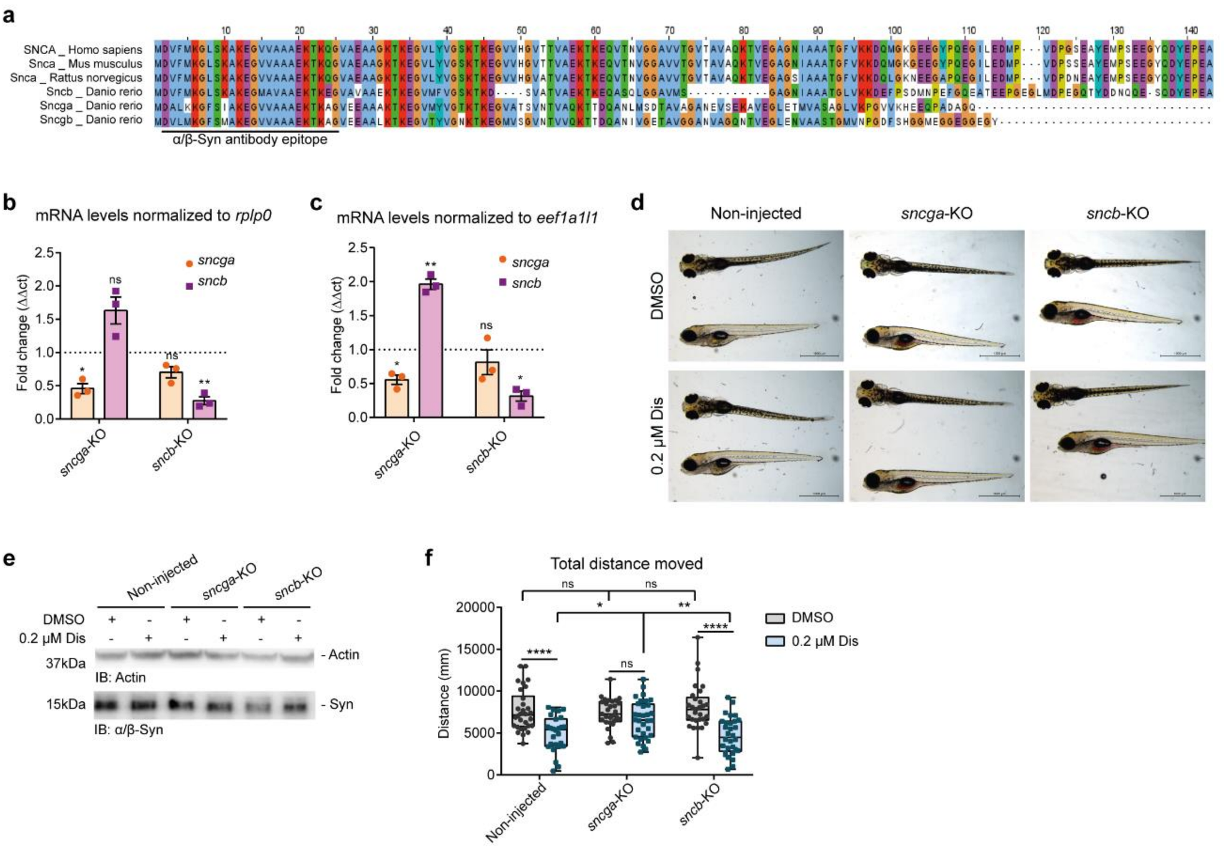

Supplementary Figure 8 Validation of zebrafish F0 KO larvae by CRISPR-Cas9 **a** Aminoacidic sequence alignment of SNCA protein of Homo sapiens, Mus musculus and Rattus norvegicus with the Sncb, Sncga and Sncgb of Danio rerio obtained by CLUSTALW and visualized by Jalview software. The sequence epitope 2-25 aa of the anti α/β-Syn antibody (SYSY) is reported. **b-c** Quantification of mRNA levels by qPCR of sncga and sncb in the heads of non-injected, sncga-KO and sncb-KO zebrafish larvae at 5 dpf. Gene expression levels were normalized for house-keeping genes *rplp0* or *eef1a1l1*. Data from three independent experiments are pooled together, normalized to non-injected samples (y=1 in the graphs) and analyzed by One-sample t-test (* p<0.05, ** p<0.001). Data are shown as Mean ± SEM. **d** Coronal and sagittal images of non-injected, sncga-KO and sncb-KO zebrafish larvae at 5 dpf, in DMSO- and 0.2 µM Disulfiram-exposed conditions. **e** Immunoblot with the anti-Synuclein and anti-Actin antibodies in lysates of zebrafish heads from each genotype, in both in DMSO- and 0.2 µM Disulfiram-exposed conditions. **f** Quantification of the total distance moved during the light-dark locomotion test of DMSO- and 0.2 µM Disulfiram-treated non-injected, sncga-KO and sncb-KO zebrafish (referred to Fig. 8i). Data from three independent experiments are analyzed by Two-way ANOVA with Sidak's multiple comparison test (* p<0.05, ** p<0.01, **** p<0.0001). Data are displayed as box and whiskers plot showing the minimum and maximum points (whiskers), the first quartile, median and third quartile (box lines).

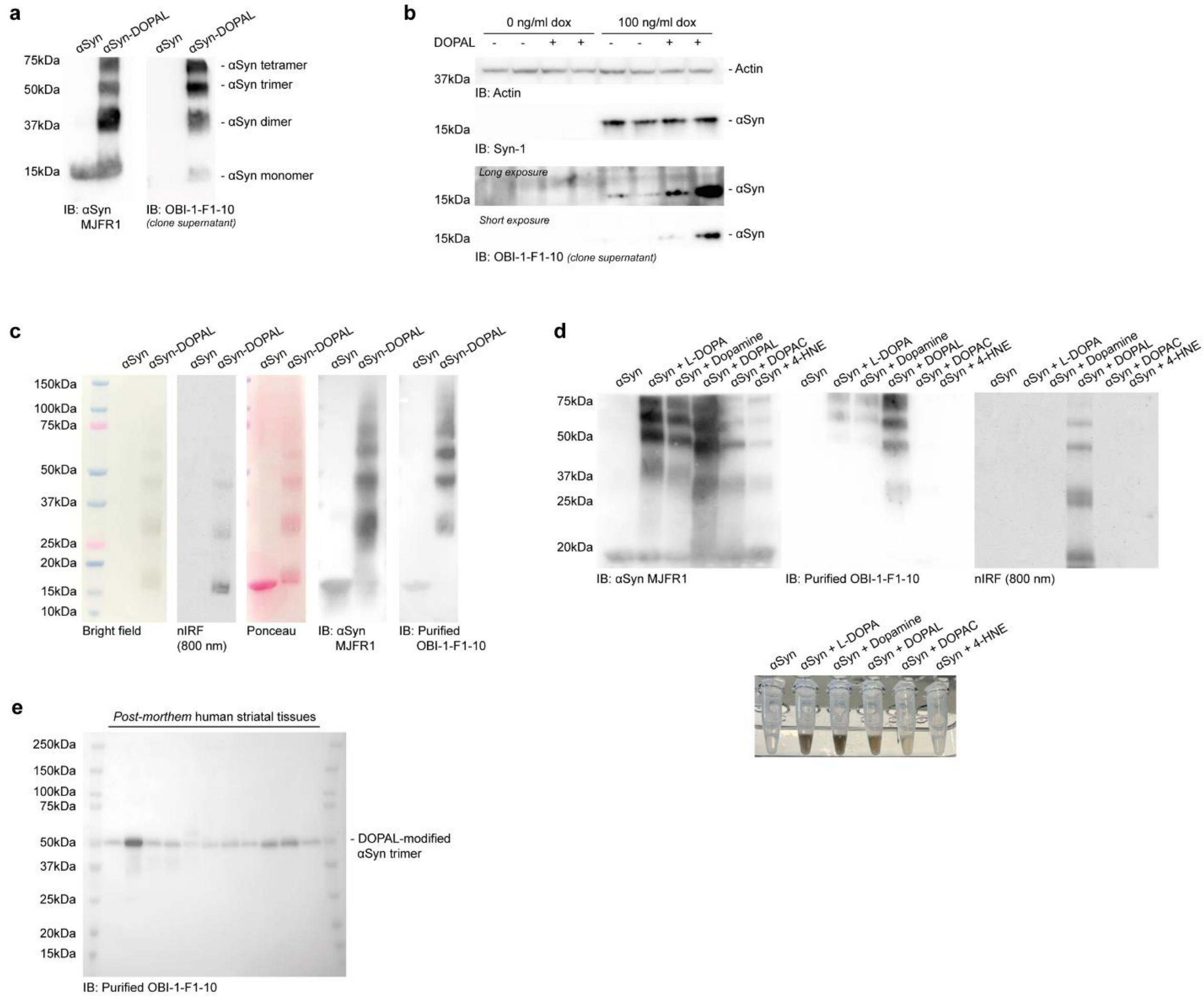

Supplementary Figure 9 Validation of anti DOPAL-modified αSynuclein OBI-1-F1-10 antibody **a** Western blot of 5 µg of recombinant human αSyn and 5 µg of DOPAL-modified αSyn oligomers generated by the incubation of 20 µM αSyn with 300 µM DOPAL in PBS at 37 °C – 350 rpm for 18 hours. The total αSyn (left image) was detected by the MJFR1 antibody (Abcam), while DOPAL-modified αSyn (right image) was detected by the OBI-F1-10 antibody present in the F1-10 clone supernatant. **b** Western blot of cell lysates of non-induced and 100 ng/ml dox-induced stable and inducible BE(2)-M17 cells, in untreated and 100 µM DOPAL-treated conditions. The αSyn overexpression was detected by the Syn-1 antibody (BD) while the OBI-F1-10 antibody (clone supernatant) presents the highest affinity for DOPAL-modified αSyn in induced DOPAL-treated cells. **c** SDS-PAGE (bright field acquisition), nIRF detection (at 800 nm), Ponceau staining and western blot with either MJFR1 antibody (total αSyn) or purified recombinant OBI-F1-10 antibody of recombinant human αSyn and 5 µg of DOPAL-modified αSyn oligomers. **d** SDS-PAGE, nIRF detection (at 800 nm) and western blot with either MJFR1 antibody (total αSyn) or purified recombinant OBI-F1-10 antibody of recombinant human αSyn and αSyn oligomers generated by the incubation for 12 hours at 37°C at 400 rpm with L-DOPA, dopamine, DOPAL, DOPAC and 4-HNE in a 1:15 αSyn:molecule ratio. The different brownish intensities of the reactions (in the bottom image) show the various degrees of catechol oxidation and polymerization. **e** Western blot of human brain lysates (striatal tissues). The incubation with purified recombinant OBI-F1-10 antibody displays a major band at 50 kDa, putatively corresponding to trimeric DOPAL-modified αSyn.

Supplementary Tables

Supplementary Table 1 Antigens synthetized for rabbit immunization to generate the OBI-1-F1-10 monoclonal antibody

Antibody name	Antigen sequences
Purified recombinant RabMab OBI-1-F1-10	Ac-C-Ahx-AAAGKTK(DOPAC)EGVLEA-amide
	Ac-AAAGKTK(DOPAC)EGVLEA-Ahx-C-amide

Supplementary Table 2 Sample demographics of the human cases used in this study

Cases	Sex (F/M)	Age (years)	***Post-mortem* dissection (hours)**
Healthy Control 1	M	69	49.3
Healthy Control 2	F	80	66.2
Healthy Control 3	M	83	35.4
Healthy Control 4	F	82	55
Healthy Control 5	M	71	42.3
Healthy Control 6	M	87	60
Idiopathic PD 1	M	78	22.3
Idiopathic PD 2	F	74	57.5
Idiopathic PD 3	M	80	62.5
Idiopathic PD 4	M	80	34.5
Idiopathic PD 5	F	76	44.2
Idiopathic PD 6	M	80	65

mRNA sequences of sncga and sncb genes, sgRNA sequences and primers for qPCR

NM_001017567.2GenBank Graphics>NM_001017567.2
Danio rerio synuclein, gamma a (***sncga***),mRNA

TGGCAATCGCACTTACCACACTGGCAGTCAGAGCACACACAGCTCCATCCACCAGCTATAAGACGAGCACCAGATCCAGTGAGAGGACGTAAAGCCCAGCACTGAGCT
CCAGCATGGATGCACTGAAGAAGGGATTCTCCATAGCCAAAGA**GGG**AGTGGTGGCCGCCGCTGAGAAAACCAAGGCCGGGGTGGAGGAGG**CAGCCGCCAAAACCAAAG**
AGGGGGTCATGTATGTAGGTACAAAGACAAAGGAGGGCGTTGCTACAAGTGTAAACACAGTTGCCCAGAAAACAACTGACCAGGCAAATCTCATGAGTGATACTGCTG
TGGCTGGAGCCAAT**GAGGTGTCAGAGAAGGCAG**TGGAGGGGCTGGAGACTATGGTCGCCTCTGC**AGG**CCTCGTCAAACCGGGGGTGGTGAAGCACGAGGAGCAGCCAG
CAGACGCC**GGG**CAG**TAG**GCCGCCGTTGAACCGAATGTCCCATGATGCTCTGCTTCTTGCCTGAGAGAAGAGTTTATTAGCTGCTGAATATCATCATGTTAAACCTCTG
AAGAACAACAAATAATCTTCAACTTCAGAAAACAAACACACTCGTCTCTTGCTTTCTCCTCACAATCAGCCTTTACTTACCTCACGCGCCACATATAGATATACTGAA
ATAGTATTGTTGTGTAACACTATCTAGCTATGCAATATTAATAAGATATGTATAATGTGCGGTTCGTGTCATTCTCACATTTGTGCAGATTTCGTATGCATGTGCTTG
TGATTTGGAGATTTACATGAAGACAAAGTCGCCTTTCTTACCTGCCCTGAAACTGGAGGCTTTCTTACCTGACCAGTTTCCTCCACCCAGTGCAATGAGGTTAGAAAG
ATGATGATGATGGTGATGAGTTGGATACTTACTGTGAGCAAAGATACTATATAAAATATATAAAAAATTCCATTTAACAATTATATAAGTACTGTCAATGTAGTAACT
GTAGTAGACAGTTTGAAACAAGGAGTATGGAAAAAAAGATGTAATGATAGATGGTCCAAAAAAGATGAGCAACTGATGTCTGTAAAGTGTGGTTATAAAATGATGCTC
TTTTATGTTGTTAACATATAATATAAACAAAAATTGAAATATTATGTCAGAGCAGTTATTAGGTTGCATCATCAGCCTATAAGTCTGTTGTTTCACTTTAACTCGTCA
CGTTCATTTACTTGACTTATTGTTCCTTGGCTGAAAGCAGCCAATCTTTTGAATATTCCATCTGTAATAAATTCAGTTTTGTGAAAAAAAAAAAAAAAAAAAAAAA

** In red, the start and the stop codon as well as the last and the first nucleotides of each exon.*

Selected gRNAs:

Gene name	Chromosome	Gene direction (forward: +; reverse: -)	Exon-target	crRNA's spacer sequence	PAM sequence
sncga	Chr13	+	Exon 1	GGATTCTCCATAGCCAAAGA	GGG
sncga	Chr13	+	Exon 3	TCTCATGAGTGATACTGCTG	TGG
sncga	Chr13	+	Exon 4	AGGAGCAGCCAGCAGACGCC	GGG

Primers qPCR:
sncga FOR: **CAGCCGCCAAAACCAAAGAG** CG:55% Tm: 59.3°C 20 bp
sncga REV: **GAGAATGACACGAACCGCAC** CG:55% Tm: 59.3°C 20 bp

Amplicon: 159 bp
Genomic DNA amplification: 10019 bp

NM_200969.1GenBank Graphics>NM_200969.1
Danio rerio synuclein, beta (***sncb***), mRNA

AATTGAAGCACAAGGACGAAGAAGGTGCTTTAAAAAGAGGGGCCAAGATGGATGTTTTTATGAAGGGGCTTTCTAAA**GCTAAAGAAGGGATGGCAGTG**GCTGCAGAAA
A**AACCAAGGAAGGTGTTGCGGTGG**CCGCTGAGAAAACCAAGGAAGGCGTGATGTTTGTGGGCAGCAAGAC**CAAAGACAGCGTTGCAACAGTG**GCTGAGAAGACGAAGG
AGCAGGCATCTCAGCTGGGCGGAGCAGTGATGTCTGGAGCCGGAAA**CATCGCCGCCGCCACCGGCCTGG**TGAAGAAGGACGAATTCCCCAGTGACATGAACCCGGAGT
TTGGTCAGGAAGCCACTGAGGAGCCGGGAGAAGGTCTGATGGACCCTGAAGGACA**GACATACGACGACAACCAGCAGG**AGAGCCAGGATTATGAGCCCGAGGCGTAAA
AGCGTCACATTCCAAGCCCAAATCCAGCCAGCAGCACAAAAAGTTTATATCAATACCAAGTACAAATCCTAAACACAATAAAAACCTGTTGCAAAAAAAAAAAAAAA

Selected gRNAs:

Gene name	Chromosome	Gene direction (forward: +; reverse: -)	Exon-target	crRNA's spacer sequence	PAM sequence
Sncb	Chr14	+	Exon 2	AACCAAGGAAGGTGTTGCGG	TGG
Sncb	Chr14	+	Exon 4	CATCGCCGCCGCCACCGGCC	TGG
sncb	Chr14	+	Exon 5	GACATACGACGACAACCAGC	AGG

Primers qPCR:
sncb FOR: **GCTAAAGAAGGGATGGCAGTG** CG:52.4% Tm:59.8°C 21bp
sncb REV: **CAACGCTGTCTTTGGTCTTGC** CG:52.4% Tm: 59.8°C 21bp

Amplicon: 123 bp
Genomic DNA amplification: 10887 bp

Primers qPCR for house-keeping genes:

ribosomal protein, large, P0 _ NM_131580.2
rplp0 FOR: CTGAACATCTCGCCCTTCTC
rplp0 REV: TAGCCGATCTGCAGACACAC

Eucaryotic translation elongation factor 1 alpha _ NM_131263
eef1a1l1 FOR: TTCGAGAAGGAAGCCGCTG
eef1a1l1 REV: CAGCAACAATCAGCACAGCAC

Full uncropped gels and western blots (1)

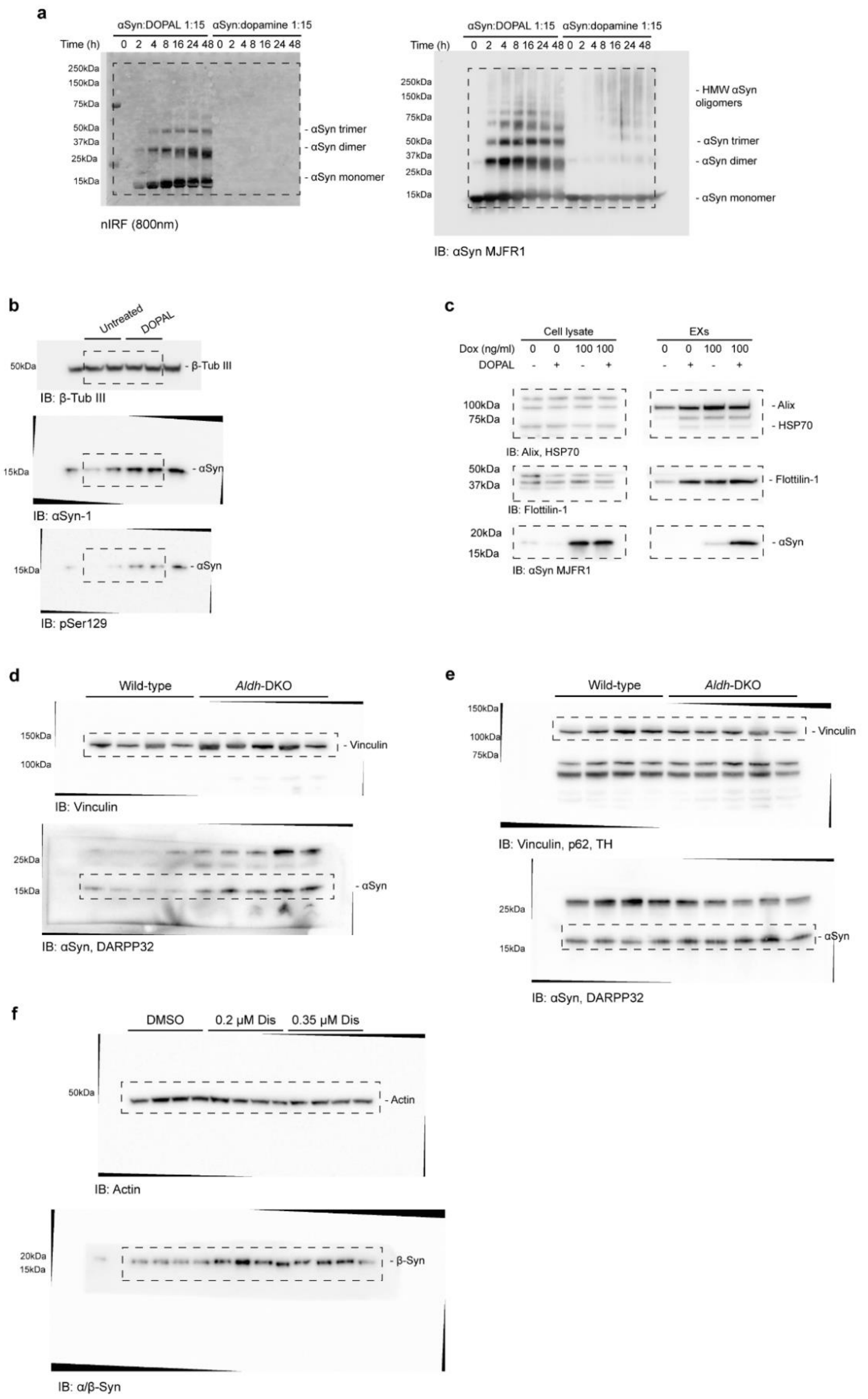

(**a**) Full uncropped nIRF image and western blot image from Fig. 1a. (**b**) Full uncropped western blot images from Fig. 1e. (**c**) Full uncropped western blot images from Fig. 4i. (**d-e**) Full uncropped western blot images from Fig. 7d-e. (**f**) Full uncropped western blot images from Fig. 8a. Dashed box represents the cropped images used in main figures.

Full uncropped gels and western blots (2)

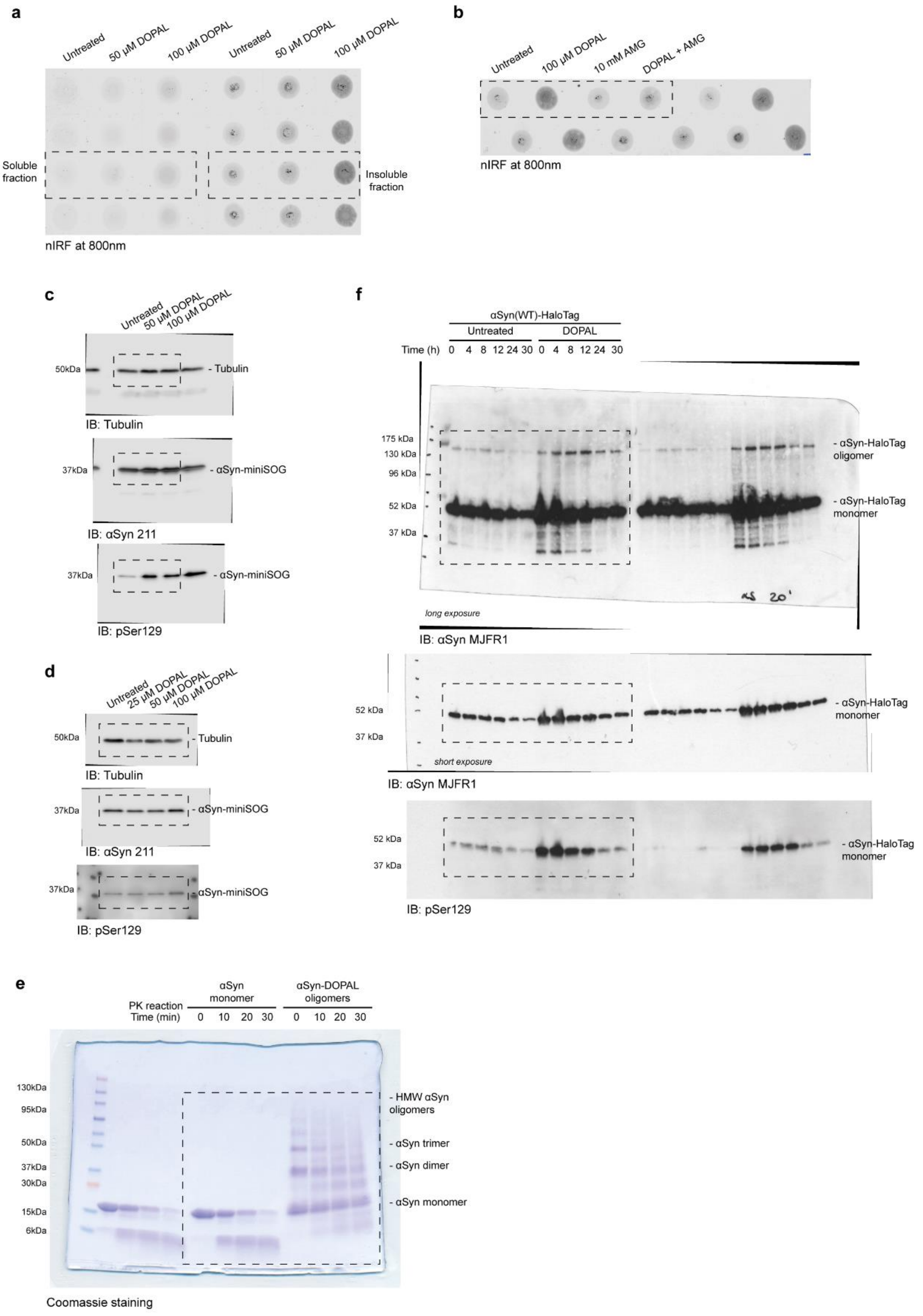

(**a-b**) Full uncropped nIRF image from Supplementary Fig. 1e,g. (**c-d**) Full uncropped western blot images from Supplementary Fig. 2d-e. (**e**) Full uncropped Coomassie stained gel from Supplementary Fig. 4a. (**f**) Full uncropped western blot images from Supplementary Fig. 4d. Dashed box represents the cropped images used in main figures.

Full uncropped gels and western blots (3)

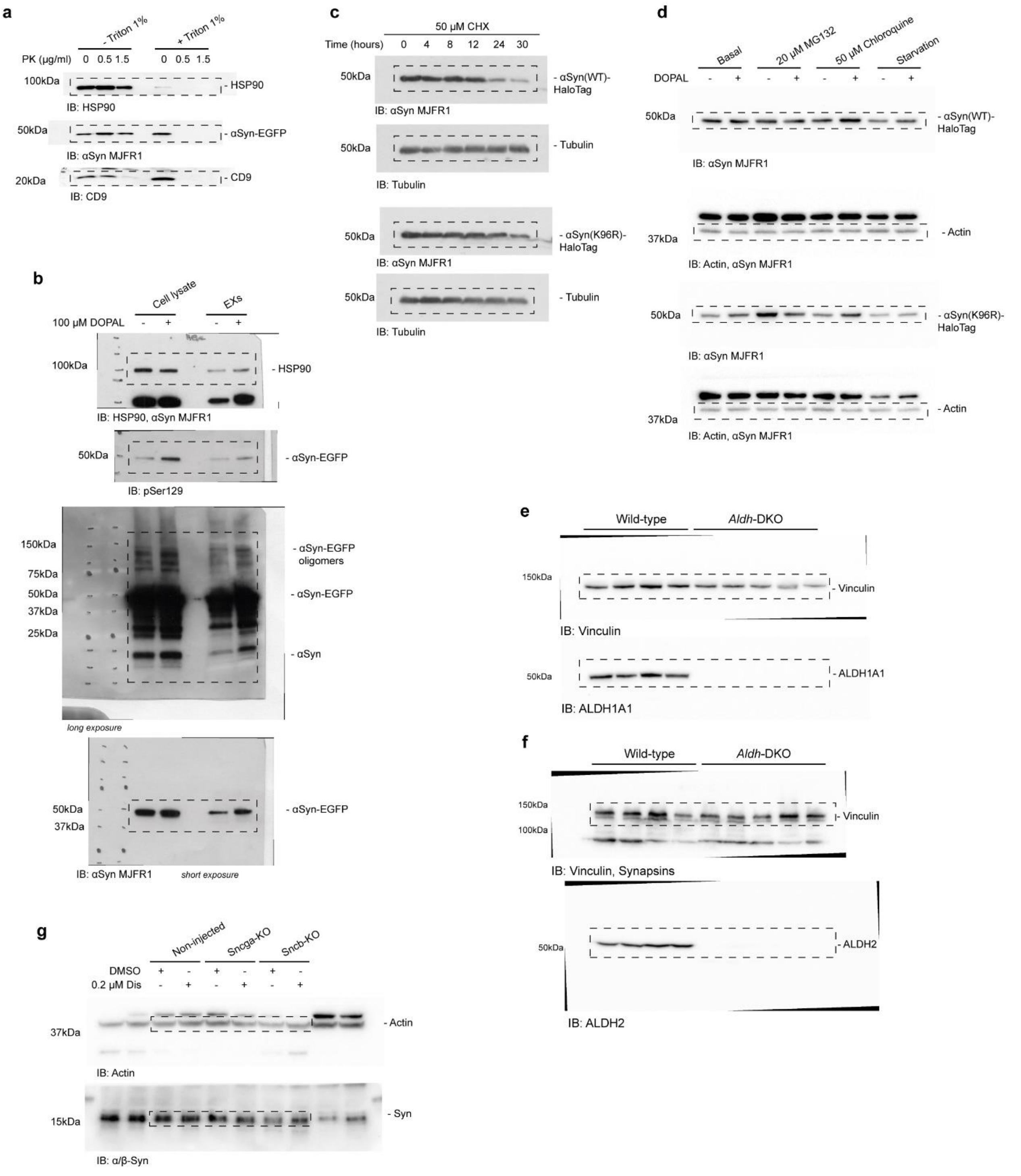

(**a-b**) Full uncropped western blot images from Supplementary Fig. 5d-e. (**c**) Full uncropped western blot images from Supplementary Fig. 5i. (**d**) Full uncropped western blot images from Supplementary Fig. 5k. (**e-f**) Full uncropped western blot images from Supplementary Fig. 7e-f. (**g**) Full uncropped western blot images from Supplementary Fig. 8e. Dashed box represents the cropped images used in main figures.

Full uncropped gels and western blots (4)

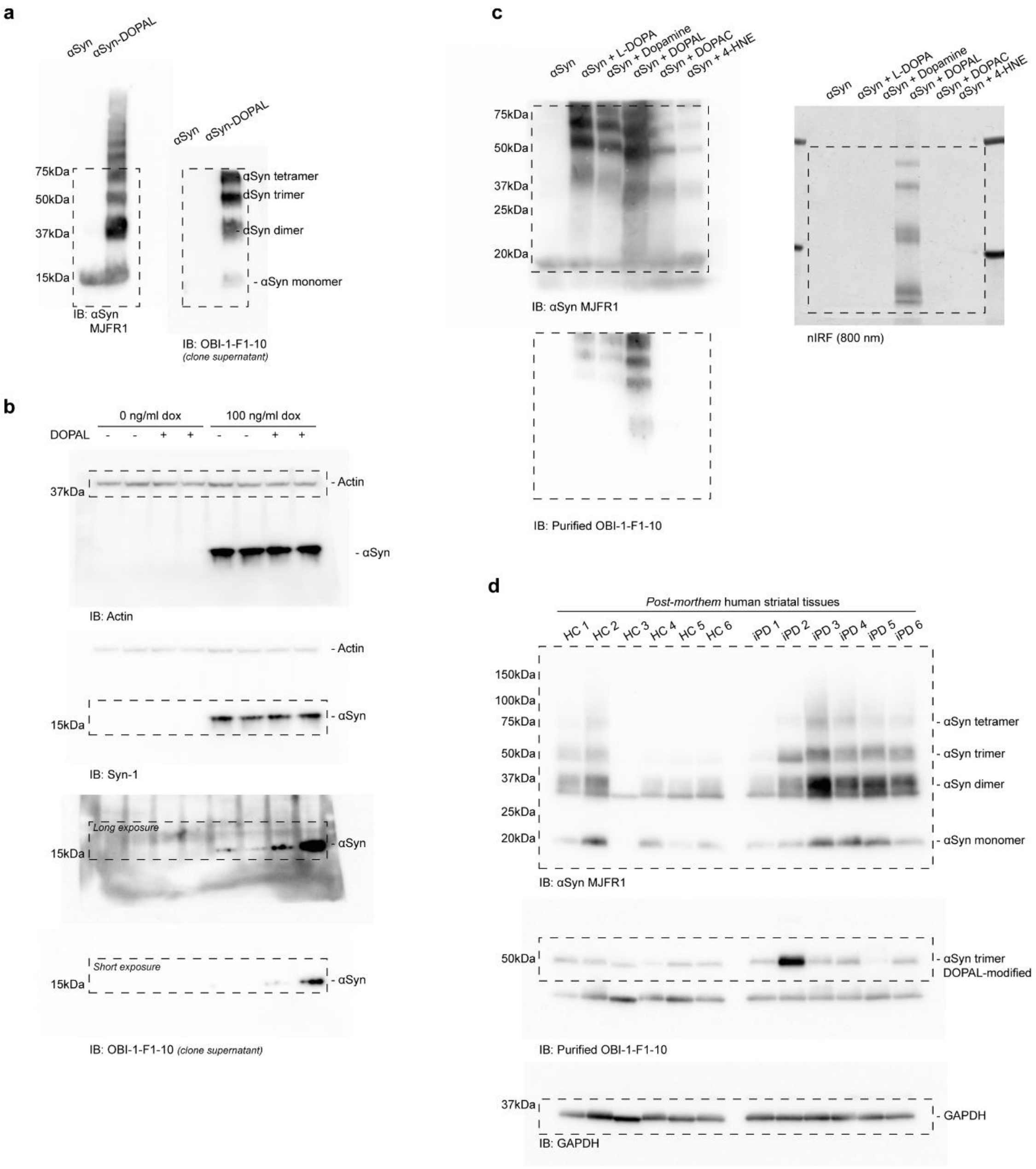

(**a-b**) Full uncropped western blot images from Supplementary Fig. 9a-b. (**c**) Full uncropped western blot and nIRF detection images from Supplementary Fig. 9d. (**d**) Full uncropped western blot images from Fig. 9a
