# Supplementary material for: DOPAL initiates αSynuclein-dependent impaired proteostasis and degeneration of neuronal projections in Parkinson’s disease
Source: NPJ Parkinsons Dis. 2023 Mar 25;9:42. doi: 10.1038/s41531-023-00485-1 (PMC10039907; doi:10.1038/s41531-023-00485-1)
Supplement: Supplementary file 4 — Reporting Summary [file 41531_2023_485_MOESM4_ESM.pdf]

nature portfolio

Corresponding author(s): Dr. Daniela Boassa
Prof. Luigi Bubacco

Last updated by author(s): Feb 2, 2023

Reporting Summary

Nature Portfolio wishes to improve the reproducibility of the work that we publish. This form provides structure for consistency and transparency in reporting. For further information on Nature Portfolio policies, see our Editorial Policies and the Editorial Policy Checklist.

Statistics

For all statistical analyses, confirm that the following items are present in the figure legend, table legend, main text, or Methods section.

n/a	Confirmed	
☐	☒	The exact sample size (*n*) for each experimental group/condition, given as a discrete number and unit of measurement
☐	☒	A statement on whether measurements were taken from distinct samples or whether the same sample was measured repeatedly
☐	☒	The statistical test(s) used AND whether they are one- or two-sided<br>*Only common tests should be described solely by name; describe more complex techniques in the Methods section.*
☐	☒	A description of all covariates tested
☐	☒	A description of any assumptions or corrections, such as tests of normality and adjustment for multiple comparisons
☐	☒	A full description of the statistical parameters including central tendency (e.g. means) or other basic estimates (e.g. regression coefficient) AND variation (e.g. standard deviation) or associated estimates of uncertainty (e.g. confidence intervals)
☐	☒	For null hypothesis testing, the test statistic (e.g. F, t, r) with confidence intervals, effect sizes, degrees of freedom and P value noted<br>*Give P values as exact values whenever suitable.*
☒	☐	For Bayesian analysis, information on the choice of priors and Markov chain Monte Carlo settings
☒	☐	For hierarchical and complex designs, identification of the appropriate level for tests and full reporting of outcomes
☒	☐	Estimates of effect sizes (e.g. Cohen's *d*, Pearson's *r*), indicating how they were calculated

Our web collection on statistics for biologists contains articles on many of the points above.

Software and code

Policy information about availability of computer code

Data collection	Custom code for MatLab to identify and measure fluorescence intensity of synaptic terminals in the live time-lapse confocal imaging.<br>Custom code for MatLab to measure inter-vesicle distances in the CLEM experiment.<br>Ethovision X.T. 8.5.614 software for the zebrafish behavioral analysis.
Data analysis	Excel, Fiji, GraphPad, OriginLab, MatLab, Imaris

For manuscripts utilizing custom algorithms or software that are central to the research but not yet described in published literature, software must be made available to editors and reviewers. We strongly encourage code deposition in a community repository (e.g. GitHub). See the Nature Portfolio guidelines for submitting code & software for further information.

Data

Policy information about availability of data

All manuscripts must include a data availability statement. This statement should provide the following information, where applicable:

- Accession codes, unique identifiers, or web links for publicly available datasets
- A description of any restrictions on data availability
- For clinical datasets or third party data, please ensure that the statement adheres to our policy

Most data generated or analyzed are included in the article and its Supplementary Information. Uncropped images of all gels and blots can be found in Supplementary File 1. Representative movies of the live time-lapse confocal imaging of αSyn-TimeSTAMP, as well as a representative track of zebrafish behavioral

analysis acquired at Danio Vision Observation chamber, the sgRNA sequeces for the CRISPR-Cas9 in zebrafish and the statistical analysis, are included in the Supplementary Information. Additional datasets and analyses, as well as relevant information are available from the corresponding authors upon requests.

Human research participants

Policy information about studies involving human research participants and Sex and Gender in Research.

Reporting on sex and gender	The gender of the healthy controls and PD patients whose post-mortem tissues have been analyzed in this study is reported in Table S2 in the Supplementary File 1.
Population characteristics	The age of the healthy controls and PD patients whose post-mortem tissues have been analyzed in this study is reported in Table S2 in the Supplementary File 1. Additional information on these subjects are detailed in Mammais et al., Neurobiol Dis 2013.
Recruitment	Post-mortem human striatal tissues from six idiopathic PD patients (iPD) and six age-matched healthy controls (HC) were obtained from Queen Square Brain Bank (London, UK).
Ethics oversight	Post-mortem human brains were collected under human tissue authority license #12198 to Dr. Rina Bandopadhyay.

Note that full information on the approval of the study protocol must also be provided in the manuscript.

Field-specific reporting

Please select the one below that is the best fit for your research. If you are not sure, read the appropriate sections before making your selection.

☒ Life sciences ☐ Behavioural & social sciences ☐ Ecological, evolutionary & environmental sciences

For a reference copy of the document with all sections, see nature.com/documents/nr-reporting-summary-flat.pdf

Life sciences study design

All studies must disclose on these points even when the disclosure is negative.

Sample size	In most experiments, sample size ranged between 5 to 50 data points. Data sets were analyzed without a normality assumption, therefore the statistical analysis among mean values was performed by two-tailed non-parametric Mann-Whitney test (two datasets) or by the two-tailed non-parametric Kruskall-Wallis test with the Dunn's multiple comparisons test (more than two datasets). For grouped analysis, the two-way ANOVA test with the Sidak's, Tukey's or Bonferroni's multiple comparison test was performed.
Data exclusions	Outliers were only excluded when data differed from the man value as twice the standard deviation.
Replication	In general, data were collected from at least n=3 independent experiments, with multiple technical replicates for each group. Within each biological replicate, data were normalized to the mean value of the untreated condition and plotted as fold-change variations of the treated samples compared to controls. Data from independent experiments were then pooled together. In some cases, experiments (immunofluorescence and CLEM) were performed only twice but a high number (n>20) of repeated measures were performed.
Randomization	Randomization was used when suitable with the experimental setup. Treatments were randomly assigned to cellular cultures and zebrafish individuals.
Blinding	Blinding was not used for data collection. Data analysis was conducted by algorithms and automated softwares to be as unbiased as possible.

Reporting for specific materials, systems and methods

We require information from authors about some types of materials, experimental systems and methods used in many studies. Here, indicate whether each material, system or method listed is relevant to your study. If you are not sure if a list item applies to your research, read the appropriate section before selecting a response.

Materials & experimental systems

n/a	Involved in the study
☐	☒ Antibodies
☐	☒ Eukaryotic cell lines
☒	☐ Palaeontology and archaeology
☐	☒ Animals and other organisms
☒	☐ Clinical data
☒	☐ Dual use research of concern

Methods

n/a	Involved in the study
☒	☐ ChIP-seq
☒	☐ Flow cytometry
☒	☐ MRI-based neuroimaging

Antibodies

Antibodies used

Immunofluorescence: mouse anti-pSer129 81/A (825702, BioLegend), mouse anti-Syn-1 (610787, BD), chicken anti-β-Tubulin III (302 306, SYSY), rabbit anti-αSyn pSer129 EP1536Y (ab51253, Abcam), mouse anti-aggregated αSyn SynO2 (847601, BioLegend), rabbit anti-aggregated αSyn MKFR14-6-4-2 (ab209538, Abcam), mouse anti-Bassoon (ab82958, Abcam), rabbit anti-VAMP2 (gifted by Prof. Montecucco's lab, UNIPD), rat anti-LAMP1 [1D4B] (ab25245, Abcam), rabbit anti-p62 (ab109012, Abcam), mouse anti-Ubiquitin (P4D1 sc-8017, Santa Cruz Biotech.), rabbit anti-TH (AB152, Millipore), rabbit anti-ALDH1A1 (GTX123973, GeneTex), rat anti-DAT (sc-32258, SCBT); goat anti-mouse-Alexa Fluor 488 (A11029, Invitrogen), goat anti-mouse-Alexa Fluor 568 (A11004, Invitrogen), goat anti-rabbit-Alexa Fluor 488 (A11034, Invitrogen), rabbit-Alexa Fluor 568 (A11036, Invitrogen), goat anti-rat-Alexa Fluor 647 (A21247, Invitrogen), goat anti-chicken-Alexa Fluor 647 (A21449, Invitrogen).

Western blot: mouse anti-α-Tubulin (T6074, Sigma-Aldrich), mouse anti-αSyn 211 (S5566, Sigma-Aldrich), mouse anti-αSyn pSer129 81/A (825702, BioLegend), mouse anti-β-Tubulin III (T8578, Sigma-Aldrich), mouse anti-Syn-1 (610787, BD Transduction Laboratories), rabbit anti-αSyn pSer129 EP1536Y (ab51253, Abcam), rabbit anti-αSyn MJFR1 (ab138501, Abcam), mouse anti-β-Actin (A1978, Sigma-Aldrich), rabbit anti-Alix (ABC40, Millipore), mouse anti-HSP90 (SPA830, ENZO Life Sciences), mouse anti-HSP70 (SPA810, ENZO LIfe Sciences), mouse anti-Flotillin-1 (610822, BD Transduction Laboratories), rabbit anti-CD9 (ab92726, Abcam), rabbit anti-Vinculin (AB6039, Millipore), rabbit anti-ALDH1A1 (GTX123973, GeneTex), rabbit anti-ALDH2 (GTX101429, GeneTex), muse anti-GAPDH (CSB-MA000195), rabbit anti-α/β.Synuclein (1280 002, SYSY), rabbit anti-DOPAL-modified aSyn (OBI-1-F1-10, generated by UNIPD in partnership with Abcam); goat anti-rabbit-HRP (A9169, Sigma-Aldrich); goat anti-mouse-HRP (A9044, Sigma-Aldrich).

Validation

Most of antibodies used are commercially available and validated in existing literature and/or from manufacturer. For the rabbit anti-DOPAL-modified aSyn OBI-1-F1-10, generated by UNIPD in partnership with Abcam, the validation is reported in Supplementry File 1, Figure S9.

Eukaryotic cell lines

Policy information about cell lines and Sex and Gender in Research

Cell line source(s)	Primary mouse and rat cortical neurons isolated from Sprague-Daley rat, C57BL/6LOlaHsd and C57BL/6JRccHsd mice. Neuroblastoma-derived BE(2)-M17 cells (ATCC CRL-2267).
Authentication	BE(2)-M17 cell line authentication and characterization were previously published in Filograna et al., PLoS ONE 2012.
Mycoplasma contamination	Cell lines were tested for mycoplasma contamination by Hoechst staining and fluorescence imaging.
Commonly misidentified lines (See ICLAC register)	No commonly misidentified cell lines were used.

Animals and other research organisms

Policy information about studies involving animals; ARRIVE guidelines recommended for reporting animal research, and Sex and Gender in Research

Laboratory animals	Primary cortical neuron cultures were isolated from post-natal day 2 Sprague-Daley rat, and post-natal day 0 C57BL/6LOlaHsd and C57BL/6JRccHsd mice (regardless animal sex). Male mice with homozygous deletions of both Aldh1a1 and Aldh2 genes (double knock-out mice) on a C57BL/6 background previously generated were sacrificed between 11 and 12 months of age. Wild-type zebrafish were sacrificed at day post fertilization 5 or 6.
Wild animals	N/A
Reporting on sex	N/A
Field-collected samples	N/A
Ethics oversight	N/A

Note that full information on the approval of the study protocol must also be provided in the manuscript.
