# Supplementary material for: DOPAL initiates αSynuclein-dependent impaired proteostasis and degeneration of neuronal projections in Parkinson’s disease
Source: NPJ Parkinsons Dis. 2023 Mar 25;9:42. doi: 10.1038/s41531-023-00485-1 (PMC10039907; doi:10.1038/s41531-023-00485-1)
Supplement: Supplementary file 9 — Supplementary Files [file 41531_2023_485_MOESM9_ESM.docx]

**Supplementary Information**

**Supplementary File 1** Supplementary Figures 1-9; Supplementary Tables 1-2; mRNA sequences of *sncga* and *sncb* genes, sgRNA sequences and primers used for the generation and validation by qPCR of *sncga*-KO and *sncb*-KO zebrafish larvae; Full uncropped gels and western blots (.pdf)

**Supplementary** **File 2** Representative video of live-cell time-lapse confocal imaging of αSyn-TimeSTAMP-YFP-miniSOG in untreated primary rat cortical neurons. Related to Figure 2d (.mp4)

**Supplementary** **File 3** Representative video of live-cell time-lapse confocal imaging of αSyn-TimeSTAMP-YFP-miniSOG in 100 µM DOPAL-treated primary rat cortical neurons. Related to Figure 2e (.mp4)

**Supplementary** **File 4** Representative video of live-cell time-lapse confocal imaging of αSyn-TimeSTAMP-YFP-miniSOG in 5 µg/ml Nocodazole-treated primary rat cortical neurons. Related to Figure 4c (.mp4)

**Supplementary** **File 5** Representative video of live-cell time-lapse confocal imaging of αSyn-TimeSTAMP-YFP-miniSOG in 5 µg/ml Nocodazole + 100 µM DOPAL-treated primary rat cortical neurons. Related to Figure 4d (.mp4)

**Supplementary** **File 6** Raw data of zebrafish behavioral study. Related to Figures 7c,d,e and Figure S7g,h,i (.xlsx)

**Supplementary File 7** Detailed statistical analysis of each graph presented in the main and supplementary figures (.xlsx)
